# Dissecting the genome-wide genetic variants of milling and appearance quality traits in rice

**DOI:** 10.1093/jxb/erz256

**Published:** 2019-05-30

**Authors:** Gopal Misra, Roslen Anacleto, Saurabh Badoni, Vito Butardo, Lilia Molina, Andreas Graner, Matty Demont, Matthew K Morell, Nese Sreenivasulu

**Affiliations:** 1 International Rice Research Institute, DAPO, Metro Manila, Philippines; 2 Leibniz Institute of Plant Genetics and Crop Plant Research (IPK), Seeland OT Gatersleben, Germany; 3 CSIRO Agriculture and Food, Australia

**Keywords:** Genome-wide association studies, grain chalkiness, head rice yield, market survey, multi-locus GWAS, whole-genome resequencing

## Abstract

Higher head rice yield (HRY), which represents the proportion of intact grains that survive milling, and lower grain chalkiness (opacity) are key quality traits. We investigated the genetic basis of HRY and chalkiness in 320 diverse resequenced accessions of *indica* rice with integrated single- and multi-locus genome-wide association studies using 2.26 million single-nucleotide polymorphisms. We identified novel haplotypes that underly higher HRY on chromosomes 3, 6, 8, and 11, and that lower grain chalkiness in a fine-mapped region on chromosome 5. Whole-genome sequencing of 92 IRRI breeding lines was performed to identify the genetic variants of HRY and chalkiness. Rare and novel haplotypes were found for lowering chalkiness, but missing alleles hindered progress towards enhancing HRY in breeding material. The novel haplotypes that we identified have potential use in breeding programs aimed at improving these important traits in the rice crop.

## Teaser

Genetic regions in the rice genome are identified that affect the proportion of grains that remain intact after milling (the head rice yield) and the degree of chalkiness in the grain.

## Introduction

The annual global production of rice (*Oryza sativa*) grown in paddy systems amounts to 730 mt, resulting in 484 mt of polished grains after milling to remove the husk and the bran layers. As a staple food, rice provides at least 20% of the daily caloric requirement for ~3.5 billion people worldwide. To meet the future demands of a growing global population, it is estimated that annual milled rice production needs to increase by additional 105 mt by 2035. Despite the development of newer, high-yielding varieties, many farmers continue to plant the popular ‘mega-varieties’ produced between 1966–1985 (Peng *et al.*, 2004). The lack of sustained adoption of more recently released modern varieties suggests that we not only need to overcome yield stagnation in new varieties (Ray *et al.*, 2012), but that we also need to match the quality benchmarks of the mega-varieties in terms of premium grain quality traits. These include a high head rice yield and a low degree of chalkiness, which affects texture (Laborte *et al.*, 2015).

Head rice yield (HRY) is the proportion of ‘intact’ grains that remain after milling, and comprises grains that are at least 75% of the original length (Cnossen *et al.*, 2003). HRY is an important quality trait that can fetch a higher price for farmers due to the net increase in milled whole-grain (unbroken) yield, especially when this is combined with a lower proportion of chalky grains. Dehulling and milling are important steps in the post-harvest processing of paddy rice, during which the grains are susceptible to fissuring and breakage (Buggenhout *et al.*, 2013). The mechanical stress imposed can result in up to 50% loss in HRY. Lower intrinsic grain strength leads to higher breakage during grain processing and this is measured as the fracture strength, which is defined as the force that can be tolerated by a grain before it cracks (Buggenhout *et al.*, 2013). There are many factors that affect HRY, including (i) post-harvest practices related to the process of grain drying, (ii) the grain moisture content at harvest, which affects the transition from a more diffusible, rubbery state to a less-diffusible, glassy starch that leads to grain fissuring (Cnossen *et al.*, 2003; Siebenmorgen *et al.*, 2007), (iii) the negative effect of high night temperatures during seed filling, and (iv) genetic factors (Cooper *et al.*, 2008; Sreenivasulu *et al.*, 2015). Although several milling quality traits that exhibit low heritability have been mapped in a subpopulation of *japonica* species (Nelson *et al.*, 2011, 2012; Pinson *et al.*, 2013), no major quantitative trait loci (QTLs) and genes that influence HRY have been cloned in *indica* germplasm. The dissection of the genetic basis of HRY is important for understanding how kernel susceptibility to breakage can be reduced, and milling quality improved, through marker-assisted breeding in the long, slender grains of *indica* rice.

Another important grain quality trait is chalkiness, which is generally characterized as an opaque white discoloration in the translucent endosperm caused by the creation of air spaces between irregularly shaped starch granules (Butardo and Sreenivasulu, 2019). An increased proportion of chalky grains lowers the appearance quality of rice, and is also believed to lower the milling quality by increasing the incidence of grain breakage (Del Rosario *et al.*, 1968; Lisle *et al.*, 2000; Wang *et al.*, 2007; Cooper *et al.*, 2008). Thus, chalkiness has negative impacts on the market value of rice. Grain chalkiness is influenced by genetic and environmental factors (Lisle *et al.*, 2000; Yamakawa *et al.*, 2007; Lanning *et al.*, 2011; Siebenmorgen *et al.*, 2013; Zhao and Fitzgerald, 2013). Chalk is a complex and polygenic quantitative trait and several fine-mapped target genes have been shown to influence it, including pyruvate orthophosphate dikinase (Kang *et al.*, 2005), starch synthase IIIa (Fujita *et al.*, 2007), cell wall invertase (Wang *et al.*, 2008), UDP-glucose pyrophosphorylase (Woo *et al.*, 2008), H^+^-translocating pyrophosphatase (*chalk5*) (Li *et al.*, 2014), and a transcriptional activator *GS9* (Zhao *et al.*, 2018).

Genome-wide association studies (GWAS) in combination with targeted-gene association studies (TGAS) have emerged as a two-pronged strategy that can assist in narrowing down the significant trait-specific gene(s) underlying QTLs and in revealing allelic variants that regulate complex grain-quality traits (Butardo *et al.*, 2017; Misra *et al.*, 2017). Here, we studied the genetic basis of HRY and chalkiness using combined GWAS (single- and multi-locus) and gene-set analysis by employing 2.26 million single-nucleotide polymorphisms (SNPs) from resequencing data of 320 *indica* rice accessions. This approach helped to identify key candidate genes that influence the complex genetic architecture of HRY under controlled drying as well as under moisture-stressed conditions. We identified novel haplotypes/allelic variants for enhanced HRY and reduced grain chalkiness. Our findings will assist the future development of high-yielding elite germplasm with superior grain quality and thus contribute to improving the livelihoods of farmers in many developing countries.

## Materials and methods

### Plant material and HRY analysis from controlled and moisture-stressed drying conditions

A diversity panel of 320 *Oryza sativa* subsp. *indica* accessions (262 landraces and 58 improved lines) were selected from 3000 resequenced rice genomes (The 3000 Rice Genomes Project, 2014; Wang *et al.*, 2018) (Supplementary Table S1 at *JXB* online). The accessions were grown in field plots of 12.5×50 m in three replicated randomized blocks during the 2014 wet season and during the 2015 dry season at the Experimental Station of the International Rice Research Institute (IRRI), Laguna, Philippines (14°N, 121°E). Standard crop management procedures were applied. Upon reaching seed maturity, the individual plots were harvested manually and hand-threshed at an optimum seed moisture content (MC) of 22–24%. For the controlled drying treatment, grains were then subjected to flat-bed drying to achieve 12–14% MC and subsequently placed in a seed storage room maintained at 18 °C before being milled.

To further test the effect of moisture stress on HRY, dried harvested grains (~200 g samples) with 12–14% MC were soaked in reverse osmosis water for 60 min at ambient temperature until the MC of duplicate samples reached the optimum level of 22–24%. The samples were then placed inside a pre-heated oven at 45 °C until they returned to 12–14% MC. The samples were then placed at room temperature inside an air-tight plastic ziplock bag for 1 h, after which HRY was determined.

Subsamples of grains (125 g, three replicates) from the control and moisture stress treatments were dehulled using a Satake dehuller (model THU-35A, Japan) with the settings optimized for the different grain shapes. Each sample was then milled using Grainman mill (model 60-200-60-DT, Miami) for 45 s. Head rice was separated from broken kernels using a post-harvest drum type-sizing machine (Satake rice machine type TRG with three different layers) and HRY (%) was calculated as the mass proportion of milled rice retained as head rice after complete milling. The stability value was calculated by dividing the HRY value under stress by the HRY value under controlled condition. The HRY evaluation was conducted according to the ISO 17025 guidelines.

An additional 300 *indica* lines from the diversity panel with SNP-based genotyping data of 700K (McCouch *et al.*, 2016) were planted at the IRRI experimental station in 1-m^2^ plots in a six rows by six hills per row configuration with 20 cm between hills and rows. The plots were arranged in three replicated randomized blocks, and plants were grown during the dry seasons in 2013 and 2014. To mimic the usual farming practice of sun-drying, samples of 2 kg of intact straw and panicles were packed in large net bags and subjected to sun-drying followed by drying under shade until they attained 12–14% MC. The samples were then evaluated for HRY and other milling quality parameters as described above.

### Phenotyping for grain chalkiness

Dried samples of grain at 12–14% MC (50 g, three replicates) of each cultivar were used to determine percentage chalkiness. Chalkiness was estimated using a SeedCount SC5000 Image Analyser (Next Instruments, NSW, Australia) as percentage grain chalkiness (PGC) using optimized methods. Grains were scattered randomly onto the sample tray of the instrument, and after scanning PGC was estimated using the in-built software after calibration according to the manufacturer’s instructions.

### Performance of historical breeding lines and their whole-genome sequencing

We evaluated a total of 106 breeding lines developed by IRRI between 1966–2015 for HRY and chalkiness. Briefly, the lines were grown in field plots during wet (2015) and dry (2016) seasons according to standard agronomic practice (Laborte *et al.*, 2015). Grain was harvested at 22–24% MC and subjected to standard drying conditions as described above. The dried samples were evaluated for HRY and chalkiness as described above.

Genomic DNA extracted from leaf samples of 92 breeding lines were subjected to whole-genome sequencing using the HiSeq X10 platform (Illumina) and 150-bp pair-end reads were generated at ~30× coverage. After completion of the sequencing process, the raw reads were filtered to remove adaptor sequences and low-quality reads.

### Survey of farmers for desirable traits in the rice crop

IRRI conducted in-depth market surveys during 2015–2016 involving 886 rice farmers from Bangladesh, the eastern part of India, Cambodia, and the Philippines (Ynion *et al.*, 2015, 2016; Maligalig, 2018). An interactive tablet application was utilized to assess the priorities of traits for their improvement that farmers would consider before they would replace their most popular current varieties with new ones. The application enabled farmers to design an ideal future variety profile by allocating a fixed investment fund across a portfolio of 11 traits, divided into market-related traits (slenderness, aroma, stickiness, and HRY) and environment-related traits (lodging tolerance, insect resistance, disease resistance, abiotic stress tolerance, reduction of shattering, earliness, and straw digestibility).

### Genome-wide association studies and gene-set analysis

A resequenced diversity panel consisting of 324 *indica* accessions was used for calling of genome-wide SNPs against reference genomes of *japonica* (cv. Nipponbare) and *indica* (MH63 and ZS97) (Zhang *et al.*, 2016). An efficient mixed-model association expedited (EMMAX) approach was used for GWAS (Kang *et al.*, 2010). HRY and PGC data for the different seasons were transformed using the Python software WarpedLMM (https://pypi.org/project/WarpedLMM/) to adjust the phenotype distribution. Genotype files were filtered prior to running the association analysis. Individual genotypes and SNPs were retained with a missing rate of not more than 5% and further filtered for a minor allele frequency of at least 5%. This plink2-based filtering step (https://www.cog-genomics.org/plink/2.0/) resulted in a final set of 320 *indica* lines that accounted for 2 260 030 SNPs against the Nipponbare genome, 802 117 SNPs against the MH63 genome, and 450 604 SNPs against the ZS97 genome. For a diversity panel of 300 lines, we used the previously published 700K SNP data from McCouch *et al.* (2016).

The statistical model used in EMMAX is a variance component model detailed in Kang *et al.* (2010). Four principal components (PCs), explaining ~14% of total variation, were used as covariates in GWAS after plotting 20 PCs in a scree plot. To determine the PC, a set of 2.26 million SNPs detected against the Nipponbare reference was utilized and the analysis was performed using the SNPRelate package in the R platform (http://dx.doi.org/10.18129/B9.bioc.SNPRelate). The data from the different seasons were used to check the reproducibility of the results. After running the GWAS on different seasons, we further analysed the results from *indica* resequenced lines. SNPs were tagged based on linkage disequilibrium (LD) from the set of SNPs with –log_10_*P*>5 for detailed analysis of HRY and PGC. The complete parameters used for clumping (in plink2) were adapted from Misra *et al.* (2017), except that we used the --clump-p1 of 1e^−7.6^. Haploview (Barrett *et al.*, 2005) was used to construct LD-blocks, and plots were then generated using standard procedures (Misra *et al.*, 2017). All genomic positions and gene annotations were based on the Nipponbare (MSUv7), MH63, and ZS97 reference genomes.

The multi-locus GWAS was run to verify the peaks identified by single-locus GWAS. Different multi-locus (ML) GWAS methods, namely FASTmrEMMA (Wen *et al*., 2018), mrMLM (Wang *et al.*, 2016a), and ISIS EM-BLASSO (Tamba *et al.*, 2017), were followed as detailed in Misra *et al.* (2017). We performed a gene-set analysis based on the MSU7 annotation using the publicly available MAGMA software (de Leeuw *et al.*, 2015) for those genes implicated by the SNPs that met the Bonferroni threshold.

### Validating the haplotype blocks

Haplotype blocks with distinguished phenotype ranges were validated using the SNP-seek database (Alexandrov *et al.*, 2015) where SNP data for 3000 rice genomes are archived.

### Collinearity overlays with HRY and chalkiness genetic regions

The translated protein sequences obtained from reference genomes of Nipponbare, MH63, and ZS97 were used for deriving synteny. All-to-all blastP was performed for each pair of reference protein sequences using the NCBI-BLAST-2.2.28+ tool (Altschul *et al.*, 1990). A stringent filtering criterion of e^−20^ was used in the BLAST alignment of protein sequences between *japonica* (Nipponbare) and *indica* (ZS97). The longest proteins were selected in cases where splice variants were present. Collinearity was then established using MCScanX (Wang *et al.*, 2012) with the threshold of 10 genes constructing each collinear block between *indica* and *japonica*, whilst 12 genes were used between the two *indica* reference genomes. On the basis of these outcomes, colinearity was identified and represented in the form of blocks using Circos (Krzywinski *et al.*, 2009) that overlaid with the genomic region regulating HRY and PGC that had been identified through GWAS.

## Results

### Survey of farmers for importance of HRY

**Fig. 1.**
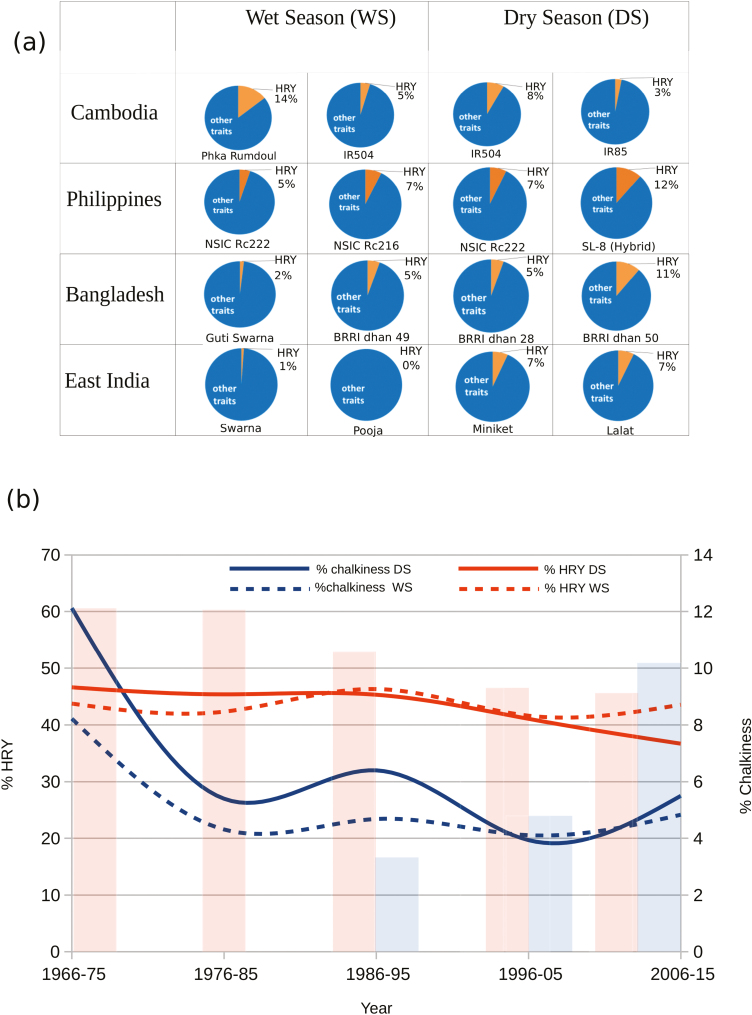
Survey of farmers for importance of head rice yield (HRY) in mega-varieties in the Philippines, Bangladesh, Cambodia and Eastern India, and evolution of grain chalkiness and HRY in breeding lines released by IRRI during the period 1966–2015. (a) Investment share assigned by farmers in HRY relative to other traits as elicited through an investment exercise (see Methods). (b) Trends in the genetic gain for % chalkiness and % HRY observed in dry seasons (DS) and wet seasons (WS) for the 106 breeding lines developed at IRRI between 1966–2015. The decrease in the HRY was observed to be more prominent during dry seasons, whilst chalkiness showed a similar trend in DS and WS. In the bar plots, orange bars represent HRY and blue bars represent % chalkiness , as adapted from Laborte *et al.* (2015), and show a similar trend in the Philippines and corroborates our results.

Given a fixed investment fund to be divided between 11 market- and environment-related traits, the share that farmers invested in HRY was in the range 0–14%, depending on the geographic location, the season, and the rice variety that was currently being grown (Fig. 1a). Slender grain varieties such as Phka Rumdoul in Cambodia (14%), SL-8 in the Philippines (12%), BRRI Dhan-50 in Bangladesh (11%), and Miniket in East India (7%) attracted the highest investment in HRY, and some of these varieties are typically grown in the dry season. Data for 106 breeding lines developed at IRRI during 1966–2015 analyzed by Laborte *et al.* (2015) indicate that over several generations of introduced varieties, HRY has declined (Fig. 1b, bar plot, orange bars), while chalkiness has dramatically increased (blue bars). This is consistent with farmers’ prioritization of investment in HRY for high-yielding varieties such as NSIC Rc222 and hybrids such as SL-8. These trends argue for more investment in research focused on unravelling the genetics of HRY.

### Performance of HRY and chalkiness in historical breeding lines

We evaluated a total of 106 breeding lines developed by IRRI between 1966–2015 for HRY and chalkiness by growing them together in dry and wet seasons and subjecting the grain to conditions of controlled drying. While chalkiness was found to be high in varieties released between 1966–1975, it was substantially lower in modern varieties released between 2006–2015 (Fig. 1b, trend line). This reduction was due to successful selection made against chalkiness through efficient image-based phenotyping. In contrast, HRY of varieties released during the early post-Green Revolution was significantly higher (~60% HRY for 1966–1975 shown in bar plot) compared to modern varieties released between 2006–2015 (~45% HRY). For varieties released between 2006–2015, HRY was found to be significantly lower in the dry season that in the wet season (Fig. 1b, trend line).

### Evaluation of a diversity panel for genetic variation in HRY and chalkiness

**Fig. 2.**
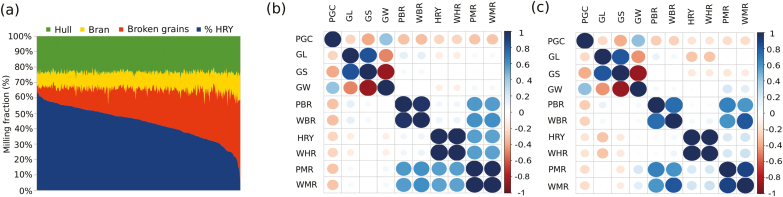
Phenotypic variation for milling quality traits in the diversity panel of 320 *indica* lines and their correlations with grain chalkiness. (a) Diversity for milling potential showing a large degree of variation for HRY (*y*-axis). The labels at the top indicate the stages during the milling process. Milling fractions include the hull (outer seed coat), the removal of which during the dehulling process give rise to brown rice. Removal of bran fraction results in milled rice (broken + intact grains). The intact grains represent the % HRY. (b, c) Correlations among different milling quality traits, grain chalkiness, and grain dimension attributes in (b) control and (c) moisture stress conditions. Blue and red indicate positive and negative correlations, respectively, and the size of the circles and intensity of the colour represent the degree of correlation. PGC, % grain chalkiness; GL, grain length; GW, grain weight; PBR, % brown rice; WBR, weight brown rice; HRY, head rice yield (%); WHR, weight head rice; PMR, % milled rice; WMR, weight milled rice. In both control and stress conditions, PGC showed negative correlations with the all of milling quality traits including the HRY and WHR, whereas showed positive correlation with GW.

As we did not observe much genetic variation for HRY in existing breeding lines, we examined a diversity panel of 320 resequenced *indica* lines. We phenotyped a wide range of milling and appearance qualities traits, namely percent HRY, weight of head rice (WHR), percent brown rice (PBR), weight of brown rice (WBR), percent milled rice (PMR), weight of milled rice (WMR), and PGC. The diversity panel showed a high degree of variation for HRY (6.22–63.8%) (Fig. 2a) with a broad-sense heritability of *H*^2^=0.88 for replicates grown in the 2015 dry season. Nevertheless, when the wet and dry season data were combined, the broad-sense heritability for HRY dropped to 0.56. PGC ranged from 0.07–100 with *H*^2^=0.90 for the dry season; when data from both seasons were combined, heritability remained high at *H*^2^=0.86. The other milling quality traits showed less variability (Supplementary Fig. S1 at *JXB* online). While HRY was moderately correlated with PMR and WMR (*r*^2^=0.54–0.55) across the 320 lines, a high level of correlation was observed between HRY and WHR (*r*^2^≥0.99). HRY was found to be poorly correlated with PBR and WBR (*r*^2^=0.09–0.10) (Fig. 2b), and also with traits related to grain size and shape (length, –0.13; width, –0.11; shape, –0.01). Moreover, PGC was negatively correlated with HRY (*r*^2^=–0.25), although with low significance. Thus, strategies employed towards lowering chalkiness alone might not contribute significantly to increase HRY, indicating that it is important to independently examine the underlying genetic variations that contribute to increasing HRY in the pre-breeding pool.

Susceptibility to grain breakage due to induced moisture stress was examined in the 320 lines. Notably, we observed a relatively lower mean HRY value of 34±0.71%, compared with 44 ±0.64% for controlled drying. The broad-sense heritability of the replicates for HRY under moisture stress was *H*^2^=0.84. Grain length showed negative correlations with HRY and WHR in the moisture stress treatment (*r*^2^=–0.30 for both), with a stronger relationship compared to control conditions (*r*^2^=–0.13) (Fig. 2c). These results suggested that variable moisture content in the grain makes long-grain types more susceptible to fracture during milling. PGC consistently showed a modest positive correlation with grain width under both control and stress conditions (Fig. 2b, c), suggesting a relatively close link between these traits.

### Identification of genomic regions regulating HRY through single- and multi-locus GWAS

**Fig. 3.**
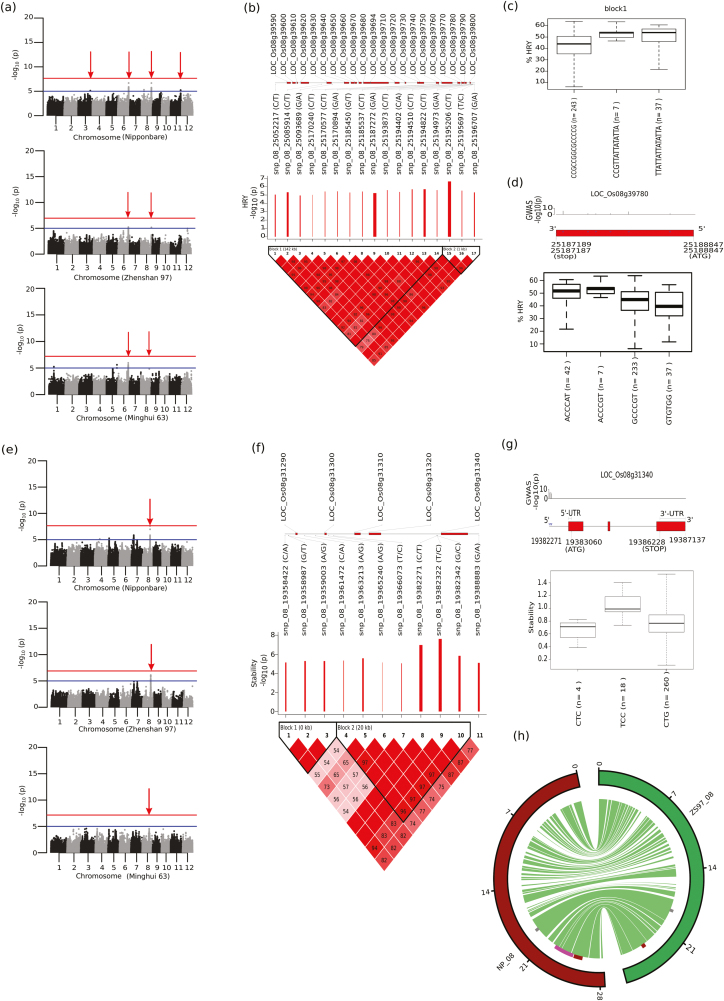
Single-locus GWAS for head rice yield (HRY) in control conditions and for moisture stress stability. (a) Manhattan plots for HRY under control conditions using the reference genomes of *japonica* Nipponbare, and *indica* Zhenshan 97 (ZS97) and Minghui 63 (MH63). Significant genomic loci (threshold –log_10_*P*>5, blue line) for HRY were identified on chromosomes 6, 8, and 11, whilst loci with lower significance were identified on chromosome 3. The loci on chromosomes 6, 8, and 11 (only using the Nipponbare reference) were further confirmed by multi-locus GWAS and are indicated by red arrows. The horizontal red lines represent the genome-wide significance threshold of –log_10_*P* = 7.6. (b) Linkage disequilibrium (LD) plot of the 17 tagged SNPs on chromosome 8. Scaled log_10_(*P*) values are shown and the red bars indicate a relative negative effect. (c) Haplotypes constructed from LD-block 1 are depicted in boxplots with their phenotypic values for HRY. (d) Haplotypes constructed from all significant non-synonymous SNPs (*P*≤0.01) present in the candidate LOC_Os08g039780 showed variations for HRY values. (e) Manhattan plot for moisture stress stability showed a significantly associated genomic region on chromosome 8 (5.7Mb away from the HRY peak) using the Nipponbare reference genome, which was confirmed further in the reference genomes of ZS97 and MH63. (f) LD-plot consisting of two LD-blocks showing 11 tagged SNPs in the 30 Kb region; block-2 showed a high-effect SNP from the upstream region of the gene LOC_Os08g31340, constructing haplotypes possessing significant phenotypic variation for stability, depicted as a box plot (g). (h) Circos diagram representing the structural variation and collinearity in chromosome 8 (physical size shown in Mb), between *japonica* cultivar Nipponbare and *indica* cultivar ZS97. Significantly associated genomic regions for HRY identified in our study (highlighted as red blocks) and in previous studies (pink block) along with the stability-associated genomic region (grey blocks) are indicated; green stripes indicate conserved regions, while white indicates collinearity breaks in the region.

An approach based on a linear mixed model for GWAS was adapted using EMMAX, with both inferred population structure and kinship being used as covariates (Supplementary Fig. S2). Based on the genotyping data of 2 260 030 SNPs obtained from mapping against the *japonica* reference genome, the GWAS was run for all milling quality traits phenotyped in the 320 *indica* lines under controlled drying and moisture stressed conditions from 2015 dry season. We identified association peaks exceeding the threshold (–log_10_*P*>5) for HRY and WHR on chromosome 3 (30.09 Mb), chromosome 6 (27.57–27.62 Mb), chromosome 8 (25.05–25.20 Mb), and chromosome 11 (25.78–25.79) under controlled drying conditions, with a narrow-sense heritability *h*^2^=0.76 (Fig. 3, Supplementary Figs S3, S4). To verify the loci with moderate significance (–log_10_*P*>5), we utilized a multi-locus GWAS analysis and further confirmed that the significant loci for HRY traits on chromosomes 3, 6, 8, and 11 co-located with the single-locus GWAS results (marked as red arrows in Fig 3a and Supplementary S3). Using the genotyping data from the two *indica* reference genomes, we confirmed that the peaks detected in chromosomes 6 and 8 associated with HRY.

Chromosome 6 (27.57–27.62 Mb), including the candidate gene LOC_Os06g45580 that encodes the ubiquitin E3 RING protein with protein degradation activity, possessed a high number of significant SNPs that showed association with HRY (Supplementary Fig. S4A). The targeted association of LOC_Os06g45580 identified several significant indels in the promoter region as well in the 3´-UTR region that explained the higher HRY (50–60 %). The fine-mapped genomic region at 25.05–25.20 Mb on chromosome 8 (HR8.1) significantly associated with HRY and WHR, which spanned 21 candidate genes encompassing 17 tagged SNPs (Table 1) at the interval locus between LOC_Os08g39590 and LOC_Os08g39800 (Fig. 3b), which was further narrowed down by the gene-set analysis to 15 candidate genes (Table 1). The major phenotype variation for HRY was represented by haplotypes from the block 1, consisting of 14 SNPs (Fig. 3b, c) By mining this genetic region, we identified highly significant non-synonymous SNPs (ACCCGT) in LOC_Os08g39780 that exhibited a high mean value of HRY of ~53.7 (Fig. 3d). An additional fine-mapped 9.6-kb region (25.78–25.79 Mb) matching the flanking region of LOC_Os11g42800 was identified on chromosome 11 (HR11.1) and showed significant association with HRY and WHR (Supplementary Fig. S4b). This region was comprised of a single LD-block with a four SNP-based haplotype TTGA that contributed to high mean values of HRY of ~50.71, and was found to be a rare haplotype present in *indica* cultivars. In addition to HRY, the genomic regions that contributed to other milling quality traits under controlled drying conditions were related to PMR and WMR, which were mapped on chromosome 8 (6.10–6.18 Mb) through single-locus GWAS with a relatively high level of significance (–log_10_*P*>7.0) (Supplementary Fig. S3).

**Table 1.** Gene-set analysis using GWAS of candidates identified within the hotspot genomic regions on chromosomes 3, 6, 8, and 11 associated with head rice yield.

Gene ID†	No. of SNPs	SNP positions		Percent head rice			Weight head rice			Annotation
		Start	End	*Z*-Score	*P*-value	Corrected *P*-value^‡^	*Z*-Score	*P*-value	Corrected *P*-value^‡^	
**Chromosome 3**										
LOC_Os03g53990	32	30954587	30959201	2.79	0.0026	0.0784	2.82	0.0024	0.0726	Development unspecified
**Chromosome 6**										
LOC_Os06g45550	16	27572045	27573452	4.10	2.09E^−5^	0.0006	4.10	2.0E^−5^	0.0006	Unclassified
LOC_Os06g45560	91	27583949	27590245	3.60	0.0002	0.0047	3.60	0.0002	0.0046	Unclassified
LOC_Os06g45570	2	27595301	27595529	3.35	0.0004	0.0116	3.35	0.0004	0.0117	Unclassified
LOC_Os06g45580	4	27598795	27599239	3.57	0.0002	0.0051	3.58	0.0002	0.0049	Protein degradation ubiquitin E3 RING
LOC_Os06g45590	5	27601517	27603740	3.84	0.0001	0.0018	3.86	0.0001	0.0017	Plastid branch 3-phosphoglycerate kinase
LOC_Os06g45600	4	27608612	27609181	3.87	0.0001	0.0016	3.89	0.0001	0.0015	Unclassified
LOC_Os06g45610	4	27614080	27614765	3.65	0.0001	0.0039	3.66	0.0001	0.0036	Unclassified
**Chromosome 8**										
LOC_Os08g39590	6	25060904	25062898	2.08	0.0189	0.5484	2.06	0.0199	0.5777	Signalling receptor kinases
LOC_Os08g39600	14	25064684	25066661	1.82	0.0342	0.9929	1.81	0.0354	1.0000	RNA processing
LOC_Os08g39610	5	25068294	25068437	2.40	0.0081	0.2356	2.38	0.0086	0.2501	Unclassified
LOC_Os08g39620	57	25070901	25076423	1.68	0.0466	1.0000	1.67	0.0477	1.0000	Transcription factor
LOC_Os08g39640	53	25102192	25105599	2.33	0.0099	0.2867	2.32	0.0102	0.2953	Cytochrome P450
LOC_Os08g39650	48	25107131	25109344	3.46	0.0003	0.0078	3.45	0.0003	0.0081	Unclassified
LOC_Os08g39660	16	25110579	25111307	2.56	0.0052	0.1513	2.55	0.0053	0.1542	Cytochrome P450
LOC_Os08g39694	94	25121356	25134182	1.80	0.0357	1.0000	1.79	0.0364	1.0000	Cytochrome P450
LOC_Os08g39710	22	25138857	25142157	2.94	0.0016	0.0477	2.93	0.0017	0.0499	Signalling receptor kinases
LOC_Os08g39730	20	25155263	25156844	2.01	0.0220	0.6369	2.03	0.0213	0.6170	Cytochrome P450
LOC_Os08g39760	15	25174628	25179927	3.51	0.0002	0.0065	3.47	0.0003	0.0075	Signalling receptor kinases
LOC_Os08g39770	31	25185266	25186195	2.60	0.0047	0.1362	2.57	0.0051	0.1478	Unclassified
LOC_Os08g39780	36	25187198	25188563	2.35	0.0094	0.2734	2.33	0.0099	0.2882	Unclassified
LOC_Os08g39790	9	25189370	25189553	1.92	0.0273	0.7911	1.90	0.0289	0.8373	Unclassified
LOC_Os08g39800	10	25193197	25193799	1.72	0.0428	1.0000	1.68	0.0463	1.0000	Unclassified
**Chromosome 11**										
LOC_Os11g42800	68	25775818	25778449	1.91	0.0278	0.8059	1.92	0.0272	0.7874	Cell organization

^†^Genes showing *P*≤0.05 were included.

^‡^ *P*-values subjected to Bonferroni correction.

Single-locus GWAS and gene-set analysis were performed to identify genomic regions regulating the stability (HRY ratio of moisture stress and control) along with covariate of HRY values obtained from controlled drying conditions. The HRY stable regions were mapped to a 30.4-kb significant genomic region on chromosome 8 (5.7Mb from the HRY control peak) (Fig. 3e, f, Table 2, Supplementary Table S2). The significant peak was further confirmed by multi-locus GWAS using the two *indica* reference genomes (Fig. 3e). The hotspot genomic region represented by the second LD-block was found to be negatively regulating the stability trait (Fig. 3f). Using TGAS together with gene-set analysis revealed highly significant SNPs located in the upstream region of LOC_Os08g31340 coding for a protein containing heavy metal-associated domain (Fig. 3g, Table 2). The haplotype TCC constructed from these SNPs contributed to higher stability of HRY (~1), meaning no difference between moisture stress versus control. The alternative haplotype CTC was responsible in decreasing HRY under moisture stress relative to the control. Furthermore, the haplotypes identified in the key candidates for influencing HRY on chromosomes 6, 8, and 11 showed consistent behavior across dry and wet seasons (Supplementary Fig. S5).

**Table 2.** Gene-set analysis using GWAS of identified candidates within the hotspot genomic region on chromosomes 8 that regulate stability under moisture stress conditions

Gene ID^†^	SNPs start	SNPs end	No. of SNPs	*Z*-Score	*P*-value	Corrected *P*-value^‡^	Annotations
LOC_Os08g31340	19382952	19387137	10	2.75	0.0030	0.0150	Metal handling binding chelation and storage
LOC_Os08g31320	19371612	19373491	17	3.80	7.34E^−5^	0.0004	Unknown
LOC_Os08g31310	19369347	19370283	9	3.19	0.0007	0.0036	Unknown
LOC_Os08g31300	19364642	19364946	9	2.22	0.0132	0.0661	Unknown

^†^Genes showing *P*≤0.05 were included.

^‡^ *P*-values subjected to Bonferroni correction.

Under stress conditions, GWAS identified a key region on chromosome 1 (37.02–37.73 Mb) that was highly associated with PBR (Supplementary Fig. S3). Another association peak was identified on an 11.14-kb fine-mapped region of chromosome 5 associated with WBR and WMR, located in the upstream region of the *GW5/qSW5* gene (LOC_Os05g09520, encoding a calmodulin-binding motif family protein) that is known to regulate grain width and weight in rice (Weng *et al.*, 2008; Supplementary Fig. S3).

We assessed the level of structural variation that existed in the target regions on chromosomes 3, 6, 8, and 11 between *japonica* (Nipponbare) and *indica* (ZS97) reference genomes, and identified collinearity and break-points in the respective genomes (Fig. 3h, Supplementary Fig. S6). The chromosome 8 hotspot region was located in the conserved regions. Notably, we detected several break-points between ZS97 and Nipponbare, especially within the target hotspot regions for HRY traits mapped on chromosomes 3, 6, and 11 (Supplementary Fig. S6). This suggested the existence of structural variations in the genetic regions that regulate HRY, which reinforced the importance of using subspecies-based reference genome(s) for genotype calling to conduct targeted associations.

### Fine-mapping of the hotspot region on chromosome 5 associated with PGC using GWAS and TGAS

**Fig. 4.**
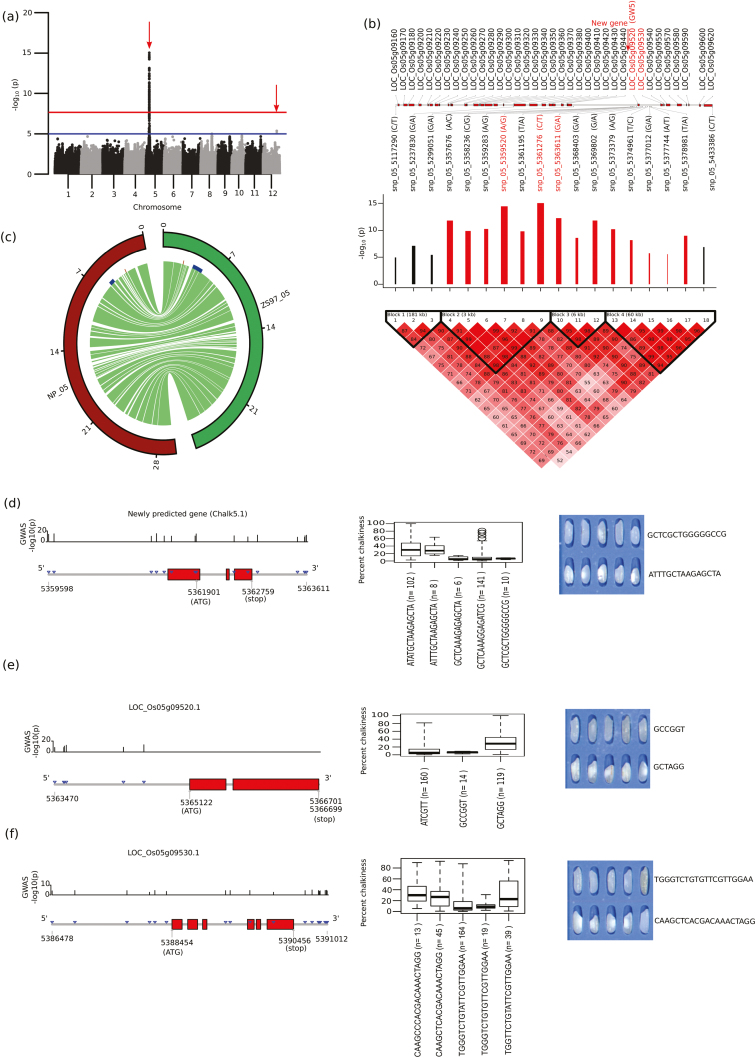
Single-locus GWAS for grain chalkiness and prominent loci identified on chromosome 5 in a diversity panel of 320 *indica* rice accessions using *japonica* reference genomes. (a) Manhattan plots of the GWAS on percentage grain chalkiness (PGC) shows its association with a chromosome 5 hotspot region and chromosome 12 genetic region (red arrow), which was further confirmed by a multi-locus GWAS. The horizontal red and blue lines represent the genome-wide significant threshold –log_10_(*P*) values of 7.6 and 5, respectively. (b) Linkage disequilibrium (LD) plot of the 18 tagged SNPs significantly associated with PGC. A scaled and highly dense LD-based plot within the hotspot genomic region on chromosome 5 is represented. Scaled log_10_(*P*) values are shown, and black bars indicate a relative positive effect whilst red bars indicate a relative negative effect. Genes also detected by TGAS are highlighted in red. (c) Circos diagram showing the collinearity in chromosome 5 between Nipponbare and ZS97, particualry within the hotspot region (marked with blue blocks) where the collinearity break is evident, whereas the previously reported gene *chalk5* that regulates chalkiness was observed more distantly (small red blocks). (d–f) TGAS of the candidates with their gene structures and haplotypes. (d) The newly detected hypothetical gene (named as *chalk5.1*); (e) LOC_Os05g09520; and (f) LOC_Os05g09530. SNPs identified in genic region (marked with blue triangles) with their –log_10_(*P*) values shown above the gene model. The boxplots show constructed haplotypes and related phenotype distribution for PGC; five selected lines from each of two haplotypes that show extreme phenotype are represented in each case.

We conducted GWAS using SNPs acquired based on the alignment of whole-genome sequencing information for 320 diverse *indica* lines with a *japonica* reference genome, and this identified a highly significant (–log_10_*P*=15) fine-mapped genomic region on chromosome 5 mapped between 5.12–5.43 Mb, which further confirmed with the multi-locus GWAS peaks (Fig. 4a–c, Supplementary Fig. S7, red arrows). Within the fine-mapped region, four LD-blocks were defined with 18 tagged SNPs that showed significant association with PGC (Supplementary Table S3). Out of these, the LD-block- 1 region positively influenced PGC phenotype, defined by the CGG haplotype that was derived from three SNPs. The LD- block- 2 and LD- block- 3 regions showed negative association with PGC, with significant beta values, and the LD- block- 4 region showed a weak correlation with PGC. The LD- block- 2 region, which was represented by the haplotype ACGATC (*n*=16), showed higher beta values that explained the lower median PGC value of 6.6 (Fig. 4b, Supplementary Table S4). Using TGAS, the genomic region identified in LD- block- 2 that associated significantly with PGC was narrowed down to three candidate genes. These were LOC_Os05g09520 and LOC_Os05g09530, and a putative novel candidate gene that encodes for a ~122-amino acid protein with unknown function, which we named as *chalk5.1* (Fig. 4d–f). Gene-set analysis identified a highly significant *Z*-score for *chalk5.1*, which was in contrast to the non-significant value (*P*>0.05) for LOC_Os05g09520 (Table 3).TGAS of *chalk5.1* gene identified the haplotype GCTCGCTGGGGGCCG (*n*=10) that covered significant tagged SNPs in the regulatory, exonic, and intronic regions (Fig. 4d). This region (*chalk5.1*) explained the significant phenotypic variance (PV) of 21%. The lines possessing this haplotype had a low-chalkiness phenotype, with a median PGC of 7% (Fig. 4d). These lines were also found to have short grain length due to the presence of the *GW5* gene (LOC_Os05g09520) in the same LD (Fig. 4d). When we mapped the chalkiness hotspot region that showed colinearity on chromosome 5, we observed several break-points between high chalkiness (ZS97) and low chalkiness (Nipponbare) reference genomes within the target hotspot region for PGC on chromosome 5 (Fig. 4c). This indicated the role of existing structural variations underlying the hotspot region in regulating grain chalkiness. Notably, the previously cloned *V-PPase* candidate gene of chalkiness (*chalk5*; Li *et al.*, 2014) was observed at some distance from the target region we identified in the present study (marked red in Fig. 4c).

**Table 3.** Gene-set analysis using GWAS of candidates identified within the hotspot genomic regions on chromosomes 5 associated with percent grain chalkiness.

Gene ID^†^	SNP start	SNP end	No. of SNPs	*Z*-Score	*P*-value^‡^	Corrected	Annotations
**Zhenshan 97 reference**							
ZS05g0093700	4132269	4132855	10	2.49	0.0063	0.2852	Unclassified
ZS05g0093800	4137048	4139938	24	2.39	0.0085	0.3821	Reverse transcriptase
ZS05g0094700	4211463	4222967	3	3.28	0.0005	0.0236	Phosphoinositide 3-kinase
Newly predicted gene (*Chalk 5.1*)	4599233	4600542	8	4.87	5.68E^−7^	2.56E^−5^	Unclassified
ZS05g0110100	4624872	4627897	1	2.68	0.0036	0.1639	Aspartic peptidase
ZS05g0110200	4629326	4630336	1	3.84	0.0001	0.0028	Unclassified
ZS05g0110300	4632404	4636282	1	2.92	0.0017	0.0785	Der1-like protein
ZS05g0110700	4657254	4678577	40	2.16	0.0155	0.6953	Armadillo-type fold
ZS05g0110900	4682839	4685046	2	2.45	0.0072	0.3244	Ubiquinone biosynthesis protein
ZS05g0111000	4688129	4693152	11	3.05	0.0012	0.0521	Acid phosphatase
ZS05g0111100	4698217	4699046	4	2.92	0.0017	0.0781	Acid phosphatase
ZS05g0111200	4705604	4707744	5	2.12	0.0172	0.7729	Acid phosphatase
ZS05g0112300	4793937	4795814	8	2.65	0.0040	0.1814	Acid phosphatase
ZS05g0112500	4847621	4848943	2	1.71	0.0439	1.0000	FAR1-DNA binding domain
ZS05g0112900	4878450	4879311	1	2.17	0.0150	0.6739	Ulp1-protease-like protein
ZS05g0115100	5039853	5040292	1	1.82	0.0344	1.0000	Unclassified
ZS05g0115200	5057235	5058552	2	3.01	0.0013	0.0593	SANT/Myb domain
**Minghui 63 reference**							
MH05g0089300	5011370	5012335	1	4.67	1.53E^−6^	6.14E^−6^	Unclassified
MH05g0091000	5069302	5070093	2	1.91	0.0280	0.1120	DUF protein
**Nipponbare reference**							
LOC_Os05g09200	5140748	5143285	1	3.97	3.57E^−5^	0.0008	Unclassified
LOC_Os05g09220	5152077	5154824	2	2.53	0.0057	0.1262	Unclassified
LOC_Os05g09230	5157489	5160847	4	2.19	0.0142	0.3129	Unclassified
Newly predicted gene (*Chalk 5.1*)	5361454	5362761	8	6.41	7.23E^−11^	1.59E^−9^	Unclassified
LOC_Os05g09530	5388454	5390458	4	3.80	7.38E^−5^	0.0016	Protein degradation aspartate protease
LOC_Os05g09540	5391521	5392195	5	4.37	6.10E^−6^	0.0001	Unclassified
LOC_Os05g09550	5394336	5398141	8	3.55	0.0002	0.0042	Protein degradation
LOC_Os05g09590	5417150	5417753	15	3.72	0.0001	0.0022	Unclassified
LOC_Os05g09600	5419267	5420744	1	4.15	1.67E^−5^	0.0004	Unclassified
LOC_Os05g09620	5424306	5433090	26	3.51	0.0002	0.0049	Unclassified

^†^Genes showing the significance of *P*≤0.05 were included.

^‡^ *P*-values are corrected as per Bonferroni corrections.

Using genotyping data derived from the ZS97 reference genome (Zhang *et al.*, 2016), GWAS also identified significant association on chromosome 5 with a region of ~0.9 Mb that harboured four major LD-blocks (Supplementary Fig. S7, S8, Supplementary Table S5). The SNPs with strong effects in conferring the low-chalkiness phenotype (Supplementary Fig. S8a, b) found within the candidate *chalk5.1* gene (unknown function) were present in a separate LD-block (Supplementary Fig. S8a) while *GW5*/*qSW5* (LOC_Os05g09520) that regulates grain width is in another LD-block (Shomura *et al.*, 2008; Weng *et al.*, 2008). The reference allele of the topmost SNP (snp_05_5361276) detected in the 5´-UTR of *chalk5.1* matched with the binding site for tri-helix protein (Supplementary Fig S9). Similarly, other strongly associated SNPs identified in the upstream region of *chalk5.1* were mapped within the binding site of trans-regulatory elements including AP2, C2H2, MYB, TIFY-transcription factor, and TBP (TATA-binding protein) (Supplementary Fig. S9).

**Fig. 5.**
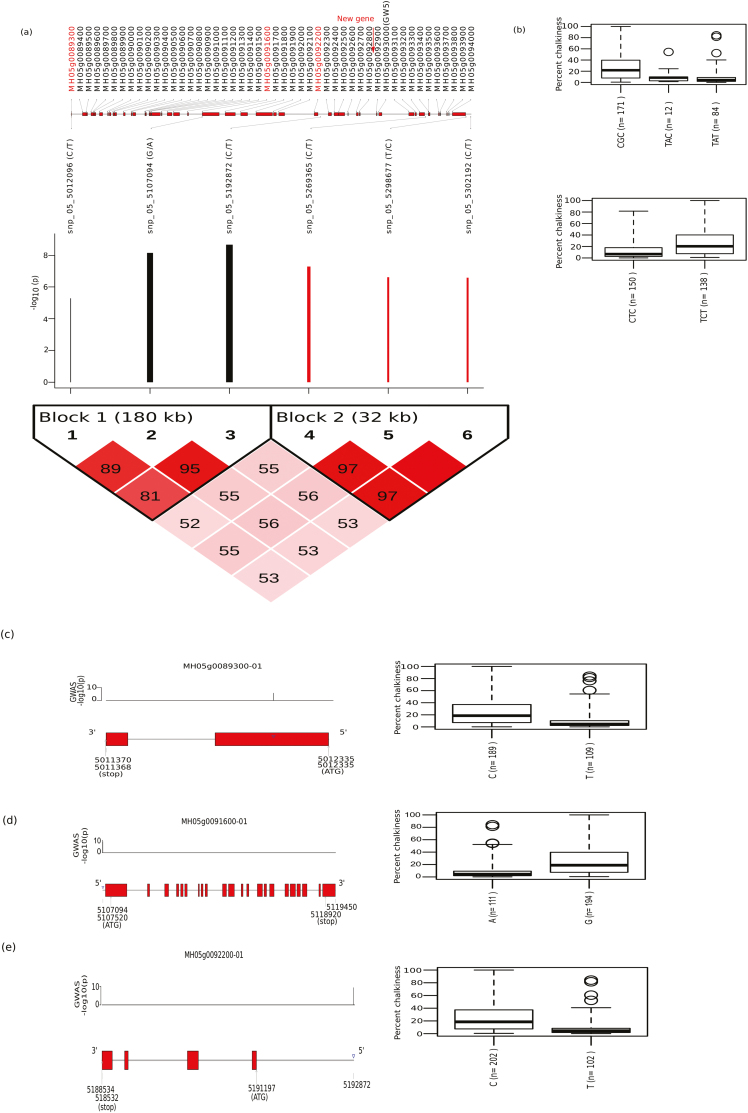
Single-locus GWAS for percentage grain chalkiness (PGC) identified a highly associated chromosome 5 hotspot region using Minghui63 (*indica*) as a reference genome. (a) Linkage disequilibrium (LD) plot of the six tagged SNPs significantly associated with PGC. A scaled and highly dense LD-based plot within the hotspot genomic region is represented and the position of newly predicted gene is highlighted. Scaled log_10_(*P*) values are shown for the six SNPs, and black bars indicate a relative positive effect whilst red bars indicate a relative negative effect. (b) Haplotypes constructed within two LD blocks and their respective phenotypic values for PGC are represented as boxplots. (c–e) TGAS of selected genes: (c) MH05g0089300, (d) MH05g0091600, (e) MH05g0092200. These genes are highlighted in red in (a) based on their high effects with respective to PGC values. The boxplots show constructed haplotypes and related phenotype distribution for PGC.

Using MH63 reference genome, a highly significant region associated with PGC was observed at a narrow interval of ~290 kb (Fig. 5a, Supplementary Table S6). The region was divided into two LD-blocks of 181 kb and 33 kb, separated by a region of 76 kb. The LD-block 1 containing three SNPs (CGC) correlated positively with PGC, whereas the SNPs located in LD-block 2 (matching the *GW5* region) exerted a negative effect on chalkiness with a lower beta value. This might be attributable to the presence of few SNPs in the target region (Supplementary Fig. S10). TGAS analysis resulted in the identification of three candidate genes (MH05g0089300, MH05g0091600, MH05g0092200), which were confirmed through gene-set analysis (Fig. 5, Table 3). The significant SNP (snp_05_5107094, G/A) identified in LD-block 1 matched the candidate gene MH05g0091600 (Supplementary Table S6). These results suggested that possible genomic variations (insertion/deletion) exist in the identified targeted region.

We additionally evaluated the different *indica* germplasm panels for chalkiness in field trials conducted across multiple seasons and years and consistently identified the same peak region as being highly associated with grain chalkiness (data not shown). Moreover, this region was found to be unique as it did not match any of the cloned chalk genes/mutants that have previously been reported (Supplementary Table S7).

### HRY and chalkiness genetic regions for improving milling and appearance qualities

**Fig. 6.**
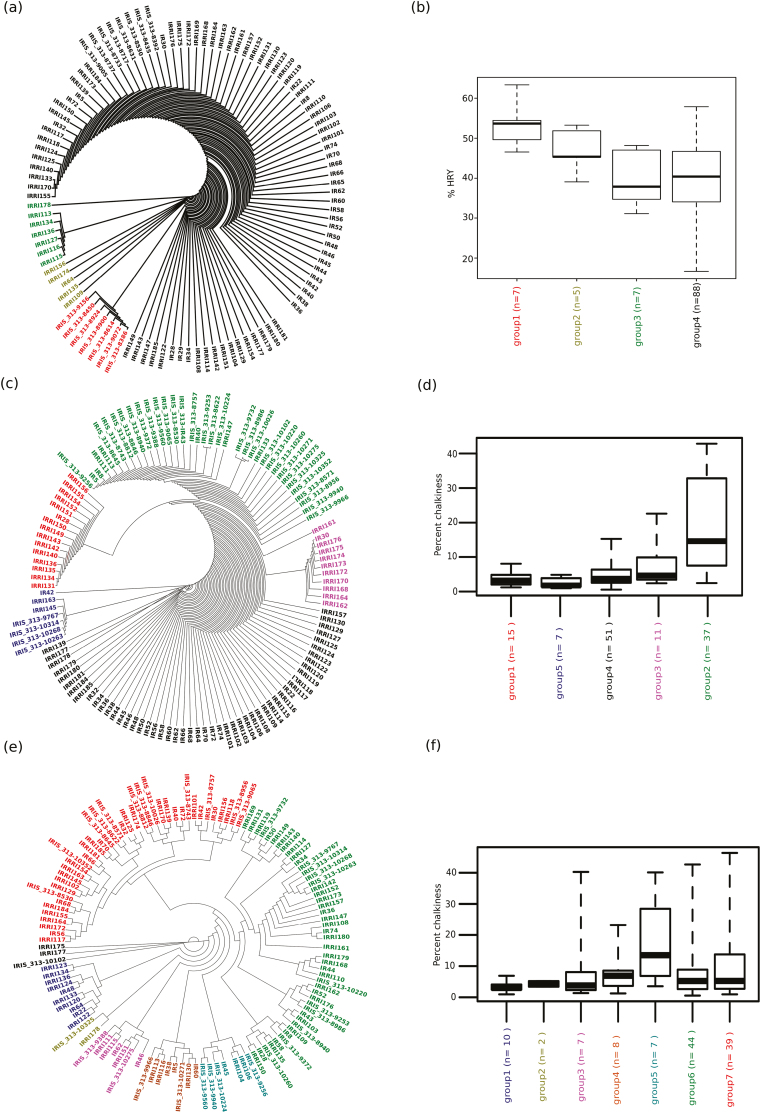
Haplotype distribution for head rice yield (HRY) and chalkiness in IRRI breeding lines together with selected diversity lines. An unrooted dendrogram (a) and a boxplot (b) depict the allelic variations for key haplotypes of the chromosome 8 hotspot region that governs HRY traits. The distribution indicated four different groups. Group-1 (red) represents high HRY cultivars (median 53.7%) as identified in the core collection with the presence of high-HRY haplotypes. Group-2 (yellow/green) had the second highest HRY values (median 45.4%) and included the broadly adapted mega-variety IR64 and other advanced breeding lines. Group-3 (green) mainly included recently released breeding lines with relatively lower and variable HRY values (median 37.84). Group 4 (black) included a wide range of HRY values (median 40.41). (c) Unrooted dendrogram and (d) boxplot based on allelic variations within the novel putative candidate gene *chalk5.1*, where group 5 (blue) represents the lowest chalkiness value (median 2.8) with lowest variance in three IRRI breeding lines and four accessions, while group 1 (red) showed a low chalk value (median 3.2) and represented only IRRI breeding lines. (e) Unrooted dendrogram and (f) boxplot showing allelic variation in the previously characterized *chalk5* gene.

Out of 106 breeding lines, we resequenced 92 lines at a depth of ~30× coverage to identify the distribution of beneficial alleles for HRY and chalkiness. These lines were developed at IRRI and released between 1966–2015. To validate the significance of the allelic diversity that existed in the hotspot region of chromosome 8 (25.05–25.20 Mb) that resulted in high HRY in the selected *indica* panel, we examined existing variations within the IRRI breeding lines for these beneficial haplotypes. For HRY four main cluster groups were identified (Fig. 6a, b). Interestingly, accessions with the high HRY haplotype (group 1) identified in the rare *indica* accessions with a median HRY of 53.7% were clustered separately from the rest of the lines. The adjacent cluster group 2 encompassed the IRRI mega-varieties IR64, IRRI109, IRRI135, IRRI156, and IRRI174 that possessed alternative allelic combinations that resulted in a median HRY value of 45.37%. IR64 is a highly adapted mega-variety that harbours superior alleles for HRY, and it was released in the 1980s and accounts for the highest acreage in South and Southeast Asia (Mackill and Khush, 2018). Group 3 clustered around a newly developed IRRI breeding line with a poor median HRY value of 37.84%, and group 4 was represented by a range of breeding lines with a variable range of HRY values (Fig. 6a).

A dendrogram constructed by considering the entire hotspot region (using 18 tagged SNPs) of chromosome 5 (5.12–5.43 Mb) that influenced PGC classified the breeding lines into four groups (Supplementary Fig. S11). A phylogentic grouping of IRRI breeding lines based on 15 significant SNPs identified in the new candidate gene *chalk5.1* also identified five groups (Fig. 6c, d). Notably, allelic variation in group 1 potentially demonstrated a narrower range of the low-chalkiness phenotype (median 3.2%) and represented many modern IRRI breeding lines, followed by group 5 (median 2.8%) that represented diversity lines originating from low-chalkiness material (Fig. 6c, d). Beneficial alleles present in groups 1 and 5 in the IRRI-breeding lines conferred to reduced chalkiness. Many breeding lines represented in groups 3 and 4 showed a relatively broad range of chalkiness (Fig. 6c, d). Group 2 included the high-chalkiness IR5, IR8, and IR40 lines that were released during an early phase of IRRI’s breeding program, and they possess the haplotype that explains the high median PGC value of 13.9%. This high-chalkiness haplotype was also found in few breeding lines that were released more recently such as IRRI111, IRRI113, and IRRI147. The PV explained by *chalk5.1* for the chalkiness phenotype in the breeding material was 11.42%, in contrast to 16.72% for *chalk5*. This suggests a relative lower representation of the *chalk5.1* allele than *chalk5* across the breeding lines (Fig. 6e, f). Intriguingly, the combined PV of both genic regions was assessed as 26.12%, which suggests the synergistic contribution of both candidate genes.

## Discussion

High head rice yield (HRY) and low chalkiness are key traits that determine the milling and appearance qualities of rice, and hence they can significantly affect economic returns to farmer and millers. Current trends of limited genetic gains in yield *per se* combined with lack of progress in other important economic traits related to HRY have made it difficult to replace the widely adopted rice mega-varieties (Laborte *et al.*, 2015; Sreenivasulu *et al.*, 2015). Several trends assessed from market surveys and a lack of genetic diversity for HRY in high-yielding breeding lines collectively suggest that diverse genetic resources need to be exploited in order to improve milling quality. Rice industries around the world have observed that climate change has contributed to increasing temperatures during the dry season, which is linked to higher incidence of breakage of rice grains during milling (Custodio *et al.*, 2016; Zohoun *et al.*, 2018). In some countries premium slender (and fragrant) rice is typically grown in the dry season due to higher yields and lower production risks associated with diseases, and in accordance with this our market survey data indicated a higher importance of HRY in the dry season in countries such as the Philippines, Bangladesh, and India (Fig. 1a). In Eastern India, parboiling has been widely used to maximize head rice recovery (Custodio *et al.*, 2016). However, rising fuel costs for cooking have decreased this practice by shifting preferences from parboiled to non-parboiled rice. In addition, increasing demand for rice with slender grains in Asian (Custodio *et al.*, 2016) and African regions (Demont *et al.*, 2017) will introduce trade-offs between breeding for slenderness and head rice recovery, as these are generally considered as antagonistic traits (i.e. slender rice breaks more easily during milling), although some key mega-varieties such as IR64 typically feature both slender grains and high HRY (IRRI, 1986; Mackill and Khush, 2018) due to superior HRY alleles. In regular breeding programs, HRY has not been subjected to intensive selection (Zhao and Fitzgerald, 2013; Zhou *et al.*, 2015), due to the availability of only a limited amount of seed material during initial filial generations and due to lack of a robust high-throughput phenotyping method. Conversely, using chalkiness as a proxy trait for improving milling quality has resulted in improving the degree of chalkiness in IRRI breeding programs (mean of 4%) (Fig. 1), but has resulted in only limited progress for improving HRY, the mean for which remains at 45% among the most recently released breeding varieties. Using chalkiness as a proxy measure for HRY has been a common assumption that has pervaded rice breeding programs for decades, clearly it is not necessarily effective. This emphasises the need for identifying the genetics of HRY.

Since HRY is known to be a low-heritable trait that is influenced primarily by moisture stress in the grain and thus by post-harvest practices (Siebenmorgen *et al.*, 2007, 2013; Nelson *et al.*, 2011), we used controlled drying conditions to improve HRY (Cnossen *et al.*, 2003). HRY data obtained from using controlled drying conditions for 320 resequenced diverse *indica* lines combined with ultra-dense SNP genotyping allowed us to identify fine-mapped genetic regions from chromosome 6 and 8 with relatively high heritability (Fig. 3, Supplementary Fig. S4). In contrast, using traditional bi-parental populations has resulted in limited success in identifying QTLs for HRY in *japonica* rice due to low heritability and restricted recombination events (Nelson *et al.*, 2012; Pinson *et al.*, 2013; Ren *et al.*, 2016; Li *et al.*, 2018). In our present study, the ultra-high SNP density was up to 3-fold higher than that of recently published reports of GWAS-based studies in rice (Crowell *et al.*, 2016; McCouch *et al.*, 2016; Wang *et al.*, 2016b). This enabled high-resolution mining of trait-specific allelic variants for higher HRY on chromosome 8 (25.05–25.20 Mb) under controlled drying (Fig. 3). An additional genomic region on chromosome 8 for stability was identified more distant from the controlled HRY region, which was not detected in a previous study (Qiu *et al.*, 2015) and might have been due to limited marker numbers and smaller population size. Fine-mapping these genomic regions is more appropriate in *indica*, where rapid LD decays occur compared to tropical and temperate *japonica* (Huang *et al.*, 2011, 2013).

Resequencing of popular IRRI varieties and studying target regions of HRY allowed us to inspect the allelic variations in the hotspot region for HRY on chromosome 8. Our study established that beneficial haplotypes responsible for high HRY that were present in only a few of the ancestral accessions were typically clustered together in group 1 (Fig. 6). It is highly likely that these underlying beneficial alleles have not been selected during breeding selection cycles. Many breeding lines in group 3 and 4 were characterized by inferior alleles resulting in poor HRY. As a result, no significant genetic improvements were made for HRY in the post-Green Revolution breeding programs. IR64 was found in group 2 with moderate HRY, together with the other modern varieties IRRI109, IRRI135, IRRI156, and IRRI174. IR64 is a popular mega-variety that is widely planted in India, Indonesia, Pakistan, the Philippines, and Vietnam, possibly due to its reasonable HRY coupled with a softer texture, which results in a wide preference by farmers and millers (Khush and Virk, 2005; Mackill and Khush, 2018).

Farmers generally sell their harvest to millers, who prefer rice grains without chalkiness as it is perceived to lessen the predisposition to grain breakage during dehulling and polishing (Del Rosario *et al.*, 1968; Lisle *et al.*, 2000; Champagne, 2004; Wang *et al.*, 2007; Cooper *et al.*, 2008). Although previous studies have indicated out the inverse relationship between PGC and HRY (Buggenhout *et al.*, 2013; Zhao and Fitzgerald, 2013), in our present study using most of the IRRI rice varieties and a diversity rice panel we found lower correlations (Fig. 2b, c). Grain chalkiness with high heritability is judged as a crucial determinant for grading the export quality of rice (Wan *et al.*, 2005; Zhao *et al.*, 2016). Using GWAS, we detected a novel and highly significant association signal from a 0.31-Mb hotspot region of chromosome 5 that was associated with PGC (Fig. 4a, b) and observed it consistently across multiple seasons and replications in field trials in *indica* lines. This ruled out a major influence of environmental factors on the hotspot region. The region was located at a significant distance (1.78 Mb) from the previously cloned gene *chalk5 V-PPase* that encodes for inorganic pyrophosphate (PPi) H^+^-translocation activity (Li *et al.*, 2014). Moreover, the hotspot region that regulates chalkiness detected in our present study represent the novel *chalk 5.1* gene, was found distinct from previously reported regions (Peng *et al.*, 2014; Zhao *et al.*, 2015, 2016; Chen *et al.*, 2016; Gao *et al.*, 2016; Yun *et al.*, 2016; Zhu *et al.*, 2018).

TGAS in combination with GWAS allowed us to narrow down the hotspot region to a locus encompassing three candidate genes (Fig. 4e), which included a known gene for grain width, *GW5*. In this region, deletion of 1212 bp from the ~5-kb upstream of the *GW5* (LOC_Os05g9520) has been reported to contribute to higher grain width in *japonica* cultivars (Shomura *et al.*, 2008; Liu *et al.*, 2017). In addition, *GW5* was also been considered as a putative candidate for reducing PGC (Qiu *et al.*, 2015; Yun *et al.*, 2016). Furthermore, by using the reference genome of the high-chalkiness *indica* line ZS97 (Zhang *et al.*, 2016) for calling SNPs, our GWAS results identified a new putative candidate gene *chalk5.1* for regulating PGC situated ~3 kb upstream of *GW5* (Fig. 4d). The lowest PGC value, confirmed by the haplotype of the *chalk5.1* gene (Fig. 4d), suggested its importance in lowering chalkiness. We found lower PV values in breeding lines for *chalk5.1* (11.4%) compared to *chalk5 V-PPase* (16.7%), which can be attributed to the lower frequency of representation of *chalk5.1* alleles in the breeding material. Together, the two chalkiness genes identified on chromosome 5 explain 26.12% of the PV, indicating their likely potential benefits in future breeding initiatives to reduce chalkiness.

In summary, the fine-mapped haplotypes of chromosome 5 identified for reducing chalk have been successfully explored in the present IRRI breeding programs. In contrast, the novel haplotypes identified for enhanced HRY from chromosome 8 in the diversity panel were not found in the 92 IRRI breeding lines. To ensure future economic benefits for farmers, superior alleles contributing for high HRY from chromosome 8 need to be recombined with the *chalk 5.1* gene in the rice breeding programs to improve milling and appearance quality attributes for long-slender *indica* varieties.

## Supplementary data

Supplementary data are available at *JXB* online.

Table S1. List of accessions used in the study together with the haplotypes for HRY that were identified in the hotspot regions of chromosomes 8 and 11.

Table S2. List of haplotypes identified for HRY stability within the candidate region on chromosome 8 in the accessions used in this study.

Table S3. Single-locus GWAS for SNPs detected against the Nipponbare reference genome with corresponding orthologous IDs in ZS97 and MH63.

Table S4. List of haplotypes identified for lowering chalkiness in hotspot region on chromosome 5 in the accessions used in this study.

Table S5. Single-locus GWAS for SNPs detected against the ZS97 reference genome with corresponding orthologous IDs in MH63 and Nipponbare.

Table S6. Single-locus GWAS for SNPs detected against the MH63 reference genome with corresponding orthologous IDs in ZS97 and Nipponbare.

Table S7. Genes/QTLs previously characterized in mutants as influencing grain chalkiness.

Fig. S1. Phenotypic variation for milling quality traits and chalkiness in 320 resequenced *indica* lines.

Fig. S2. Principal component analysis for the 320 resequenced *indica* lines.

Fig. S3. Manhattan plots generated by GWAS for milling quality traits within the *indica* germplasm.

Fig. S4. Single-locus GWAS for HRY showing the association of the genomic regions of chromosomes 6 and 11.

Fig. S5. Variation in the phenotypic values of HRY and chalkiness observed for different haplotypes across dry and wet seasons.

Fig. S6. Comprehensive representation of structural variation and collinearity between the *japonica* cultivar Nipponbare and the *indica* cultivar ZS97.

Fig. S7. Single-locus GWAS for grain chalkiness showing the main candidate loci on chromosomes 5 using the *indica* reference genomes ZS97 and MH63.

Fig. S8. Single-locus GWAS for PGC showing the significant association in the chromosome 5 hotspot region using ZS97 as the reference genome.

Fig. S9. Nucleotide sequence alignment in the main chromosome 5 hotspot region within cultivars with extreme chalkiness phenotypes.

Fig. S10. SNPs in the genomic sequence of the chromosome 5 hotspot region that regulate PGC in the three reference genomes, Nipponbare, ZS97, and MH63.

Fig. S11. Allelic variation in the chromosome 5 hotspot region and allele distribution in IRRI breeding lines and selected core-collection cultivars.

erz256_suppl_Supplementary_Figures_S1-S11

erz256_suppl_Supplementary_Tables_S1-S7

## Author contributions

GM and RA conducted the GWAS, TGAS, and haplotype analyses; RA supervised the planting, harvesting, and drying of the rice diversity panel and IRRI varieties; MD conducted and interpreted the farmer survey; VB and LM optimized the methods for chalkiness and HRY assays, and SB analysed the data; NS, AG, and MKM conceptualized the work and NS supervised the conduct of all experiments and bioinformatics analyses; NS wrote the manuscript with contributions from VB and SB; all authors read and contributed to the revision of manuscript.

## References

[CIT0001] Alexandrov N, Tai S, Wang W, et al 2015 SNP-Seek database of SNPs derived from 3000 rice genomes. Nucleic Acids Research 43, D1023–D1027. 25429973 10.1093/nar/gku1039 PMC4383887

[CIT0002] Altschul SF, Gish W, Miller W, Myers EW, Lipman DJ 1990 Basic local alignment search tool. Journal of Molecular Biology 215, 403–410. 2231712 10.1016/S0022-2836(05)80360-2

[CIT0003] Barrett JC, Fry B, Maller J, Daly MJ 2005 Haploview: analysis and visualization of LD and haplotype maps. Bioinformatics 21, 263–265. 15297300 10.1093/bioinformatics/bth457

[CIT0004] Buggenhout J, Brijs K, Celus I, Delcour J 2013 The breakage susceptibility of raw and parboiled rice: a review. Journal of Food Engineering 117, 304–315.

[CIT0005] Butardo VM Jr, Anacleto R, Parween S, Samson I, de Guzman K, Alhambra CM, Misra G, Sreenivasulu N 2017 Systems genetics identifies a novel regulatory domain of amylose synthesis. Plant Physiology 173, 887–906. 27881726 10.1104/pp.16.01248 PMC5210722

[CIT0006] Butardo VM Jr, Sreenivasulu N 2019 Improving head rice yield and milling quality: state-of-the-art and future prospects. Methods in Molecular Biology 1892, 1–18. 30397797 10.1007/978-1-4939-8914-0_1

[CIT0007] Champagne ET(ed). 2004 Rice: chemistry and technology. St Paul, MN: American Association of Cereal Chemists.

[CIT0008] Chen L, Gao W, Chen S, Wang L, Zou J, Liu Y, Wang H, Chen Z, Guo T 2016 High-resolution QTL mapping for grain appearance traits and co-localization of chalkiness-associated differentially expressed candidate genes in rice. Rice 9, 48. 27659284 10.1186/s12284-016-0121-6 PMC5033801

[CIT0009] Cnossen AG, Jimenez MJ, Siebenmorgen TJ 2003 Rice fissuring response to high drying and tempering temperatures. Journal of Food Engineering 59, 61–69.

[CIT0010] Cooper NTW, Siebenmorgen TJ, Counce PA 2008 Effects of nighttime temperature during kernel development on rice physicochemical properties. Cereal Chemistry 85, 276–282.

[CIT0011] Crowell S, Korniliev P, Falcão A, Ismail A, Gregorio G, Mezey J, McCouch S 2016 Genome-wide association and high-resolution phenotyping link *Oryza sativa* panicle traits to numerous trait-specific QTL clusters. Nature Communications 7, 10527. 10.1038/ncomms10527 PMC4742901 26841834

[CIT0012] Custodio MC, Demont M, Laborte A, Ynion J 2016 Improving food security in Asia through consumer-focused rice breeding. Global Food Security 9, 19–28.

[CIT0013] de Leeuw CA, Mooij JM, Heskes T, Posthuma D 2015 MAGMA: generalized gene-set analysis of GWAS data. PLoS Computational Biology 11, e1004219. 25885710 10.1371/journal.pcbi.1004219 PMC4401657

[CIT0014] Del Rosario AR, Briones VP, Vidal AJ, Juliano BO 1968 Composition and endosperm structure of developing and mature rice kernel. Cereal Chemistry 45, 225–235.

[CIT0015] Demont M, Fiamohe R, Kinkpé AT 2017 Comparative advantage in demand and the development of rice value chains in West Africa. World Development 96, 578–590.

[CIT0016] Fujita N, Yoshida M, Kondo T, et al 2007 Characterization of SSIIIa-deficient mutants of rice: the function of SSIIIa and pleiotropic effects by SSIIIa deficiency in the rice endosperm. Plant Physiology 144, 2009–2023. 17586688 10.1104/pp.107.102533 PMC1949899

[CIT0017] Gao Y, Liu C, Li Y, et al 2016 QTL analysis for chalkiness of rice and fine mapping of a candidate gene for qACE9. Rice 9, 41. 27549111 10.1186/s12284-016-0114-5 PMC4993740

[CIT0018] Huang X, Lu T, Han B 2013 Resequencing rice genomes: an emerging new era of rice genomics. Trends in Genetics 29, 225–232. 23295340 10.1016/j.tig.2012.12.001

[CIT0019] Huang X, Zhao Y, Wei X, et al 2011 Genome-wide association study of flowering time and grain yield traits in a worldwide collection of rice germplasm. Nature Genetics 44, 32–39. 22138690 10.1038/ng.1018

[CIT0020] IRRI 1986 Annual report for 1985. Los Baños: International Rice Research Institute.

[CIT0021] Kang HG, Park S, Matsuoka M, An G 2005 White-core endosperm floury endosperm-4 in rice is generated by knockout mutations in the C-type pyruvate orthophosphate dikinase gene (*OsPPDKB*). The Plant Journal 42, 901–911. 15941402 10.1111/j.1365-313X.2005.02423.x

[CIT0022] Kang HM, Sul JH, Service SK, Zaitlen NA, Kong SY, Freimer NB, Sabatti C, Eskin E 2010 Variance component model to account for sample structure in genome-wide association studies. Nature Genetics 42, 348–354. 20208533 10.1038/ng.548 PMC3092069

[CIT0023] Khush GS, Virk PS 2005 IR varieties and their impact. Los Baños: International Rice Research Institute.

[CIT0024] Krzywinski M, Schein J, Birol I, Connors J, Gascoyne R, Horsman D, Jones SJ, Marra MA 2009 Circos: an information aesthetic for comparative genomics. Genome Research 19, 1639–1645. 19541911 10.1101/gr.092759.109 PMC2752132

[CIT0025] Laborte AG, Paguirigan NC, Moya PF, Nelson A, Sparks AH, Gregorio GB 2015 Farmers’ preference for rice traits: insights from farm surveys in Central Luzon, Philippines, 1966-2012. PLoS ONE 10, e0136562. 26317505 10.1371/journal.pone.0136562 PMC4552743

[CIT0026] Lanning SB, Siebenmorgen TJ, Counce PA, Ambardekara AA, Mauromoustakos A 2011 Extreme nighttime air temperatures in 2010 impact rice chalkiness and milling quality. Field Crops Research 124, 132–136.

[CIT0027] Li X, Wu L, Wang J, Sun J, Xia X, Geng X, Wang X, Xu Z, Xu Q 2018 Genome sequencing of rice subspecies and genetic analysis of recombinant lines reveals regional yield- and quality-associated loci. BMC Biology 16, 102. 30227868 10.1186/s12915-018-0572-x PMC6145349

[CIT0028] Li Y, Fan C, Xing Y, et al 2014 *Chalk5* encodes a vacuolar H^+^-translocating pyrophosphatase influencing grain chalkiness in rice. Nature Genetics 46, 398–404. 24633159 10.1038/ng.2923

[CIT0029] Lisle AJ, Martin M, Fitzgerald MA 2000 Chalky and translucent rice grains differ in starch composition and structure and cooking properties. Cereal Chemistry 77, 627–632.

[CIT0030] Liu J, Chen J, Zheng X, et al 2017 GW5 acts in the brassinosteroid signalling pathway to regulate grain width and weight in rice. Nature Plants 3, 17043. 28394310 10.1038/nplants.2017.43

[CIT0031] Mackill DJ, Khush GS 2018 IR64: a high-quality and high-yielding mega variety. Rice 11, 18. 29629479 10.1186/s12284-018-0208-3 PMC5890005

[CIT0032] Maligalig RL 2018 Eliciting farmer preferences for rice varietal trait improvements using an experimental methodology based on investment games. Adelaide, Australia: University of Adelaide.

[CIT0033] McCouch SR, Wright MH, Tung CW, et al 2016 Open access resources for genome-wide association mapping in rice. Nature Communications 7, 10532. 10.1038/ncomms10532 PMC4742900 26842267

[CIT0034] Misra G, Badoni S, Anacleto R, Graner A, Alexandrov N, Sreenivasulu N 2017 Whole genome sequencing-based association study to unravel genetic architecture of cooked grain width and length traits in rice. Scientific Reports 7, 12478. 28963534 10.1038/s41598-017-12778-6 PMC5622062

[CIT0035] Nelson J, Jodari F, Roughton A, McKenzie K, McClung A, Fjellstrom R, Scheffler B 2012 QTL mapping for milling quality in elite western US rice germplasm. Crop Science 52, 242–252.

[CIT0036] Nelson JC, McClung AM, Fjellstrom RG, Moldenhauer KA, Boza E, Jodari F, Oard JH, Linscombe S, Scheffler BE, Yeater KM 2011 Mapping QTL main and interaction influences on milling quality in elite US rice germplasm. Theoretical and Applied Genetics 122, 291–309. 20857082 10.1007/s00122-010-1445-z

[CIT0037] Peng B, Wang L, Fan C, Jiang G, Luo L, Li Y, He Y 2014 Comparative mapping of chalkiness components in rice using five populations across two environments. BMC Genetics 15, 49. 24766995 10.1186/1471-2156-15-49 PMC4021085

[CIT0038] Peng S, Huang J, Sheehy J, Laza R, Visperas R, Zhong X, Centeno G, Khush G, Cassman K 2004 Rice yields decline with higher night temperature from global warming. Proceedings of the National Academy of Sciences, USA 101, 9971–9975. 10.1073/pnas.0403720101 PMC454199 15226500

[CIT0039] Pinson SR, Jia Y, Gibbons JW 2013 Three quantitative trait loci conferring resistance to kernel fissuring in rice identified by selective genotyping in two tropical japonica populations. Crop Science 53, 2434–2443.

[CIT0040] Qiu X, Pang Y, Yuan Z, Xing D, Xu J, Dingkuhn M, Li Z, Ye G 2015 Genome-wide association study of grain appearance and milling quality in a worldwide collection of *indica* rice germplasm. PLoS ONE 10, e0145577. 26714258 10.1371/journal.pone.0145577 PMC4694703

[CIT0041] Ray DK, Ramankutty N, Mueller ND, West PC, Foley JA 2012 Recent patterns of crop yield growth and stagnation. Nature Communications 3, 1293. 10.1038/ncomms2296 23250423

[CIT0042] Ren D, Rao Y, Huang L, et al 2016 Fine mapping identifies a new QTL for brown rice rate in rice (*Oryza sativa* L.). Rice 9, 4. 26847792 10.1186/s12284-016-0076-7 PMC4742455

[CIT0043] Shomura A, Izawa T, Ebana K, Ebitani T, Kanegae H, Konishi S, Yano M 2008 Deletion in a gene associated with grain size increased yields during rice domestication. Nature Genetics 40, 1023–1028. 18604208 10.1038/ng.169

[CIT0044] Siebenmorgen TJ, Bautista R, Counce P 2007 Optimal harvest moisture contents for maximizing milling quality of long- and medium-grain rice cultivars. Applied Engineering in Agriculture 23, 517–527.

[CIT0045] Siebenmorgen TJ, Grigg BC, Lanning SB 2013 Impacts of preharvest factors during kernel development on rice quality and functionality. Annual Review of Food Science and Technology 4, 101–115. 10.1146/annurev-food-030212-182644 23464570

[CIT0046] Sreenivasulu N, Butardo VM Jr, Misra G, Cuevas RP, Anacleto R, Kavi Kishor PB 2015 Designing climate-resilient rice with ideal grain quality suited for high-temperature stress. Journal of Experimental Botany 66, 1737–1748. 25662847 10.1093/jxb/eru544 PMC4669556

[CIT0047] Tamba CL, Ni YL, Zhang YM 2017 Iterative sure independence screening EM-Bayesian LASSO algorithm for multi-locus genome-wide association studies. PLoS Computational Biology 13, e1005357. 28141824 10.1371/journal.pcbi.1005357 PMC5308866

[CIT0048] The 3000 Rice Genomes Project 2014 The 3,000 rice genomes project. GigaScience 3, 7. 24872877 10.1186/2047-217X-3-7 PMC4035669

[CIT0049] Wan XY, Wan JM, Weng JF, Jiang L, Bi JC, Wang CM, Zhai HQ 2005 Stability of QTLs for rice grain dimension and endosperm chalkiness characteristics across eight environments. Theoretical and Applied Genetics 110, 1334–1346. 15809851 10.1007/s00122-005-1976-x

[CIT0050] Wang E, Wang J, Zhu X, et al 2008 Control of rice grain-filling and yield by a gene with a potential signature of domestication. Nature Genetics 40, 1370–1374. 18820698 10.1038/ng.220

[CIT0051] Wang J, Wan X, Li H, Pfeiffer WH, Crouch J, Wan J 2007 Application of identified QTL-marker associations in rice quality improvement through a design-breeding approach. Theoretical and Applied Genetics 115, 87–100. 17479243 10.1007/s00122-007-0545-x

[CIT0052] Wang S-B, Feng J-Y, Ren W-L, Huang B, Zhou L, Wen Y-J, Zhang J, Dunwell JM, Xu S, Zhang Y-M 2016a Improving power and accuracy of genome-wide association studies via a multi-locus mixed linear model methodology. Scientific Reports 6, 19444. 26787347 10.1038/srep19444 PMC4726296

[CIT0053] Wang W, Mauleon R, Hu Z, et al 2018 Genomic variation in 3,010 diverse accessions of Asian cultivated rice. Nature 557, 43–49. 29695866 10.1038/s41586-018-0063-9 PMC6784863

[CIT0054] Wang X, Pang Y, Wang C, Chen K, Zhu Y, Shen C, Ali J, Xu J, Li Z 2016b New candidate genes affecting rice grain appearance and milling quality detected by genome-wide and gene-based association analyses. Frontiers in Plant Science 7, 1998. 28101096 10.3389/fpls.2016.01998 PMC5209347

[CIT0055] Wang Y, Tang H, Debarry JD, et al 2012 MCScanX: a toolkit for detection and evolutionary analysis of gene synteny and collinearity. Nucleic Acids Research 40, e49. 22217600 10.1093/nar/gkr1293 PMC3326336

[CIT0056] Wen YJ, Zhang H, Ni YL, Huang B, Zhang J, Feng JY, Wang SB, Dunwell JM, Zhang YM, Wu R 2018 Methodological implementation of mixed linear models in multi-locus genome-wide association studies. Briefings in Bioinformatics 19, 700–712. 28158525 10.1093/bib/bbw145 PMC6054291

[CIT0057] Weng J, Gu S, Wan X, et al 2008 Isolation and initial characterization of GW5, a major QTL associated with rice grain width and weight. Cell Research 18, 1199–1209. 19015668 10.1038/cr.2008.307

[CIT0058] Woo MO, Ham TH, Ji HS, et al 2008 Inactivation of the *UGPase1* gene causes genic male sterility and endosperm chalkiness in rice (*Oryza sativa* L.). The Plant Journal 54, 190–204. 18182026 10.1111/j.1365-313X.2008.03405.x PMC2327258

[CIT0059] Yamakawa H, Hirose T, Kuroda M, Yamaguchi T 2007 Comprehensive expression profiling of rice grain filling-related genes under high temperature using DNA microarray. Plant Physiology 144, 258–277. 17384160 10.1104/pp.107.098665 PMC1913800

[CIT0060] Ynion J, Demont M, Custodio MC, Sarkar MAR 2016 Report on the IGA experiment conducted in Rangpur and Jessore, Bangladesh. Los Baños, Philippines: International Rice research Institute.

[CIT0061] Ynion J, Demont M, Samaddar A, Custodio MC 2015 Report on the IGA experiment, conducted in West Bengal and Odisha, India. Los Baños, Philippines: International Rice research Institute.

[CIT0062] Yun P, Zhu Y, Wu B, Gao G, Sun P, Zhang Q, He Y 2016 Genetic mapping and confirmation of quantitative trait loci for grain chalkiness in rice. Molecular Breeding 36, 162.

[CIT0063] Zhang J, Chen LL, Xing F, et al 2016 Extensive sequence divergence between the reference genomes of two elite *indica* rice varieties Zhenshan 97 and Minghui 63. Proceedings of the National Academy of Sciences, USA 113, E5163–5171. 10.1073/pnas.1611012113 PMC5024649 27535938

[CIT0064] Zhao DS, Li QF, Zhang CQ, Zhang C, Yang QQ, Pan LX, Ren XY, Lu J, Gu MH, Liu QQ 2018 GS9 acts as a transcriptional activator to regulate rice grain shape and appearance quality. Nature Communications 9, 1240. 10.1038/s41467-018-03616-y PMC5869696 29588443

[CIT0065] Zhao X, Daygon VD, McNally KL, Hamilton RS, Xie F, Reinke RF, Fitzgerald MA 2016 Identification of stable QTLs causing chalk in rice grains in nine environments. Theoretical and Applied Genetics 129, 141–153. 26498441 10.1007/s00122-015-2616-8

[CIT0066] Zhao X, Fitzgerald M 2013 Climate change: implications for the yield of edible rice. PLoS ONE 8, e66218. 23776635 10.1371/journal.pone.0066218 PMC3680399

[CIT0067] Zhao X, Zhou L, Ponce K, Ye G 2015 The usefulness of known genes/QTLs for grain quality traits in an *indica* population of diverse breeding lines tested using association analysis. Rice 8, 29. 26391157 10.1186/s12284-015-0064-3 PMC4577492

[CIT0068] Zhou L, Liang S, Ponce K, Marundon S, Ye G, Zhao X 2015 Factors affecting head rice yield and chalkiness in *indica* rice. Field Crops Research 172, 1–10.

[CIT0069] Zhu A, Zhang Y, Zhang Z, et al 2018 Genetic dissection of *qPCG1* for a quantitative trait locus for percentage of chalky grain in rice (*Oryza sativa* L.). Frontiers in Plant Science 9, 1173. 30147703 10.3389/fpls.2018.01173 PMC6095994

[CIT0070] Zohoun EV, Tang EN, Soumanou MM, Manful J, Akissoe NH, Bigoga J, Futakuchi K, Ndindeng SA 2018 Physicochemical and nutritional properties of rice as affected by parboiling steaming time at atmospheric pressure and variety. Food Science & Nutrition 6, 638–652. 29876115 10.1002/fsn3.600 PMC5980200

