# Supplementary material for: Dissecting the genome-wide genetic variants of milling and appearance quality traits in rice
Source: J Exp Bot. 2019 May 30;70(19):5115–30. doi: 10.1093/jxb/erz256 (PMC6793453; doi:10.1093/jxb/erz256)
Supplement: erz256_suppl_Supplementary_Figures_S1-S11 [file erz256_suppl_supplementary_figures_s1-s11.pdf]

Dissecting the genome-wide genetic variants of milling and appearance quality traits in rice

Gopal Misra[1], Roslen Anacleto[1], Saurabh Badoni[1], Vito Butardo Jr[1,3], Lilia Molina[1], Andreas Graner[2], Matty Demont[1], Matthew K Morell[1], and Nese Sreenivasulu[1]

[1]International Rice Research Institute, DAPO Box 7777, Metro Manila 1301, Philippines

[2]Leibniz Institute of Plant Genetics and Crop Plant Research (IPK), Corrensstrasse 3, 06466 Seeland OT Gatersleben, Germany

[3]Present address: Department of Chemistry and Biotechnology, Faculty of Science, Engineering and Technology, Swinburne University of Technology, Hawthorn, Victoria, 3122, Australia

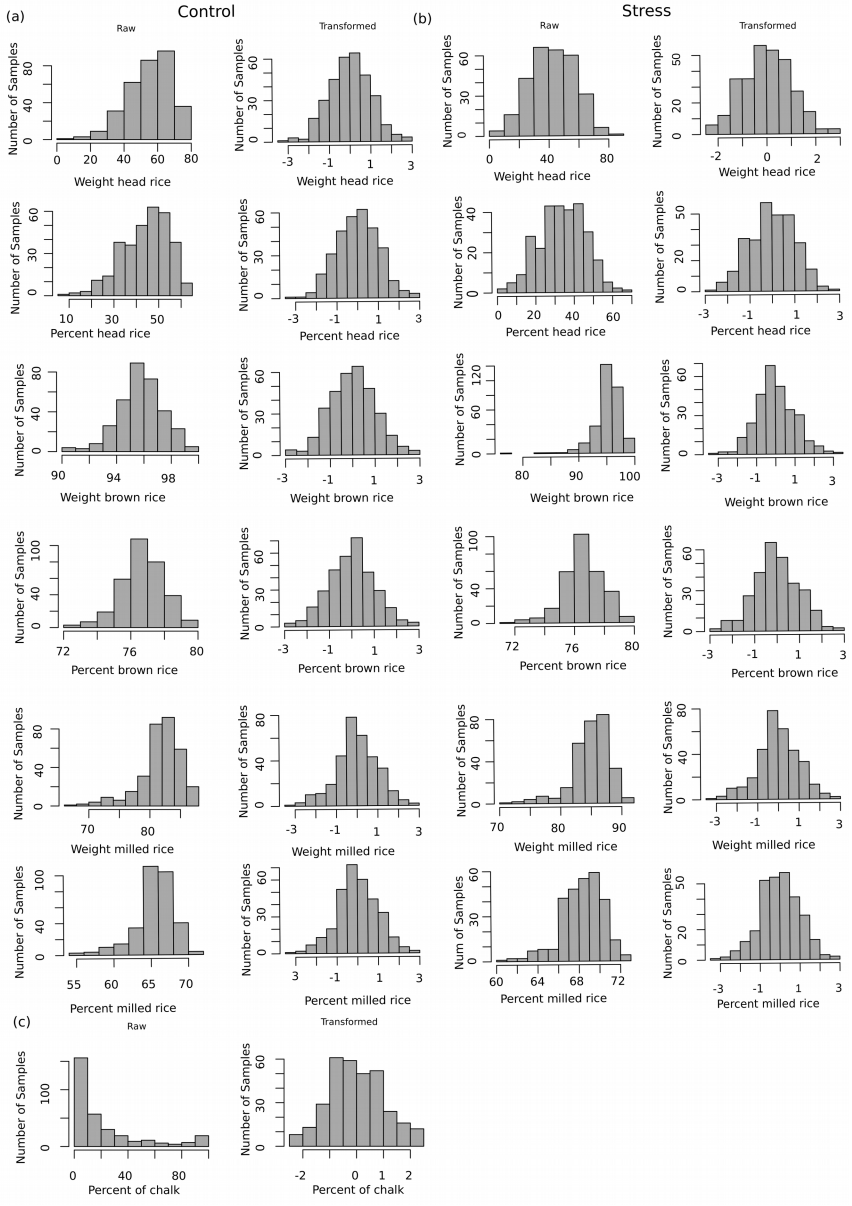

Figure S1: Phenotypic variation for milling quality traits and chalkiness in 320 indica panel. Raw and transformed phenotypic values for weight head rice, head rice yield, weight brown rice, percent brown rice, weight milled rice and percent milled rice, in controlled condition (a) and moister stressed condition (b); (c) Raw and transformed phenotypic value for percent grain chalkiness. X-axis represents respective absolute (in raw) or transformed trait values and y-axis represents the number of samples. All of the traits matched with the normal distribution pattern after getting transformed.

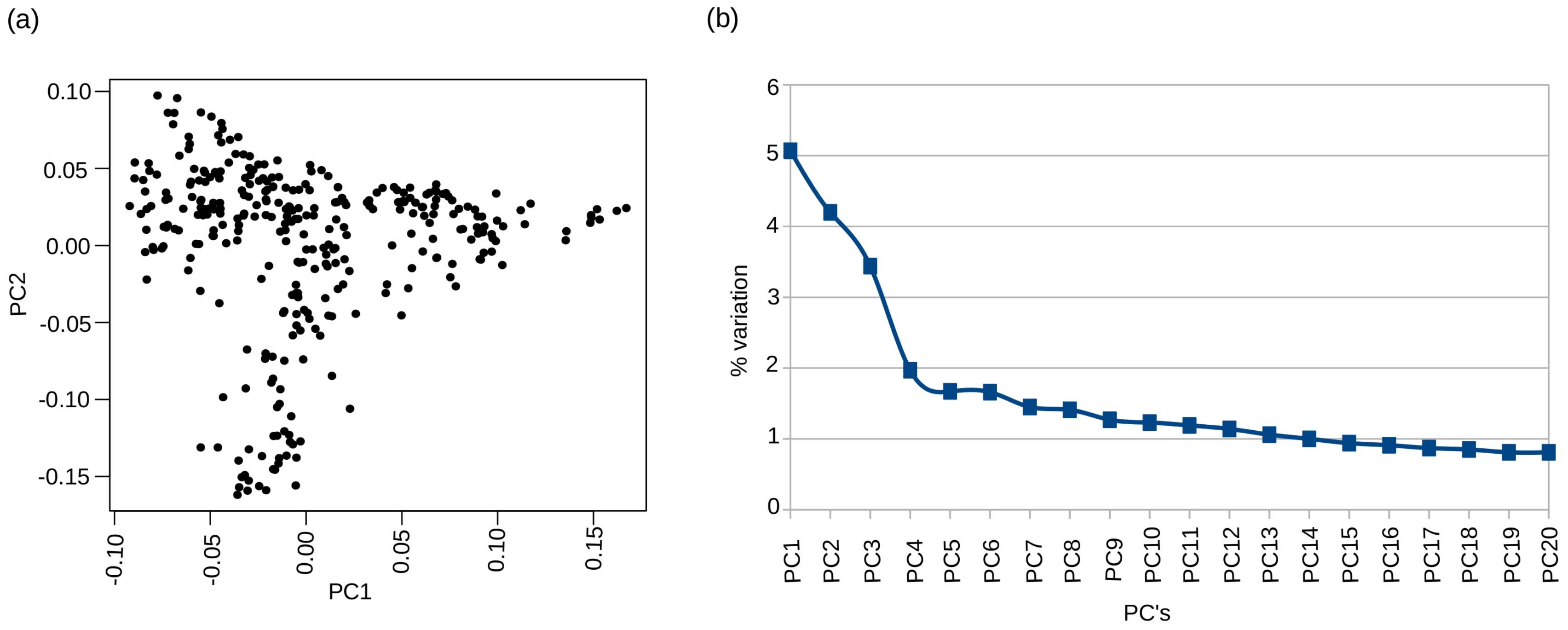

Figure S2: Principal component analysis (PCA) of 320 resequenced lines. (a) Biplot of the first two components, principal component (PC) 1, and PC2 based on 2.26 million high-dense SNPs. (b) Representation of percent variation explained in each PC as scree plot. First 4 PC's were detected to explain major proportion (14%) of the total variations.

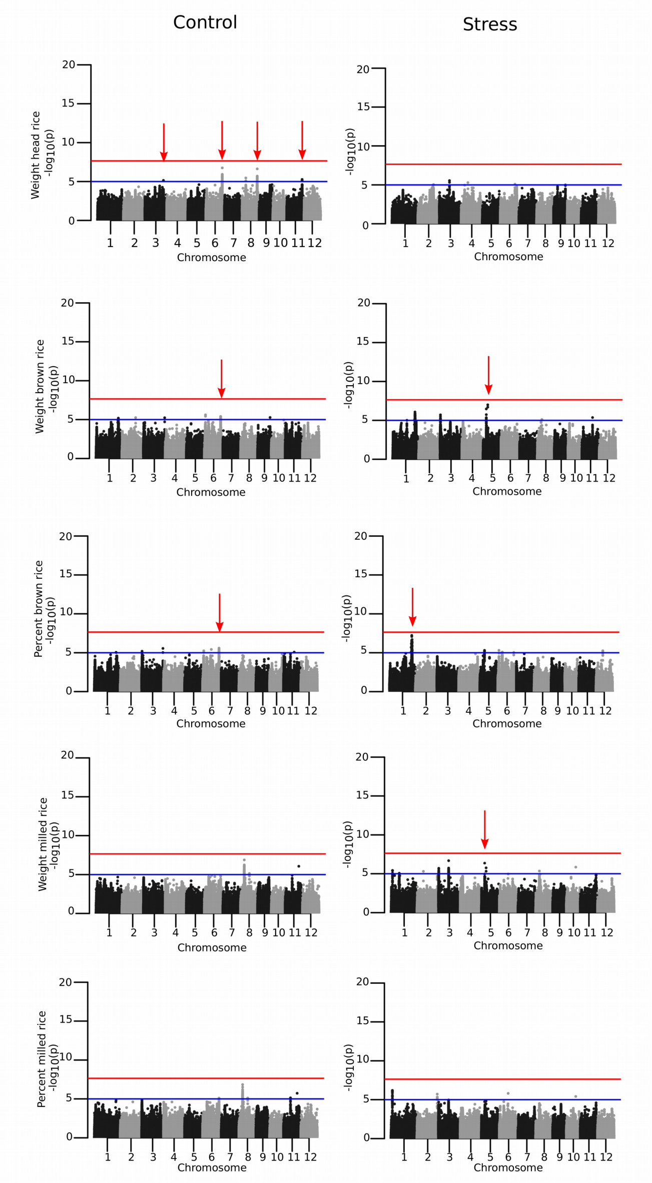

Figure S3: Manhattan-plots generated in GWAS for milling quality traits within *indica* germplasm. Manhattan plots generated for the output of single-locus GWAS for control and moister stress conditions were further validated using the multi-locus GWAS, and marked by red vertical arrow; Head rice yield (HRY) (a); weight head rice (WHR) (b); percent milled rice (PMR) (c); weight milled rice (WMR) (d); percent brown rice (PBR) (e) and weight brown rice (WBR) (f). Horizontal red and blue line in Manhattan plots represents the genome-wide significant threshold $-\log_{10}(P)$ value of 7.5 and 5, respectively.

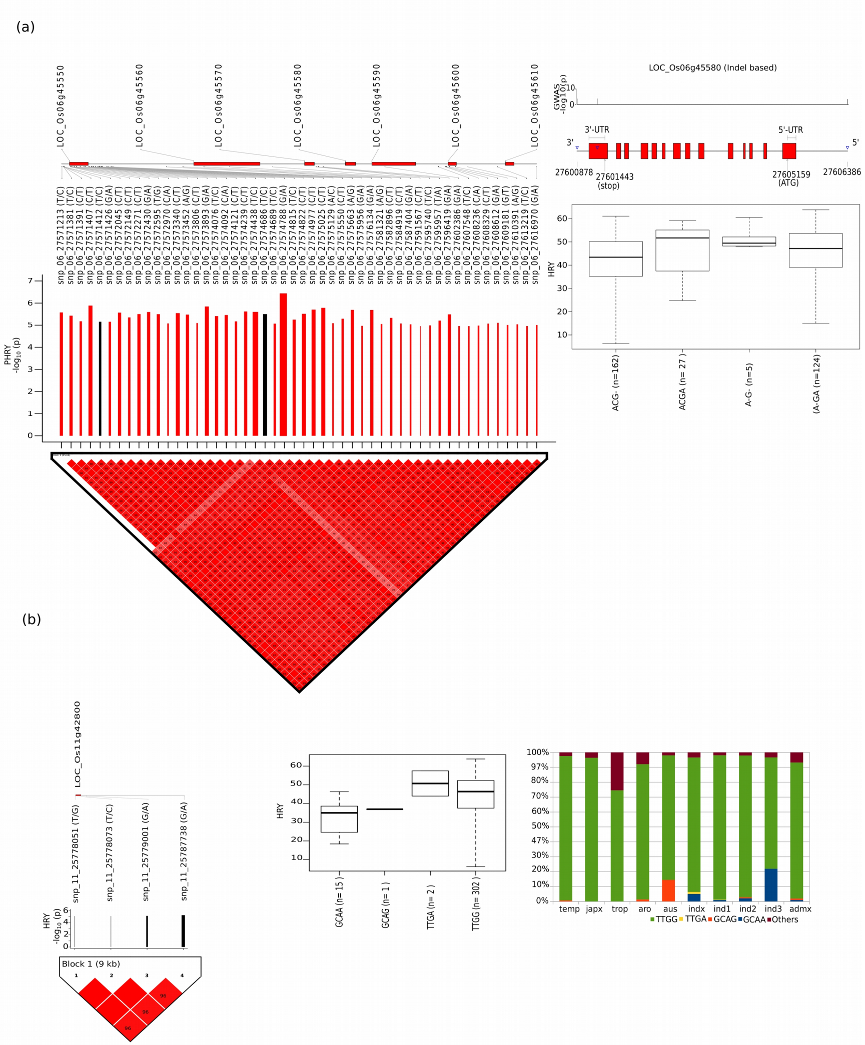

Figure S4: Single-locus GWAS for head rice yield (HRY) revealed the association of chromosome 6 and 11 genomic regions. (a) Linkage disequilibrium (LD) plot of the tag SNPs on chromosome 6 significantly associated with HRY. A scaled and highly dense LD-based plot within the hotspot genomic region on the chromosome is represented. Haplotypes constructed based on presence of Indels within neighboring region of LOC_Os06g45590, and their respective HRY phenotypic values were represented as boxplot. Deletion is mentioned as a dash (-) in haplotypes; (b) the linkage disequilibrium (LD) plot of 4 tagged SNP on chromosome 11 significantly associated with HRY. The positions of the tagged SNPs marked with the $\log_{10}$-scaled P-values ($\log_{10}(P)$) and black/red bars reflecting their relative positive/negative effect sizes, respectively. Haplotypes with phenotypic values for HRY are represented as boxplot; the distribution of these haplotypes was further examined with data from the 3000 Rice Genomes Project (2014).

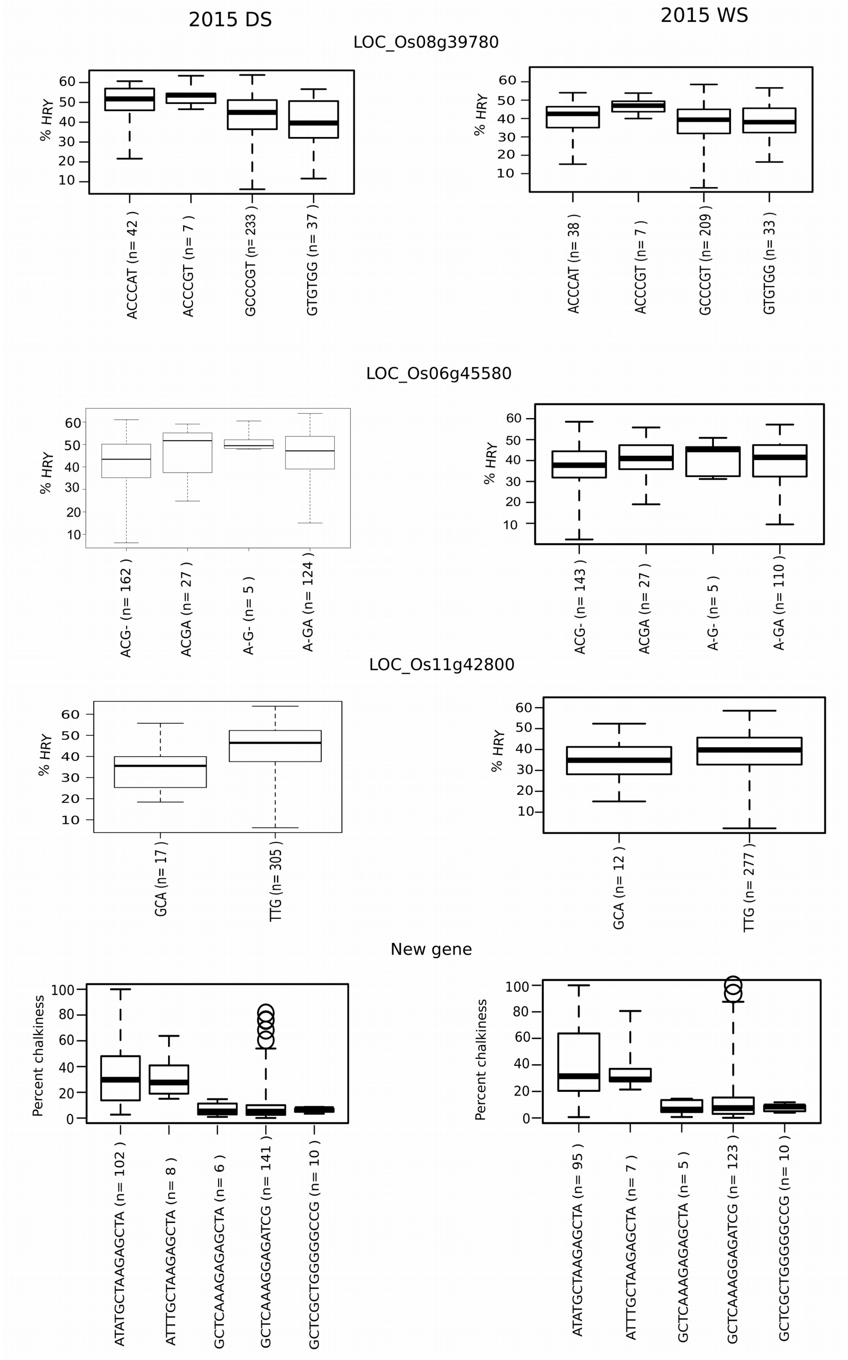

Figure S5: Variation in the phenotypic values of percent HRY and chalkiness observed for different haplotypes across dry and wet seasons. The phenotypic variations for % HRY in 2015DS and 2015WS were reflected by haplotypes identified in the candidates LOC_Os08g39780, LOC_Os06g45580 and LOC_Os11g42800 on chromosome 8, 6 and 11, respectively. Similarly, for percent chalkiness, haplotypes identified within a new gene (Chalk5.1) also depicted the consistency in the phenotype across the seasons.

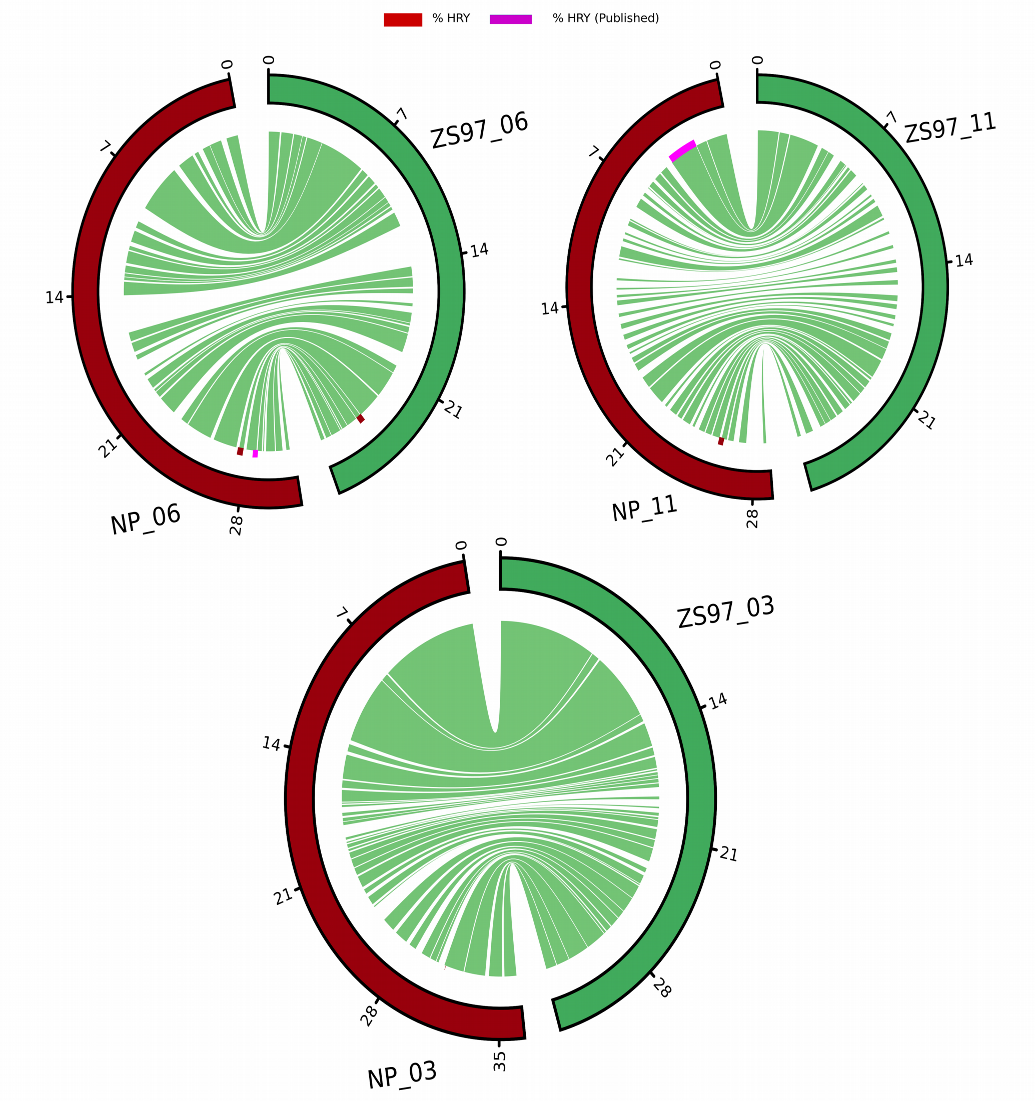

Figure S6: Comprehensive representation of structural variation and collinearity between *japonica* cultivar Nipponbare and *indica* cultivar ZS97. Circos plots depicted collinearity in chromosomes 3, 6 and 11(physical size shown in Mb), within *both* subspecies based on protein sequence alignment. The significant hotspot regions for HRY detected in our study (red colour) and previous studies (pink colour) were mapped on respective genomic positions. Likewise, the genomic region regulating chalk is represented in blue colour in the synteny map (circos). The conserved regions were represented in green lines, while, white gap in the region signifies the collinearity break in the region.

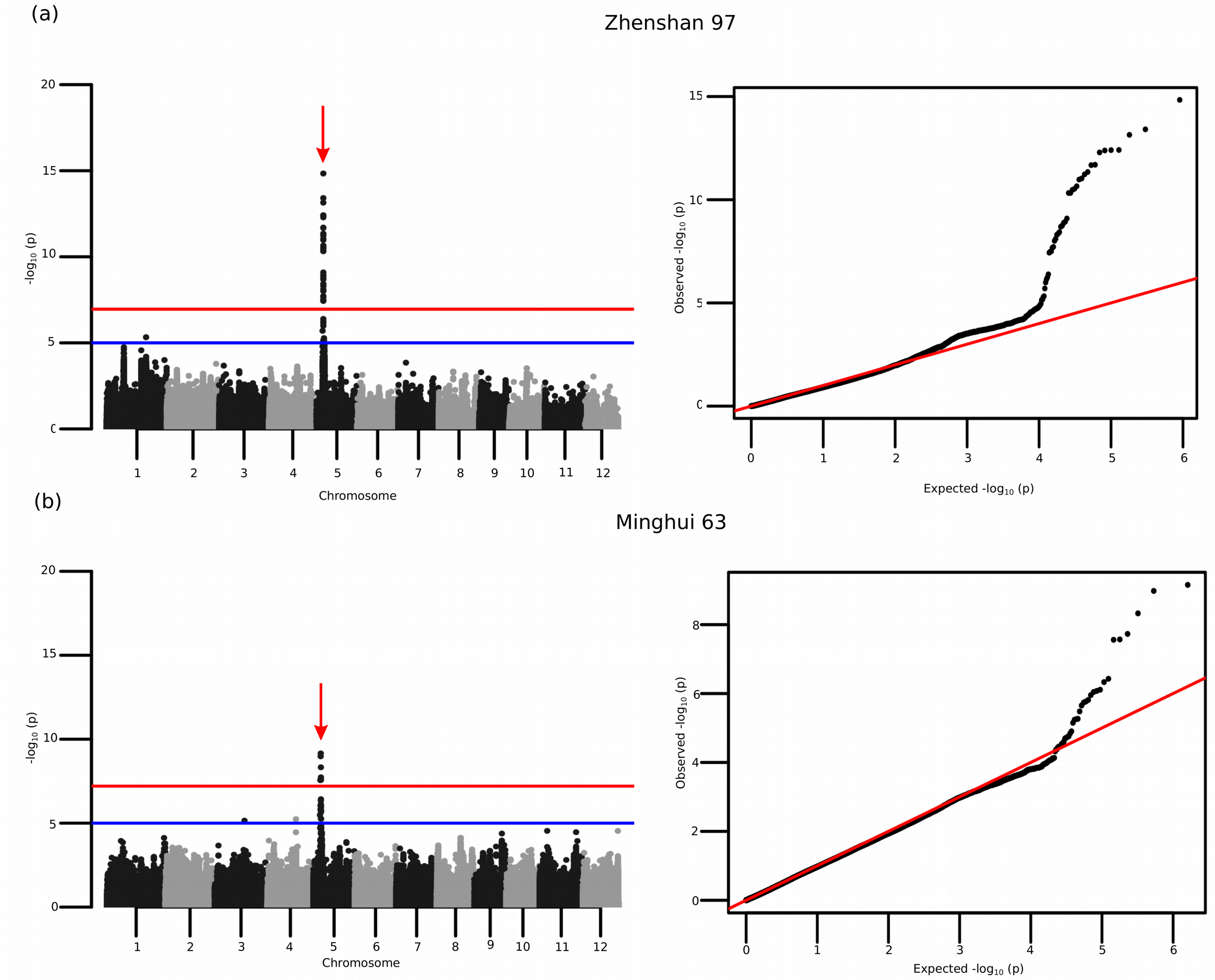

Figure S7: Single-locus GWAS for grain chalkiness identified prominent candidate loci on chromosomes 5 using *indica* reference genomes Zhanshan 97 (ZS97) and Minghui 63 (MH63). Manhattan and QQ- plots of the GWAS conducted on percent grain with chalkiness (PGC) in *indica* germplasm panel using ZS97 (a) and MH63 (b) reference genomes, respectively. A association peak on chromosome 5 was identified using both references and further validated using the multi-locus GWAS, and marked by red vertical arrow; Horizontal red and blue line in Manhattan plots represents the genome-wide significant threshold -$\log_{10}(P)$ value of 7 and 5, respectively.

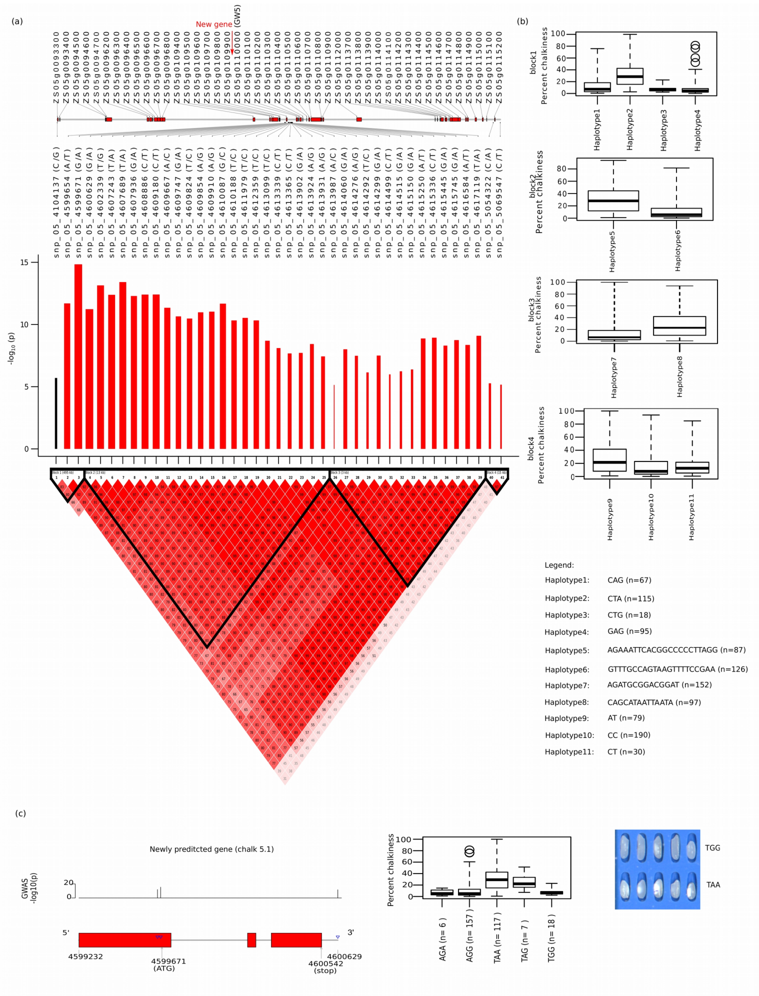

Figure S8: Single-locus GWAS for PGC revealed the significant association on chromosome 5 hotspot region to PGC using Zhenshan97 (*indica*) as a reference genome. (a) Scaled linkage disequilibrium (LD) plot of the 41 tagged-SNPs in the hotspot genomic region significantly associated with PGC and the position of newly predicted gene is highlighted in red arrow. The 41 tagged-SNPs were indicated with the log10-scaled P values (log10(P)) and black/red bars reflecting their relative positive/negative effect sizes, respectively, (b) 11 haplotypes constructed within 4 LD-blocks and their respective phenotypic values for PGC are represented as boxplots with haplotype allelic combination underneath. (c) TGAS of newly predicted gene (termed as chalk 5.1) identified the significant low chalk haplotype TGG, which is represented in boxplots with rest haplotype combinations, leftmost is the predicted gene modal with identified key SNPs while rightmost section depicted the image of 5 lines from each of two selected haplotypes showing grain phenotype.

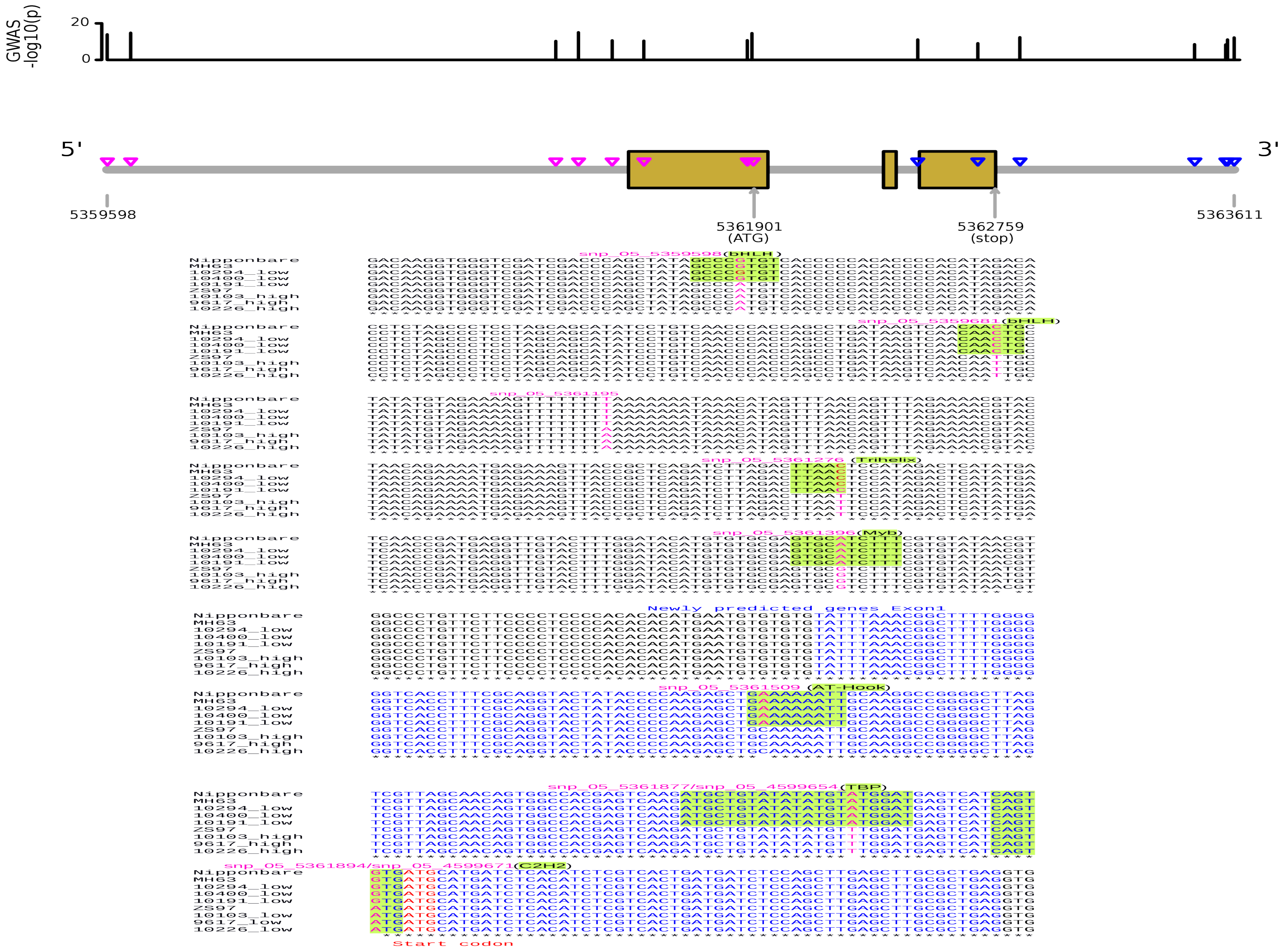

GWAS
-log10(p)
20
0
5'
3'
5359598
5361901
(ATG)
5362759
(stop)
5363611
Nipponbare
MH63
10294_low
10400_low
10191_low
ZS97
10103_high
9617_high
10226_high
snp_05_5359598(bHLH)
snp_05_5359681(bHLH)
snp_05_5361195
snp_05_5361276 (Trihelix)
snp_05_5361396(Myb)
Newly predicted genes Exon1
snp_05_5361509 (AT-Hook)
snp_05_5361877/snp_05_4599654(TBP)
snp_05_5361894/snp_05_4599671(C2H2)
10103_low
9617_low
10226_low
Start codon

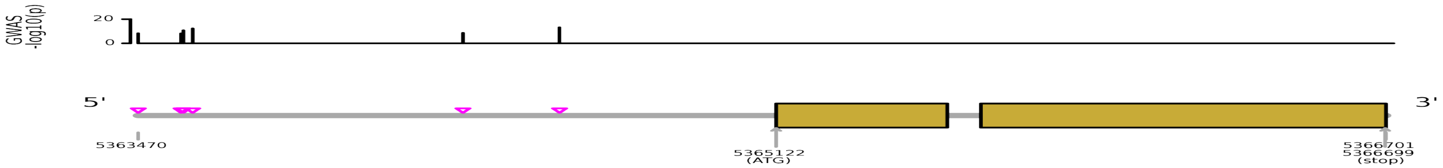


```
                        snp_05_5363470
Nipponbare      GGGGTAGATCGACTCCCAGAAATCGGCGGCGGCCGGACCTGGACCGGCTACGGGAAGGGC
MH63            GGGGTAGATCGACTCCCAGAAATCGGCGGCGGCCGGACCTGGACCGGCTACGGGAAGGGC
10294_low       GGGGTAGATCGACTCCCAGAAATCGGCGGCGGCCGGACCTGGACCGGCTACGGGAAGGGC
10400_low       GGGGTAGATCGACTCCCAGAAATCGGCGGCGGCCGGACCTGGACCGGCTACGGGAAGGGC
10191_low       GGGGTAGATCGACTCCCAGAAATCGGCGGCGGCCGGACCTGGACCGGCTACGGGAAGGGC
ZS97            GGGGTAGATCGACTCCCAGAAGTCGGCGGCGGCCGGACCTGGACCGGCTACGGGAAGGGC
10103_high      GGGGTAGATCGACTCCCAGAAGTCGGCGGCGGCCGGACCTGGACCGGCTACGGGAAGGGC
9617_high       GGGGTAGATCGACTCCCAGAAGTCGGCGGCGGCCGGACCTGGACCGGCTACGGGAAGGGC
10226_high      GGGGTAGATCGACTCCCAGAAGTCGGCGGCGGCCGGACCTGGACCGGCTACGGGAAGGGC
                ********************* **************************************

                snp_05_5363581(AP2)  snp_05_5363587(AP2)  snp_05_5363611(Tify)
Nipponbare      CGAGATTGGCTGTGCCGCCGCCAGGGACGACGGAAGGGGATCGGGAAGAGACAGATTACC
MH63            CGAGATTGGCTGTGCCGCCGCCAGGGACGACGGAAGGGGATCGGGAAGAGACAGATTACC
10294_low       CGAGATTGGCTGTGCCGCCGCTAGGGATGACGGAAGGGGATCGGGAAGAGACAGATTACC
10400_low       CGAGATTGGCTGTGCCGCCGCTAGGGATGACGGAAGGGGATCGGGAAGAGACAGATTACC
10191_low       CGAGATTGGCTGTGCCGCCGCCAGGGACGACGGAAGGGGATCGGGAAGAGACAGATTACC
ZS97            CGAGATTGGCTGCGCCGCTGCCAGGGACGACGGAAGGGGATCAGGAAGAGACAGATTACC
10103_high      CGAGATTGGCTGCGCCGCTGCCAGGGACGACGGAAGGGGATCAGGAAGAGACAGATTACC
9617_high       CGAGATTGGCTGCGCCGCTGCCAGGGACGACGGAAGGGGATCAGGAAGAGACAGATTACC
10226_high      CGAGATTGGCTGCGCCGCTGCCAGGGACGACGGAAGGGGATCAGGAAGAGACAGATTACC
                ************ ***** ** ***** ************** *****************

                           snp_05_5364311(Tify)
Nipponbare      TGGGCTTACACTGTCCCTTTTTTATCGCTGCGTCCAATCCTAATCCACTATTTATGCCTA
MH63            TGGGCTTACACTGTCCCTTTTTTATCGCTGCGTCCAATCCTAATCCACTATTTATGCCTA
10294_low       TGGGCTTACACTGTCCCTTTTTTATCGCTGCGTCCAATCCTAATCCACTATTTATGCCTA
10400_low       TGGGCTTACACTGTCCCTTTTTTATCGCTGCGTCCAATCCTAATCCACTATTTATGCCTA
8674_low        TGGGCTTACACTGTCCCTTTTTTATCGCTGCGTCCAATCCTAATCCACTATTTATGCCTA
10191_low       TGGGCTTACACTGTCCCTTTTTTATCGCTGCGTCCAATCCTAATCCACTATTTATGCCTA
ZS97            TGGGCTTACACTGTCCCTTTTTGATCGCTGCGTCCAATCCTAATCCACTATTTATGCCTA
10103_high      TGGGCTTACACTGTCCCTTTTTGATCGCTGCGTCCAATCCTAATCCACTATTTATGCCTA
9617_high       TGGGCTTACACTGTCCCTTTTTGATCGCTGCGTCCAATCCTAATCCACTATTTATGCCTA
10226_high      TGGGCTTACACTGTCCCTTTTTGATCGCTGCGTCCAATCCTAATCCACTATTTATGCCTA
                ********************** *************************************

                                  snp_05_5364561 (MADF)
Nipponbare      AATGGTCTGAATTGTTCGTTATTAAGGGTAAATTATAAATTTACTAATATAATGATTACG
MH63            AATGGTCTGAATTGTTCGTTATTAAGGGTAAATTATAAATTTACTAATATAATGATTACG
10294_low       AATGGTCTGAATTGTTCGTTATTAAGGGTAAATTATAAATTTACTAATATAATGATTACG
10400_low       AATGGTCTGAATTGTTCGTTATTAAGGGTAAATTATAAATTTACTAATATAATGATTACG
10191_low       AATGGTCTGAATTGTTCGTTATTAAGGGTAAATTATAAATTTACTAATATAATGATTACG
ZS97            AATGGTCTGAATTGTTCGTTATTAAGGGTAAAGTATAAATTTACTAATATAATGATTACG
10103_high      AATGGTCTGAATTGTTCGTTATTAAGGGTAAAGTATAAATTTACTAATATAATGATTACG
9617_high       AATGGTCTGAATTGTTCGTTATTAAGGGTAAAGTATAAATTTACTAATATAATGATTACG
10226_high      AATGGTCTGAATTGTTCGTTATTAAGGGTAAAGTATAAATTTACTAATATAATGATTACG
                ******************************** ***************************
```

Figure S9: Nucleotide sequence alignment in the prominent chromosome 5 hotspot regions within cultivars with extreme chalk phenotypes. (a) Highly significant SNPs identified using TGAS in the newly predicted gene (*chalk5.1*) especially in the upstream region were represented in the gene model. Below, genome sequence of newly predicted gene region (*chalk5.1*) within three lines, possessing low (10294, 10400, 10191) and high chalky phenotypes (10103, 9617, 10226) were aligned with the three cultivars, Nipponbare (Intermediate chalk), ZS97 (high chalk) and MH63 (Low chalk), which were also used as reference genomes in present study. Highly significant allelic variations identified using the Nipponbare reference, in the promotor and 5'-UTR region, were observed consistent with their chalk phenotype; Significant SNPs observed in promotor region were predicted within the binding site of bHLH protein (snp_05_5359598, snp_05_5359681), tri-helix protein (topmost associated SNP; snp_05_5361276) and Myb-factor binding site (snp_05_5361396), whereas SNPs lying in 5'-UTR region were predicted within binding site of AT-hook (snp_05_5361509), TATA-binding protein (same SNP detected against Nipponbare references, snp_05_5361877; and ZS97 reference, snp_05_4599654) and C2H2-binding site (same SNP detected against Nipponbare references, snp_05_5361894; and ZS97 reference, snp_05_4599671). (b) Significant SNPs identified using TGAS in the upstream region of *GW5* (LOC_OS05g09520) gene, which harbour the binding site for transcription factor including AP2, TIFY, C2H2 and MADF. Blue highlighted region corresponds to 5'-UTR region and nucleotides highlighted in green signifies the corresponding transcription factor binding site.

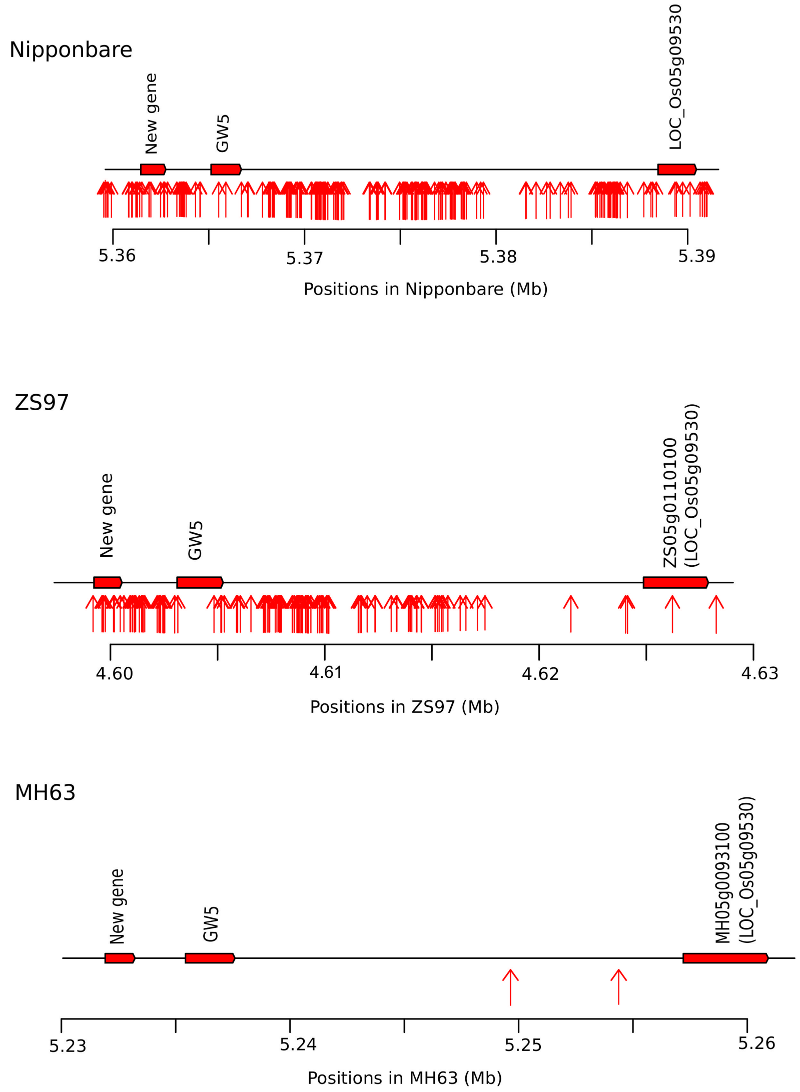

Figure S10: SNPs in the genomic sequence of chromosome 5 hotspot region regulating PGC, in the three cultivars (Nipponbare, ZS97, and MH63), used as reference genomes in the present study. In the genomic sequence of MH63, the very few SNPs are detected in the key genomic region, whereas, unlike MH63, in case of other ZS97 and Nippponbare, hotspot region possess sufficient SNPs, enabling the detection of three candidate genes regulating PGC. In the case of ZS97 and MH63, the gene ID in parenthesis showed the corresponding gene ID from the Nipponbare reference genome.

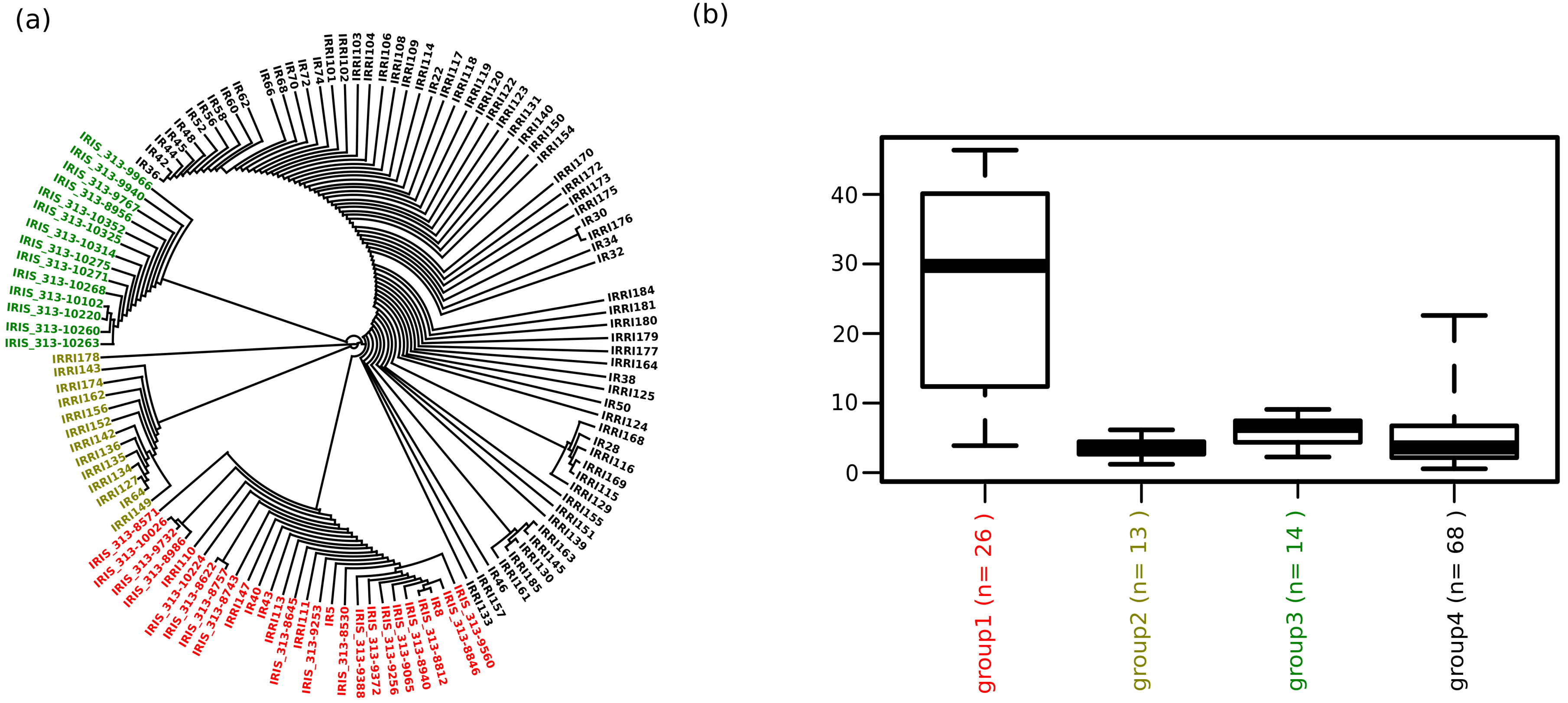

Figure S11: Allelic variations exist in chromosome 5 hotspot region and their distribution in IRRI breeding lines and selected core-collection cultivars. Unrooted dendrogram (a) and boxplot (b) for haplotypes of chromosome 5 hotspot region associated with percent grain chalkiness (PGC), group-1 (in red colour) clustered lines identified as high chalky (median 29.7%) consist of cultivars with high-chalk haplotype from core collection and IRRI breeding lines with high chalk phenotype, group-2 (yellowish green color) showed the low chalk lines (median 3.6%) including mega-varieties like IR-64, group-3 (green colour) depicted low chalkiness (median 6.7%) grouped the cultivars possessing low chalk haplotype lines from core collection and group-4 was represented in low chalk lines (median 3.6%) showing relatively high variance for PGC.
