# Supplementary material for: Dissecting the genome-wide genetic variants of milling and appearance quality traits in rice
Source: J Exp Bot. 2019 May 30;70(19):5115–30. doi: 10.1093/jxb/erz256 (PMC6793453; doi:10.1093/jxb/erz256)
Supplement: erz256_suppl_Supplementary_Tables_S1-S7 [file erz256_suppl_supplementary_tables_s1-s7.pdf]

Table S1

**Table S1: Accessions used in the study, with the exiting haplotypes for HRY in hotspot regions of chromosome 8 and 11.**

**Favourable haplotype identified for high HRY**

- **Missing/heterozygous**

| Accession IDs | chr8 (block1) | chr11 | Average % HRY |
|---|---|---|---|
| IRIS_313-10000 | TTATTATTATATTA | TTGG | 57.50 |
| IRIS_313-10001 | CCGCCGGCGCCCCG | TTGG | 35.43 |
| IRIS_313-10007 | TTATTATTATATTA | TTGG | 46.87 |
| IRIS_313-10016 | CCGCCGGCGCCCCG | TTGG | 35.10 |
| IRIS_313-10026 | CCGCCGGCGCCCCG | TTGG | 24.23 |
| IRIS_313-10030 | CCGCCGGCGCCCCG | TTGG | 14.17 |
| IRIS_313-10034 | CCGCCGGCGCCCCG | TTGG | 36.40 |
| IRIS_313-10040 | CCGCCGGCGCCCCG | TTGG | 63.33 |
| IRIS_313-10045 | CCGCCGGCGCCCCG | TTGG | 28.13 |
| IRIS_313-10046 | CCGCCGGCGCCCCG | TTGG | 53.37 |
| IRIS_313-10047 | CCGCCGGCGCCCCG | TTGG | 39.43 |
| IRIS_313-10048 | CCGCCGGCGCCCCG | TTGG | 32.73 |
| IRIS_313-10050 | CCGCCGGCGCCCCG | TTGG | 35.73 |
| IRIS_313-10054 | CCGCCGGCGCCCCG | TTGG | 45.03 |
| IRIS_313-10102 | - | TTGG | 41.77 |
| IRIS_313-10103 | CCGCCGGCGCCCCG | TTGG | 32.97 |
| IRIS_313-10109 | CCGCCGGCGCCCCG | TTGG | 35.23 |
| IRIS_313-10113 | - | TTGG | 53.47 |
| IRIS_313-10114 | CCGCCGGCGCCCCG | TTGG | 13.07 |
| IRIS_313-10129 | CCGCCGGCGCCCCG | TTGG | 41.60 |
| IRIS_313-10147 | CCGCCGGCGCCCCG | TTGG | 43.17 |
| IRIS_313-10148 | CCGCCGGCGCCCCG | TTGG | 55.00 |
| IRIS_313-10151 | - | - | 51.07 |
| IRIS_313-10154 | CCGCCGGCGCCCCG | TTGG | 43.13 |
| IRIS_313-10167 | CCGCCGGCGCCCCG | TTGG | 38.93 |
| IRIS_313-10168 | CCGCCGGCGCCCCG | GCAA | 26.10 |
| IRIS_313-10170 | CCGCCGGCGCCCCG | TTGG | 36.33 |
| IRIS_313-10171 | CCGCCGGCGCCCCG | TTGG | 40.93 |
| IRIS_313-10177 | CCGCCGGCGCCCCG | **TTGA** | 57.40 |
| IRIS_313-10178 | CCGCCGGCGCCCCG | TTGG | 50.43 |
| IRIS_313-10179 | CCGCCGGCGCCCCG | TTGG | 52.43 |
| IRIS_313-10191 | TTATTATTATATTA | TTGG | 59.73 |
| IRIS_313-10220 | - | TTGG | 48.90 |
| IRIS_313-10221 | TTATTATTATATTA | TTGG | 59.13 |
| IRIS_313-10224 | CCGCCGGCGCCCCG | TTGG | 40.43 |
| IRIS_313-10226 | CCGCCGGCGCCCCG | TTGG | 45.57 |
| IRIS_313-10234 | TTATTATTATATTA | TTGG | 42.80 |
| IRIS_313-10235 | CCGCCGGCGCCCCG | TTGG | 47.90 |
| IRIS_313-10237 | TTATTATTATATTA | TTGG | 48.67 |
| IRIS_313-10238 | TTATTATTATATTA | TTGG | 60.97 |
| IRIS_313-10247 | CCGCCGGCGCCCCG | TTGG | 45.50 |
| IRIS_313-10257 | CCGCCGGCGCCCCG | TTGG | 30.73 |
| IRIS_313-10260 | CCGCCGGCGCCCCG | TTGG | 55.20 |
| IRIS_313-10263 | CCGCCGGCGCCCCG | TTGG | 39.73 |
| IRIS_313-10268 | TTATTATTATATTA | TTGG | 50.13 |
| IRIS_313-10271 | CCGCCGGCGCCCCG | TTGG | 48.67 |
| IRIS_313-10274 | - | TTGG | 59.43 |

Table S1

| Accession IDs | chr8 (block1) | chr11 | Average % HRY |
|---|---|---|---|
| IRIS_313-10275 | - | TTGG | 40.20 |
| IRIS_313-10279 | CCGCCGGCGCCCCG | TTGG | 30.60 |
| IRIS_313-10285 | - | TTGG | 46.90 |
| IRIS_313-10287 | TTATTATTATATTA | TTGG | 54.07 |
| IRIS_313-10289 | CCGCCGGCGCCCCG | TTGG | 57.03 |
| IRIS_313-10293 | CCGCCGGCGCCCCG | TTGG | 46.00 |
| IRIS_313-10294 | CCGCCGGCGCCCCG | TTGG | 46.40 |
| IRIS_313-10298 | CCGCCGGCGCCCCG | TTGG | 31.60 |
| IRIS_313-10300 | CCGCCGGCGCCCCG | TTGG | 53.27 |
| IRIS_313-10301 | CCGCCGGCGCCCCG | TTGG | 43.73 |
| IRIS_313-10307 | - | TTGG | 52.37 |
| IRIS_313-10314 | CCGCCGGCGCCCCG | TTGG | 48.70 |
| IRIS_313-10318 | CCGCCGGCGCCCCG | TTGG | 44.70 |
| IRIS_313-10325 | CCGCCGGCGCCCCG | TTGG | 40.57 |
| IRIS_313-10332 | CCGCCGGCGCCCCG | TTGG | 58.20 |
| IRIS_313-10333 | CCGCCGGCGCCCCG | TTGG | 49.47 |
| IRIS_313-10334 | CCGCCGGCGCCCCG | TTGG | 49.53 |
| IRIS_313-10337 | CCGCCGGCGCCCCG | GCAA | 33.43 |
| IRIS_313-10340 | TTATTATTATATTA | TTGG | 50.70 |
| IRIS_313-10349 | CCGCCGGCGCCCCG | GCAA | 35.50 |
| IRIS_313-10352 | CCGCCGGCGCCCCG | TTGG | 47.73 |
| IRIS_313-10353 | TTATTATTATATTA | TTGG | 31.27 |
| IRIS_313-10355 | CCGCCGGCGCCCCG | TTGG | 37.33 |
| IRIS_313-10357 | CCGCCGGCGCCCCG | TTGG | 46.37 |
| IRIS_313-10360 | CCGCCGGCGCCCCG | TTGG | 52.67 |
| IRIS_313-10361 | TTATTATTATATTA | TTGG | 59.43 |
| IRIS_313-10366 | CCGCCGGCGCCCCG | TTGG | 48.53 |
| IRIS_313-10371 | CCGCCGGCGCCCCG | TTGG | 31.70 |
| IRIS_313-10374 | TTATTATTATATTA | TTGG | 54.50 |
| IRIS_313-10375 | CCGCCGGCGCCCCG | TTGG | 32.70 |
| IRIS_313-10385 | CCGCCGGCGCCCCG | TTGG | 38.97 |
| IRIS_313-10392 | TTATTATTATATTA | TTGG | 46.37 |
| IRIS_313-10394 | CCGCCGGCGCCCCG | TTGG | 56.80 |
| IRIS_313-10396 | TTATTATTATATTA | TTGG | 55.67 |
| IRIS_313-10397 | CCGCCGGCGCCCCG | TTGG | 57.50 |
| IRIS_313-10398 | CCGCCGGCGCCCCG | TTGG | 59.50 |
| IRIS_313-10399 | CCGCCGGCGCCCCG | TTGG | 51.80 |
| IRIS_313-10400 | CCGCCGGCGCCCCG | TTGG | 46.37 |
| IRIS_313-10401 | - | TTGG | 50.13 |
| IRIS_313-10402 | CCGCCGGCGCCCCG | TTGG | 54.43 |
| IRIS_313-10403 | TTATTATTATATTA | TTGG | 60.50 |
| IRIS_313-10404 | CCGCCGGCGCCCCG | TTGG | 50.20 |
| IRIS_313-10412 | CCGCCGGCGCCCCG | TTGG | 49.70 |
| IRIS_313-10417 | CCGCCGGCGCCCCG | TTGG | 31.30 |
| IRIS_313-10421 | - | TTGG | 54.67 |
| IRIS_313-10422 | - | TTGG | 35.17 |
| IRIS_313-10423 | CCGCCGGCGCCCCG | GCAA | 34.67 |
| IRIS_313-8288 | CCGCCGGCGCCCCG | TTGG | 27.97 |
| IRIS_313-8303 | TTATTATTATATTA | TTGG | 59.37 |
| IRIS_313-8305 | CCGCCGGCGCCCCG | TTGG | 49.73 |
| IRIS_313-8315 | CCGCCGGCGCCCCG | TTGG | 53.60 |
| IRIS_313-8332 | CCGCCGGCGCCCCG | TTGG | 44.27 |
| IRIS_313-8368 | CCGCCGGCGCCCCG | TTGG | 32.97 |
| IRIS_313-8380 | CCGCCGGCGCCCCG | TTGG | 43.10 |

Table S1

| Accession IDs | chr8 (block1) | chr11 | Average % HRY |
|---|---|---|---|
| IRIS_313-8386 | **CCGTTATTATATTA** | TTGG | 51.80 |
| IRIS_313-8392 | CCGCCGGCGCCCCG | TTGG | 30.50 |
| IRIS_313-8401 | - | TTGG | 46.90 |
| IRIS_313-8409 | - | TTGG | 57.40 |
| IRIS_313-8412 | - | TTGG | 49.17 |
| IRIS_313-8414 | CCGCCGGCGCCCCG | TTGG | 39.83 |
| IRIS_313-8433 | CCGCCGGCGCCCCG | TTGG | 52.67 |
| IRIS_313-8435 | CCGCCGGCGCCCCG | TTGG | 34.83 |
| IRIS_313-8437 | CCGCCGGCGCCCCG | TTGG | 52.83 |
| IRIS_313-8450 | **CCGTTATTATATTA** | TTGG | 53.93 |
| IRIS_313-8453 | CCGCCGGCGCCCCG | TTGG | 49.83 |
| IRIS_313-8454 | CCGCCGGCGCCCCG | TTGG | 52.70 |
| IRIS_313-8458 | CCGCCGGCGCCCCG | GCAA | 46.37 |
| IRIS_313-8509 | CCGCCGGCGCCCCG | - | 55.10 |
| IRIS_313-8514 | CCGCCGGCGCCCCG | TTGG | 44.57 |
| IRIS_313-8530 | CCGCCGGCGCCCCG | TTGG | 22.17 |
| IRIS_313-8536 | CCGCCGGCGCCCCG | TTGG | 37.60 |
| IRIS_313-8559 | CCGCCGGCGCCCCG | TTGG | 49.50 |
| IRIS_313-8568 | CCGCCGGCGCCCCG | TTGG | 52.13 |
| IRIS_313-8571 | TTATTATTATATTA | TTGG | 57.10 |
| IRIS_313-8585 | TTATTATTATATTA | TTGG | 56.57 |
| IRIS_313-8586 | TTATTATTATATTA | GCAA | 23.97 |
| IRIS_313-8595 | CCGCCGGCGCCCCG | TTGG | 4.37 |
| IRIS_313-8603 | CCGCCGGCGCCCCG | TTGG | 46.83 |
| IRIS_313-8614 | **CCGTTATTATATTA** | TTGG | 63.13 |
| IRIS_313-8616 | CCGCCGGCGCCCCG | TTGG | 47.37 |
| IRIS_313-8621 | - | TTGG | 37.03 |
| IRIS_313-8622 | - | TTGG | 25.37 |
| IRIS_313-8631 | CCGCCGGCGCCCCG | TTGG | 32.17 |
| IRIS_313-8632 | CCGCCGGCGCCCCG | TTGG | 51.67 |
| IRIS_313-8638 | TTATTATTATATTA | TTGG | 44.13 |
| IRIS_313-8643 | CCGCCGGCGCCCCG | TTGG | 47.60 |
| IRIS_313-8645 | CCGCCGGCGCCCCG | TTGG | 39.23 |
| IRIS_313-8647 | CCGCCGGCGCCCCG | TTGG | 58.10 |
| IRIS_313-8659 | CCGCCGGCGCCCCG | TTGG | 40.83 |
| IRIS_313-8660 | - | TTGG | 60.73 |
| IRIS_313-8674 | TTATTATTATATTA | TTGG | 52.90 |
| IRIS_313-8699 | TTATTATTATATTA | TTGG | 58.40 |
| IRIS_313-8704 | CCGCCGGCGCCCCG | TTGG | 45.30 |
| IRIS_313-8717 | CCGCCGGCGCCCCG | TTGG | 21.73 |
| IRIS_313-8722 | - | GCAA | 36.77 |
| IRIS_313-8725 | CCGCCGGCGCCCCG | TTGG | 51.47 |
| IRIS_313-8727 | CCGCCGGCGCCCCG | TTGG | 46.23 |
| IRIS_313-8731 | CCGCCGGCGCCCCG | TTGG | 56.70 |
| IRIS_313-8732 | CCGCCGGCGCCCCG | TTGG | 42.73 |
| IRIS_313-8733 | CCGCCGGCGCCCCG | TTGG | 24.77 |
| IRIS_313-8737 | CCGCCGGCGCCCCG | TTGG | 29.67 |
| IRIS_313-8743 | CCGCCGGCGCCCCG | TTGG | 46.60 |
| IRIS_313-8744 | CCGCCGGCGCCCCG | TTGG | 29.47 |
| IRIS_313-8751 | TTATTATTATATTA | TTGG | 55.63 |
| IRIS_313-8754 | CCGCCGGCGCCCCG | TTGG | 39.13 |
| IRIS_313-8757 | CCGCCGGCGCCCCG | TTGG | 45.00 |
| IRIS_313-8781 | CCGCCGGCGCCCCG | TTGG | 47.23 |
| IRIS_313-8785 | TTATTATTATATTA | GCAA | 36.50 |

Table S1

| Accession IDs | chr8 (block1) | chr11 | Average % HRY |
|---|---|---|---|
| IRIS_313-8796 | CCGCCGGCGCCCCG | TTGG | 55.20 |
| IRIS_313-8812 | CCGCCGGCGCCCCG | TTGG | 54.53 |
| IRIS_313-8831 | CCGCCGGCGCCCCG | TTGG | 37.73 |
| IRIS_313-8844 | CCGCCGGCGCCCCG | GCAA | 37.23 |
| IRIS_313-8846 | CCGCCGGCGCCCCG | TTGG | 45.60 |
| IRIS_313-8850 | CCGCCGGCGCCCCG | TTGG | 28.70 |
| IRIS_313-8854 | CCGCCGGCGCCCCG | TTGG | 50.93 |
| IRIS_313-8889 | TTATTATTATATTA | TTGG | 60.73 |
| IRIS_313-8900 | **CCGTTATTATATTA** | TTGG | 54.07 |
| IRIS_313-8903 | CCGCCGGCGCCCCG | TTGG | 53.00 |
| IRIS_313-8914 | CCGCCGGCGCCCCG | TTGG | 40.43 |
| IRIS_313-8920 | - | TTGG | 38.50 |
| IRIS_313-8924 | **CCGTTATTATATTA** | TTGG | 48.37 |
| IRIS_313-8925 | CCGCCGGCGCCCCG | TTGG | 48.37 |
| IRIS_313-8930 | CCGCCGGCGCCCCG | TTGG | 32.63 |
| IRIS_313-8932 | CCGCCGGCGCCCCG | TTGG | 61.23 |
| IRIS_313-8935 | CCGCCGGCGCCCCG | TTGG | 48.93 |
| IRIS_313-8940 | CCGCCGGCGCCCCG | TTGG | 48.13 |
| IRIS_313-8946 | CCGCCGGCGCCCCG | TTGG | 58.77 |
| IRIS_313-8956 | CCGCCGGCGCCCCG | TTGG | 38.87 |
| IRIS_313-8967 | CCGCCGGCGCCCCG | TTGG | 56.50 |
| IRIS_313-8968 | CCGCCGGCGCCCCG | TTGG | 43.63 |
| IRIS_313-8980 | TTATTATTATATTA | GCAA | 39.07 |
| IRIS_313-8982 | CCGCCGGCGCCCCG | TTGG | 40.00 |
| IRIS_313-8986 | CCGCCGGCGCCCCG | TTGG | 32.87 |
| IRIS_313-8988 | CCGCCGGCGCCCCG | TTGG | 52.07 |
| IRIS_313-8994 | TTATTATTATATTA | TTGG | 53.47 |
| IRIS_313-9005 | CCGCCGGCGCCCCG | TTGG | 28.23 |
| IRIS_313-9017 | CCGCCGGCGCCCCG | TTGG | 29.87 |
| IRIS_313-9023 | CCGCCGGCGCCCCG | TTGG | 37.23 |
| IRIS_313-9032 | CCGCCGGCGCCCCG | TTGG | 42.20 |
| IRIS_313-9044 | CCGCCGGCGCCCCG | TTGG | 30.63 |
| IRIS_313-9065 | CCGCCGGCGCCCCG | TTGG | 42.90 |
| IRIS_313-9066 | CCGCCGGCGCCCCG | TTGG | 49.37 |
| IRIS_313-9067 | CCGCCGGCGCCCCG | TTGG | 22.63 |
| IRIS_313-9072 | **CCGTTATTATATTA** | TTGG | 47.40 |
| IRIS_313-9111 | - | TTGG | 55.37 |
| IRIS_313-9119 | TTATTATTATATTA | TTGG | 22.70 |
| IRIS_313-9139 | CCGCCGGCGCCCCG | TTGG | 44.23 |
| IRIS_313-9148 | CCGCCGGCGCCCCG | TTGG | 44.07 |
| IRIS_313-9156 | **CCGTTATTATATTA** | TTGG | 53.77 |
| IRIS_313-9174 | CCGCCGGCGCCCCG | TTGG | 46.53 |
| IRIS_313-9184 | CCGCCGGCGCCCCG | TTGG | 49.50 |
| IRIS_313-9190 | CCGCCGGCGCCCCG | TTGG | 54.20 |
| IRIS_313-9198 | - | TTGG | 56.83 |
| IRIS_313-9204 | CCGCCGGCGCCCCG | TTGG | 57.57 |
| IRIS_313-9218 | CCGCCGGCGCCCCG | TTGG | 31.53 |
| IRIS_313-9227 | CCGCCGGCGCCCCG | TTGG | 52.90 |
| IRIS_313-9253 | - | TTGG | 38.03 |
| IRIS_313-9256 | CCGCCGGCGCCCCG | TTGG | 28.47 |
| IRIS_313-9259 | CCGCCGGCGCCCCG | TTGG | 53.10 |
| IRIS_313-9262 | CCGCCGGCGCCCCG | TTGG | 50.93 |
| IRIS_313-9271 | CCGCCGGCGCCCCG | TTGG | 43.00 |
| IRIS_313-9281 | CCGCCGGCGCCCCG | TTGG | 46.90 |

Table S1

| Accession IDs | chr8 (block1) | chr11 | Average % HRY |
|---|---|---|---|
| IRIS_313-9286 | CCGCCGGCGCCCCG | TTGG | 26.30 |
| IRIS_313-9287 | CCGCCGGCGCCCCG | TTGG | 37.97 |
| IRIS_313-9313 | CCGCCGGCGCCCCG | TTGG | 42.13 |
| IRIS_313-9314 | CCGCCGGCGCCCCG | TTGG | 35.00 |
| IRIS_313-9317 | TTATTATTATATTA | TTGG | 55.70 |
| IRIS_313-9320 | CCGCCGGCGCCCCG | TTGG | 41.40 |
| IRIS_313-9324 | CCGCCGGCGCCCCG | TTGG | 23.50 |
| IRIS_313-9325 | CCGCCGGCGCCCCG | TTGG | 47.03 |
| IRIS_313-9348 | CCGCCGGCGCCCCG | TTGG | 44.17 |
| IRIS_313-9351 | CCGCCGGCGCCCCG | TTGG | 55.00 |
| IRIS_313-9360 | CCGCCGGCGCCCCG | TTGG | 50.70 |
| IRIS_313-9372 | CCGCCGGCGCCCCG | TTGG | 40.70 |
| IRIS_313-9384 | CCGCCGGCGCCCCG | TTGG | 51.37 |
| IRIS_313-9388 | CCGCCGGCGCCCCG | TTGG | 39.20 |
| IRIS_313-9400 | - | TTGG | 43.17 |
| IRIS_313-9403 | CCGCCGGCGCCCCG | TTGG | 44.77 |
| IRIS_313-9424 | TTATTATTATATTA | TTGG | 43.43 |
| IRIS_313-9427 | CCGCCGGCGCCCCG | TTGG | 58.77 |
| IRIS_313-9429 | - | TTGG | 58.30 |
| IRIS_313-9433 | CCGCCGGCGCCCCG | TTGG | 40.33 |
| IRIS_313-9451 | - | TTGG | 50.60 |
| IRIS_313-9461 | CCGCCGGCGCCCCG | GCAA | 42.33 |
| IRIS_313-9469 | CCGCCGGCGCCCCG | TTGG | 21.77 |
| IRIS_313-9472 | CCGCCGGCGCCCCG | TTGG | 47.10 |
| IRIS_313-9482 | CCGCCGGCGCCCCG | TTGG | 38.33 |
| IRIS_313-9484 | CCGCCGGCGCCCCG | TTGG | 16.93 |
| IRIS_313-9492 | CCGCCGGCGCCCCG | TTGG | 44.17 |
| IRIS_313-9503 | CCGCCGGCGCCCCG | TTGG | 27.10 |
| IRIS_313-9505 | CCGCCGGCGCCCCG | TTGG | 30.90 |
| IRIS_313-9522 | CCGCCGGCGCCCCG | TTGG | 39.47 |
| IRIS_313-9533 | TTATTATTATATTA | TTGG | 45.63 |
| IRIS_313-9547 | CCGCCGGCGCCCCG | TTGG | 55.40 |
| IRIS_313-9551 | CCGCCGGCGCCCCG | TTGG | 36.97 |
| IRIS_313-9557 | CCGCCGGCGCCCCG | TTGG | 36.37 |
| IRIS_313-9560 | CCGCCGGCGCCCCG | TTGG | 52.47 |
| IRIS_313-9566 | - | TTGG | 54.97 |
| IRIS_313-9570 | CCGCCGGCGCCCCG | TTGG | 44.57 |
| IRIS_313-9572 | CCGCCGGCGCCCCG | TTGG | 33.60 |
| IRIS_313-9574 | CCGCCGGCGCCCCG | TTGG | 27.93 |
| IRIS_313-9590 | CCGCCGGCGCCCCG | TTGG | 49.73 |
| IRIS_313-9593 | CCGCCGGCGCCCCG | TTGG | 59.90 |
| IRIS_313-9594 | CCGCCGGCGCCCCG | TTGG | 50.90 |
| IRIS_313-9602 | CCGCCGGCGCCCCG | TTGG | 48.93 |
| IRIS_313-9604 | CCGCCGGCGCCCCG | TTGG | 52.33 |
| IRIS_313-9605 | CCGCCGGCGCCCCG | TTGG | 51.77 |
| IRIS_313-9609 | - | TTGG | 41.40 |
| IRIS_313-9611 | TTATTATTATATTA | TTGG | 52.83 |
| IRIS_313-9617 | CCGCCGGCGCCCCG | TTGG | 21.97 |
| IRIS_313-9623 | CCGCCGGCGCCCCG | TTGG | 40.93 |
| IRIS_313-9634 | CCGCCGGCGCCCCG | TTGG | 42.27 |
| IRIS_313-9696 | - | TTGG | 57.33 |
| IRIS_313-9705 | CCGCCGGCGCCCCG | TTGG | 43.13 |
| IRIS_313-9706 | - | TTGG | 33.07 |
| IRIS_313-9708 | CCGCCGGCGCCCCG | TTGG | 29.97 |

Table S1

| Accession IDs | chr8 (block1) | chr11 | Average % HRY |
|---|---|---|---|
| IRIS_313-9723 | CCGCCGGCGCCCCG | TTGG | 47.27 |
| IRIS_313-9727 | CCGCCGGCGCCCCG | TTGG | 39.80 |
| IRIS_313-9730 | CCGCCGGCGCCCCG | TTGG | 54.97 |
| IRIS_313-9732 | CCGCCGGCGCCCCG | TTGG | 34.67 |
| IRIS_313-9740 | CCGCCGGCGCCCCG | TTGG | 32.97 |
| IRIS_313-9758 | CCGCCGGCGCCCCG | TTGG | 52.50 |
| IRIS_313-9767 | CCGCCGGCGCCCCG | TTGG | 54.73 |
| IRIS_313-9791 | CCGCCGGCGCCCCG | TTGG | 31.73 |
| IRIS_313-9795 | - | TTGG | 42.60 |
| IRIS_313-9831 | CCGCCGGCGCCCCG | GCAG | 34.03 |
| IRIS_313-9841 | CCGCCGGCGCCCCG | TTGG | 13.67 |
| IRIS_313-9862 | CCGCCGGCGCCCCG | TTGG | 25.53 |
| IRIS_313-9867 | CCGCCGGCGCCCCG | TTGG | 18.47 |
| IRIS_313-9882 | CCGCCGGCGCCCCG | **TTGA** | 39.87 |
| IRIS_313-9898 | - | GCAA | 20.20 |
| IRIS_313-9917 | CCGCCGGCGCCCCG | TTGG | 50.20 |
| IRIS_313-9922 | CCGCCGGCGCCCCG | TTGG | 50.50 |
| IRIS_313-9924 | - | TTGG | 40.73 |
| IRIS_313-9925 | CCGCCGGCGCCCCG | TTGG | 53.90 |
| IRIS_313-9940 | CCGCCGGCGCCCCG | TTGG | 45.57 |
| IRIS_313-9944 | CCGCCGGCGCCCCG | TTGG | 50.77 |
| IRIS_313-9953 | CCGCCGGCGCCCCG | TTGG | 51.60 |
| IRIS_313-9966 | CCGCCGGCGCCCCG | TTGG | 49.13 |

Table S2

**Table S2: Accessions used in the study, with the exiting haplotypes for HRY stability within candidate region on chromosome 8.**

Favourable haplotype identified for high HRY stability

- Missing/heterozygous

| Accession IDs | LOC_Os08g31340 | HRY Stability |
|---|---|---|
| IRIS_313-10000 | CTG | 1.00 |
| IRIS_313-10001 | CTG | 0.49 |
| IRIS_313-10007 | CTG | 0.69 |
| IRIS_313-10016 | CTG | 0.62 |
| IRIS_313-10026 | CTG | 0.77 |
| IRIS_313-10030 | CTG | 0.78 |
| IRIS_313-10034 | CTG | 0.94 |
| IRIS_313-10040 | CTG | 1.00 |
| IRIS_313-10045 | CTG | 0.98 |
| IRIS_313-10046 | CTG | 0.67 |
| IRIS_313-10047 | CTG | 0.76 |
| IRIS_313-10048 | CTG | 0.96 |
| IRIS_313-10050 | CTG | 0.77 |
| IRIS_313-10054 | CTG | 0.42 |
| IRIS_313-10102 | CTG | 0.91 |
| IRIS_313-10103 | CTG | 1.53 |
| IRIS_313-10109 | CTG | 0.54 |
| IRIS_313-10113 | CTG | 0.90 |
| IRIS_313-10114 | CTG | 0.57 |
| IRIS_313-10129 | CTG | 0.88 |
| IRIS_313-10147 | CTG | 0.99 |
| IRIS_313-10148 | CTG | 0.62 |
| IRIS_313-10151 | CTG | 0.89 |
| IRIS_313-10154 | - | 0.79 |
| IRIS_313-10167 | CTG | 0.68 |
| IRIS_313-10168 | CTG | 0.82 |
| IRIS_313-10170 | CTG | 0.92 |
| IRIS_313-10171 | CTG | 0.68 |
| IRIS_313-10177 | CTG | 0.67 |
| IRIS_313-10178 | CTG | 0.80 |
| IRIS_313-10179 | CTG | 0.78 |
| IRIS_313-10191 | CTG | 1.06 |
| IRIS_313-10220 | CTG | 0.59 |
| IRIS_313-10221 | CTG | 0.99 |
| IRIS_313-10224 | CTG | 0.76 |
| IRIS_313-10226 | CTC | 0.82 |
| IRIS_313-10234 | CTG | 0.30 |
| IRIS_313-10235 | CTG | 0.64 |
| IRIS_313-10237 | CTG | 0.35 |
| IRIS_313-10238 | CTG | 0.63 |
| IRIS_313-10247 | CTG | 0.34 |
| IRIS_313-10257 | CTG | 0.93 |
| IRIS_313-10260 | CTG | 0.63 |
| IRIS_313-10263 | CTG | 0.45 |
| IRIS_313-10268 | CTG | 0.58 |
| IRIS_313-10271 | CTG | 0.52 |
| IRIS_313-10274 | CTG | 0.83 |

Table S2

| Accession IDs | LOC_Os08g31340 | HRY Stability |
|---|---|---|
| IRIS_313-10275 | CTG | 0.53 |
| IRIS_313-10279 | CTG | 0.33 |
| IRIS_313-10285 | CTG | 0.53 |
| IRIS_313-10287 | CTG | 0.83 |
| IRIS_313-10289 | CTG | 0.62 |
| IRIS_313-10293 | CTG | 0.67 |
| IRIS_313-10294 | CTG | 0.57 |
| IRIS_313-10298 | CTG | 0.49 |
| IRIS_313-10300 | CTC | 0.72 |
| IRIS_313-10301 | CTG | 0.74 |
| IRIS_313-10307 | CTG | 0.71 |
| IRIS_313-10314 | CTG | 0.41 |
| IRIS_313-10318 | CTG | 0.61 |
| IRIS_313-10325 | CTG | 0.89 |
| IRIS_313-10332 | CTG | 0.80 |
| IRIS_313-10333 | CTG | 0.65 |
| IRIS_313-10334 | CTG | 0.81 |
| IRIS_313-10337 | CTG | 0.66 |
| IRIS_313-10340 | CTG | 0.54 |
| IRIS_313-10349 | CTG | 0.54 |
| IRIS_313-10352 | CTG | 0.52 |
| IRIS_313-10353 | CTG | 0.38 |
| IRIS_313-10355 | **TCC** | 0.82 |
| IRIS_313-10357 | CTG | 0.61 |
| IRIS_313-10360 | CTG | 0.87 |
| IRIS_313-10361 | CTG | 0.74 |
| IRIS_313-10366 | CTG | 0.60 |
| IRIS_313-10371 | CTG | 0.51 |
| IRIS_313-10374 | CTG | 0.82 |
| IRIS_313-10375 | CTG | 0.47 |
| IRIS_313-10385 | CTG | 1.05 |
| IRIS_313-10392 | CTG | 1.08 |
| IRIS_313-10394 | CTG | 0.61 |
| IRIS_313-10396 | CTG | 0.78 |
| IRIS_313-10397 | CTG | 0.84 |
| IRIS_313-10398 | CTG | 0.87 |
| IRIS_313-10399 | CTG | 0.92 |
| IRIS_313-10400 | CTG | 0.69 |
| IRIS_313-10401 | CTG | 0.75 |
| IRIS_313-10402 | CTG | 0.88 |
| IRIS_313-10403 | CTG | 0.71 |
| IRIS_313-10404 | CTG | 0.51 |
| IRIS_313-10412 | CTG | 0.64 |
| IRIS_313-10417 | CTG | 0.44 |
| IRIS_313-10421 | CTG | 0.53 |
| IRIS_313-10422 | CTG | 0.87 |
| IRIS_313-10423 | CTG | 0.53 |
| IRIS_313-8288 | CTG | 0.44 |
| IRIS_313-8303 | CTG | 0.61 |
| IRIS_313-8305 | CTG | 0.80 |
| IRIS_313-8315 | CTG | 0.46 |
| IRIS_313-8332 | CTG | 1.01 |
| IRIS_313-8368 | CTG | 0.59 |

Table S2

| Accession IDs | LOC_Os08g31340 | HRY Stability |
|---|---|---|
| IRIS_313-8380 | CTG | 0.72 |
| IRIS_313-8386 | CTG | 0.72 |
| IRIS_313-8392 | CTG | 0.71 |
| IRIS_313-8401 | CTG | 0.73 |
| IRIS_313-8409 | CTG | 0.94 |
| IRIS_313-8412 | CTG | 0.64 |
| IRIS_313-8414 | CTG | 0.79 |
| IRIS_313-8433 | CTG | 0.99 |
| IRIS_313-8435 | CTG | 0.74 |
| IRIS_313-8437 | CTG | 0.90 |
| IRIS_313-8450 | CTG | 0.81 |
| IRIS_313-8453 | **TCC** | 0.99 |
| IRIS_313-8454 | CTG | 0.85 |
| IRIS_313-8458 | CTG | 0.58 |
| IRIS_313-8509 | CTG | 0.85 |
| IRIS_313-8514 | CTG | 0.59 |
| IRIS_313-8530 | CTG | 0.88 |
| IRIS_313-8536 | CTG | 0.81 |
| IRIS_313-8559 | CTG | 0.83 |
| IRIS_313-8568 | CTG | 0.94 |
| IRIS_313-8571 | CTG | 0.82 |
| IRIS_313-8585 | CTG | 0.74 |
| IRIS_313-8586 | CTG | 0.38 |
| IRIS_313-8595 | CTG | 1.09 |
| IRIS_313-8603 | CTG | 0.49 |
| IRIS_313-8614 | CTG | 1.04 |
| IRIS_313-8616 | CTG | 0.54 |
| IRIS_313-8621 | CTG | 0.57 |
| IRIS_313-8622 | CTG | 0.73 |
| IRIS_313-8631 | CTG | 0.74 |
| IRIS_313-8632 | CTG | 1.04 |
| IRIS_313-8638 | CTG | 0.60 |
| IRIS_313-8643 | CTG | 0.95 |
| IRIS_313-8645 | CTG | 1.11 |
| IRIS_313-8647 | CTG | 0.91 |
| IRIS_313-8659 | CTG | 1.00 |
| IRIS_313-8660 | CTG | 0.94 |
| IRIS_313-8674 | CTG | 0.79 |
| IRIS_313-8699 | CTG | 0.96 |
| IRIS_313-8704 | CTG | 0.69 |
| IRIS_313-8717 | **TCC** | 1.16 |
| IRIS_313-8722 | CTG | 0.10 |
| IRIS_313-8725 | CTG | 0.97 |
| IRIS_313-8727 | CTG | 0.55 |
| IRIS_313-8731 | CTG | 0.65 |
| IRIS_313-8732 | CTG | 0.83 |
| IRIS_313-8733 | **TCC** | 1.18 |
| IRIS_313-8737 | **TCC** | 1.29 |
| IRIS_313-8743 | CTG | 0.89 |
| IRIS_313-8744 | CTG | 1.13 |
| IRIS_313-8751 | CTG | 0.56 |
| IRIS_313-8754 | CTG | 0.75 |
| IRIS_313-8757 | CTG | 0.75 |

Table S2

| Accession IDs | LOC_Os08g31340 | HRY Stability |
|---|---|---|
| IRIS_313-8781 | CTG | 0.92 |
| IRIS_313-8785 | **TCC** | 0.98 |
| IRIS_313-8796 | CTG | 0.74 |
| IRIS_313-8812 | CTG | 0.89 |
| IRIS_313-8831 | CTG | 1.10 |
| IRIS_313-8844 | **TCC** | 0.98 |
| IRIS_313-8846 | CTG | 0.86 |
| IRIS_313-8850 | CTG | 1.10 |
| IRIS_313-8854 | CTG | 0.78 |
| IRIS_313-8889 | CTG | 0.88 |
| IRIS_313-8900 | CTG | 0.92 |
| IRIS_313-8903 | CTG | 0.74 |
| IRIS_313-8914 | CTG | 0.84 |
| IRIS_313-8920 | **TCC** | 1.18 |
| IRIS_313-8924 | CTG | 0.74 |
| IRIS_313-8925 | **TCC** | 1.04 |
| IRIS_313-8930 | CTG | 0.63 |
| IRIS_313-8932 | CTG | 0.84 |
| IRIS_313-8935 | CTG | 0.82 |
| IRIS_313-8940 | CTG | 0.96 |
| IRIS_313-8946 | CTG | 0.76 |
| IRIS_313-8956 | CTG | 1.24 |
| IRIS_313-8967 | CTG | 0.68 |
| IRIS_313-8968 | CTG | 0.99 |
| IRIS_313-8980 | CTG | 0.43 |
| IRIS_313-8982 | CTG | 0.84 |
| IRIS_313-8986 | CTG | 0.78 |
| IRIS_313-8988 | CTG | 0.58 |
| IRIS_313-8994 | CTG | 0.80 |
| IRIS_313-9005 | CTG | 0.62 |
| IRIS_313-9017 | CTG | 0.55 |
| IRIS_313-9023 | CTG | 0.52 |
| IRIS_313-9032 | - | 0.83 |
| IRIS_313-9044 | CTG | 0.71 |
| IRIS_313-9065 | - | 1.09 |
| IRIS_313-9066 | CTG | 0.99 |
| IRIS_313-9067 | **TCC** | 0.96 |
| IRIS_313-9072 | CTG | 0.88 |
| IRIS_313-9111 | CTG | 0.97 |
| IRIS_313-9119 | CTG | 0.91 |
| IRIS_313-9139 | CTG | 0.91 |
| IRIS_313-9148 | CTG | 0.73 |
| IRIS_313-9156 | CTG | 0.78 |
| IRIS_313-9174 | CTG | 0.93 |
| IRIS_313-9184 | CTG | 0.90 |
| IRIS_313-9190 | **TCC** | 0.73 |
| IRIS_313-9198 | **TCC** | 0.87 |
| IRIS_313-9204 | CTG | 0.91 |
| IRIS_313-9218 | **TCC** | 0.95 |
| IRIS_313-9227 | CTG | 0.49 |
| IRIS_313-9253 | CTG | 0.76 |
| IRIS_313-9256 | CTG | 0.67 |
| IRIS_313-9259 | CTC | 0.70 |

Table S2

| Accession IDs | LOC_Os08g31340 | HRY Stability |
|---|---|---|
| IRIS_313-9262 | CTG | 0.56 |
| IRIS_313-9271 | CTG | 0.71 |
| IRIS_313-9281 | CTG | 0.70 |
| IRIS_313-9286 | CTG | 0.75 |
| IRIS_313-9287 | CTG | 0.55 |
| IRIS_313-9313 | CTG | 0.71 |
| IRIS_313-9314 | CTG | 0.90 |
| IRIS_313-9317 | CTG | 0.94 |
| IRIS_313-9320 | CTG | 0.70 |
| IRIS_313-9324 | CTG | 0.77 |
| IRIS_313-9325 | CTG | 0.61 |
| IRIS_313-9348 | CTG | 0.66 |
| IRIS_313-9351 | CTG | 0.68 |
| IRIS_313-9360 | **TCC** | 0.78 |
| IRIS_313-9372 | CTG | 0.73 |
| IRIS_313-9384 | CTG | 1.11 |
| IRIS_313-9388 | CTG | 0.90 |
| IRIS_313-9400 | CTG | 0.56 |
| IRIS_313-9403 | **TCC** | 0.99 |
| IRIS_313-9424 | CTG | 0.88 |
| IRIS_313-9427 | CTG | 0.69 |
| IRIS_313-9429 | CTG | 0.90 |
| IRIS_313-9433 | CTG | 0.86 |
| IRIS_313-9451 | CTG | 0.84 |
| IRIS_313-9461 | CTG | 0.92 |
| IRIS_313-9469 | CTG | 0.85 |
| IRIS_313-9472 | CTG | 0.58 |
| IRIS_313-9482 | CTG | 0.62 |
| IRIS_313-9484 | CTG | 0.39 |
| IRIS_313-9492 | CTG | 0.75 |
| IRIS_313-9503 | - | 0.45 |
| IRIS_313-9505 | CTG | 0.77 |
| IRIS_313-9522 | CTG | 0.85 |
| IRIS_313-9533 | CTG | 0.73 |
| IRIS_313-9547 | CTG | 0.92 |
| IRIS_313-9551 | CTG | 0.90 |
| IRIS_313-9557 | CTG | 1.15 |
| IRIS_313-9560 | CTG | 0.68 |
| IRIS_313-9566 | CTG | 0.76 |
| IRIS_313-9570 | CTG | 0.83 |
| IRIS_313-9572 | CTG | 0.91 |
| IRIS_313-9574 | **TCC** | 1.40 |
| IRIS_313-9590 | CTG | 0.98 |
| IRIS_313-9593 | CTG | 0.70 |
| IRIS_313-9594 | CTG | 0.88 |
| IRIS_313-9602 | CTG | 0.85 |
| IRIS_313-9604 | CTG | 0.79 |
| IRIS_313-9605 | CTG | 0.89 |
| IRIS_313-9609 | **TCC** | 0.99 |
| IRIS_313-9611 | CTG | 0.75 |
| IRIS_313-9617 | **TCC** | 1.30 |
| IRIS_313-9623 | CTG | 0.72 |
| IRIS_313-9634 | CTC | 0.39 |

Table S2

| Accession IDs | LOC_Os08g31340 | HRY Stability |
|---|---|---|
| IRIS_313-9696 | CTG | 0.95 |
| IRIS_313-9705 | CTG | 0.87 |
| IRIS_313-9706 | CTG | 0.90 |
| IRIS_313-9708 | CTG | 0.48 |
| IRIS_313-9723 | CTG | 1.03 |
| IRIS_313-9727 | CTG | 0.91 |
| IRIS_313-9730 | CTG | 0.70 |
| IRIS_313-9732 | CTG | 0.65 |
| IRIS_313-9740 | CTG | 0.60 |
| IRIS_313-9758 | CTG | 0.93 |
| IRIS_313-9767 | CTG | 0.89 |
| IRIS_313-9791 | CTG | 0.55 |
| IRIS_313-9795 | CTG | 0.61 |
| IRIS_313-9831 | CTG | 1.12 |
| IRIS_313-9841 | CTG | 0.72 |
| IRIS_313-9862 | CTG | 1.13 |
| IRIS_313-9867 | CTG | 0.83 |
| IRIS_313-9882 | CTG | 0.81 |
| IRIS_313-9898 | - | 0.46 |
| IRIS_313-9917 | CTG | 0.86 |
| IRIS_313-9922 | CTG | 0.85 |
| IRIS_313-9924 | CTG | 0.70 |
| IRIS_313-9925 | CTG | 0.81 |
| IRIS_313-9940 | CTG | 0.49 |
| IRIS_313-9944 | CTG | 0.85 |
| IRIS_313-9953 | CTG | 0.50 |
| IRIS_313-9966 | CTG | 0.30 |

# Table S3

**Table S3: Single locus GWAS for SNPs detected against Nipponbare reference genome with corrosponding orthologous IDs In ZS97 and MH63.**

| Position | Chr | Ref | Alt | Beta | -log10 p-value | Region | Gene | Function of the Gene | Orthologous_ZS97 | Orthologoues_MH63 |
|---|---|---|---|---|---|---|---|---|---|---|
| 5117290 | 5 | T | C | 0.29 | 4.95 | Upstream | LOC_Os05g09160 | Unknown | Not Present | MH05g0088700 |
| 5237830 | 5 | A | G | 0.35 | 7.14 | Upstream | LOC_Os05g09360 | Unknown | ZS05g0108900 | MH05g0091600 |
| 5299051 | 5 | A | G | 0.30 | 5.43 | Upstream | LOC_Os05g09450 | Unknown | Not present | Not present |
| 5357676 | 5 | C | A | -0.44 | 11.81 | Upstream | LOC_Os05g09510 | Unknown | ZS05g0109900 | MH05g0092800 |
| 5358236 | 5 | G | C | -0.42 | 9.89 | Intergenic | LOC_Os05g09510, LOC_Os05g09520 (GW5) | Unknown, Signalling calcium | ZS05g0109900, ZS05g0110000 | MH05g0092800, MH05g0093000 |
| 5359283 | 5 | G | A | -0.39 | 10.26 | Intergenic | LOC_Os05g09510, LOC_Os05g09520 (GW5) | Unknown, Signalling calcium | ZS05g0109900, ZS05g0110000 | MH05g0092800, MH05g0093000 |
| 5359520 | 5 | G | A | -0.48 | 14.45 | Intergenic | LOC_Os05g09510, LOC_Os05g09520 | Unknown, Signalling calcium | ZS05g0109900, ZS05g0110000 | MH05g0092800, MH05g0093000 |
| 5361195 | 5 | A | T | -0.37 | 9.83 | Upstream | LOC_Os05g09520 | Signalling calcium | ZS05g0110000 | MH05g0093000 |
| 5361276 | 5 | T | C | -0.49 | 15.04 | Upstream | LOC_Os05g09520 | Signalling calcium | ZS05g0110000 | MH05g0093000 |
| 5363611 | 5 | A | G | -0.42 | 12.26 | Upstream | LOC_Os05g09520 | Signalling calcium | ZS05g0110000 | MH05g0093000 |
| 5368403 | 5 | A | G | -0.34 | 8.61 | Downstream | LOC_Os05g09520 | Signalling calcium | ZS05g0110000 | MH05g0093000 |
| 5369802 | 5 | A | G | -0.42 | 11.82 | Downstream | LOC_Os05g09520 | Signalling calcium | ZS05g0110000 | MH05g0093000 |
| 5373379 | 5 | G | A | -0.38 | 10.21 | Intergenic | LOC_Os05g09520, LOC_Os05g09530 | Signalling calcium, Aspartate protease | ZS05g0110000-ZS05g0110100 | MH05g0093000, MH05g0093100 |
| 5374961 | 5 | C | T | -0.34 | 8.19 | Intergenic | LOC_Os05g09520, LOC_Os05g09530 | Signalling calcium, Aspartate protease | ZS05g0110000-ZS05g0110100 | MH05g0093000, MH05g0093100 |
| 5377012 | 5 | A | G | -0.28 | 5.73 | Intergenic | LOC_Os05g09520, LOC_Os05g09530 | Signalling calcium, Aspartate protease | ZS05g0110000-ZS05g0110100 | MH05g0093000, MH05g0093100 |
| 5377744 | 5 | T | A | -0.27 | 5.55 | Intergenic | LOC_Os05g09520, LOC_Os05g09530 | Signalling calcium, Aspartate protease | ZS05g0110000-ZS05g0110100 | MH05g0093000, MH05g0093100 |
| 5378981 | 5 | A | T | -0.37 | 9.01 | Downstream | LOC_Os05g09520, LOC_Os05g09530 | Signalling calcium, Aspartate protease | ZS05g0110000-ZS05g0110100 | MH05g0093000, MH05g0093100 |
| 5433386 | 5 | T | C | 0.30 | 6.91 | Downstream | LOC_Os05g09620 | Unknown | ZS05g0110700 | MH05g0094000 |

Table S4

**Table S4 : Accessions used in the study, with the exiting haplotypes for lowering chalk in hotspot regions of chromosome 5.**

Favourable haplotype identified for lowering chalk (green)

- Missing/heterozygous (grey)

| Accession IDs | New_gene | LOC_Os05g09520 | LOC_Os05g09530 | PGC |
|---|---|---|---|---|
| IRIS_313-10000 | ATATGCTAAGAGCTA | GCTAGG | TGGTTCTGTATTCGTTGGAA | 60.30 |
| IRIS_313-10001 | GCTCAAAGGAGATCG | ATCGTT | TGGGTCTGTATTCGTTGGAA | 3.95 |
| IRIS_313-10007 | ATATGCTAAGAGCTA | GCTAGG | TGGTTCTGTATTCGTTGGAA | 92.75 |
| IRIS_313-10016 | GCTCAAAGGAGATCG | ATCGTT | TGGGTCTGTATTCGTTGGAA | 7.18 |
| IRIS_313-10026 | - | - | **TGGGTCTGTGTTCGTTGGAA** | 30.98 |
| IRIS_313-10030 | ATATGCTAAGAGCTA | GCTAGG | CAAGCTCACGACAAACTAGG | 84.87 |
| IRIS_313-10034 | ATATGCTAAGAGCTA | - | TGGGTCTGTATTCGTTGGAA | 2.73 |
| IRIS_313-10040 | ATATGCTAAGAGCTA | GCTAGG | CAAGCTCACGACAAACTAGG | 2.78 |
| IRIS_313-10045 | GCTCAAAGGAGATCG | ATCGTT | TGGGTCTGTATTCGTTGGAA | 7.28 |
| IRIS_313-10046 | GCTCAAAGGAGATCG | ATCGTT | TGGGTCTGTATTCGTTGGAA | 4.47 |
| IRIS_313-10047 | - | GCTAGG | CAAGCTCACGACAAACTAGG | 10.03 |
| IRIS_313-10048 | GCTCAAAGGAGATCG | ATCGTT | TGGGTCTGTATTCGTTGGAA | 9.29 |
| IRIS_313-10050 | ATATGCTAAGAGCTA | GCTAGG | - | 16.56 |
| IRIS_313-10054 | GCTCAAAGGAGATCG | ATCGTT | TGGGTCTGTATTCGTTGGAA | 4.61 |
| IRIS_313-10077 | GCTCAAAGGAGATCG | ATCGTT | TGGGTCTGTATTCGTTGGAA | 7.99 |
| IRIS_313-10102 | **GCTCGCTGGGGGCCG** | **GCCGGT** | **TGGGTCTGTGTTCGTTGGAA** | 7.40 |
| IRIS_313-10103 | GCTCAAAGGAGATCG | ATCGTT | TGGGTCTGTATTCGTTGGAA | 68.30 |
| IRIS_313-10109 | GCTCAAAGGAGATCG | ATCGTT | TGGGTCTGTATTCGTTGGAA | 8.38 |
| IRIS_313-10113 | - | ATCGTT | TGGGTCTGTATTCGTTGGAA | 3.02 |
| IRIS_313-10114 | GCTCAAAGGAGATCG | ATCGTT | TGGGTCTGTATTCGTTGGAA | 42.66 |
| IRIS_313-10129 | ATATGCTAAGAGCTA | GCTAGG | CAAGCTCACGACAAACTAGG | 30.79 |
| IRIS_313-10147 | - | GCTAGG | TGGTTCTGTATTCGTTGGAA | 1.38 |
| IRIS_313-10148 | ATATGCTAAGAGCTA | GCTAGG | CAAGCTCACGACAAACTAGG | 6.99 |
| IRIS_313-10151 | GCTCAAAGAGAGCTA | GCTAGG | TGGTTCTGTATTCGTTGGAA | 0.89 |
| IRIS_313-10154 | ATATGCTAAGAGCTA | GCTAGG | TGGTTCTGTATTCGTTGGAA | 28.10 |
| IRIS_313-10167 | GCTCAAAGGAGATCG | ATCGTT | TGGGTCTGTATTCGTTGGAA | 2.36 |
| IRIS_313-10168 | ATATGCTAAGAGCTA | GCTAGG | TGGTTCTGTATTCGTTGGAA | 58.20 |
| IRIS_313-10170 | - | - | TGGTTCTGTATTCGTTGGAA | 39.75 |
| IRIS_313-10171 | ATATGCTAAGAGCTA | GCTAGG | TGGTTCTGTATTCGTTGGAA | 35.57 |
| IRIS_313-10177 | ATATGCTAAGAGCTA | GCTAGG | CAAGCTCACGACAAACTAGG | 12.94 |
| IRIS_313-10178 | ATATGCTAAGAGCTA | GCTAGG | - | 7.70 |
| IRIS_313-10179 | ATATGCTAAGAGCTA | GCTAGG | TGGGTCTGTATTCGTTGGAA | 9.54 |
| IRIS_313-10191 | GCTCAAAGGAGATCG | ATCGTT | TGGGTCTGTATTCGTTGGAA | 0.17 |
| IRIS_313-10220 | **GCTCGCTGGGGGCCG** | **GCCGGT** | **TGGGTCTGTGTTCGTTGGAA** | 4.36 |
| IRIS_313-10221 | ATATGCTAAGAGCTA | GCTAGG | TGGGTCTGTATTCGTTGGAA | 9.33 |
| IRIS_313-10224 | - | - | **TGGGTCTGTGTTCGTTGGAA** | 21.80 |
| IRIS_313-10226 | ATATGCTAAGAGCTA | GCTAGG | - | 42.54 |
| IRIS_313-10234 | ATATGCTAAGAGCTA | GCTAGG | TGGTTCTGTATTCGTTGGAA | 21.53 |
| IRIS_313-10235 | GCTCAAAGGAGATCG | ATCGTT | TGGGTCTGTATTCGTTGGAA | 4.72 |
| IRIS_313-10237 | GCTCAAAGGAGATCG | ATCGTT | TGGGTCTGTATTCGTTGGAA | 2.55 |
| IRIS_313-10238 | GCTCAAAGGAGATCG | ATCGTT | TGGGTCTGTATTCGTTGGAA | 0.94 |
| IRIS_313-10247 | GCTCAAAGGAGATCG | ATCGTT | TGGGTCTGTATTCGTTGGAA | 0.87 |
| IRIS_313-10257 | GCTCAAAGGAGATCG | ATCGTT | - | 6.49 |
| IRIS_313-10260 | **GCTCGCTGGGGGCCG** | **GCCGGT** | **TGGGTCTGTGTTCGTTGGAA** | 6.47 |
| IRIS_313-10263 | - | **GCCGGT** | - | 2.61 |
| IRIS_313-10268 | - | **GCCGGT** | **TGGGTCTGTGTTCGTTGGAA** | 5.86 |
| IRIS_313-10271 | **GCTCGCTGGGGGCCG** | **GCCGGT** | - | 7.45 |
| IRIS_313-10274 | - | - | - | 1.22 |

Table S4

| Accession IDs | New_gene | LOC_Os05g09520 | LOC_Os05g09530 | PGC |
|---|---|---|---|---|
| IRIS_313-10275 | **GCTCGCTGGGGGCCG** | **GCCGGT** | **TGGGTCTGTGTTCGTTGGAA** | 8.67 |
| IRIS_313-10279 | GCTCAAAGAGAGCTA | GCTAGG | TGGTTCTGTATTCGTTGGAA | 6.67 |
| IRIS_313-10285 | GCTCAAAGGAGATCG | ATCGTT | TGGGTCTGTATTCGTTGGAA | 4.59 |
| IRIS_313-10287 | GCTCAAAGGAGATCG | ATCGTT | TGGGTCTGTATTCGTTGGAA | 4.75 |
| IRIS_313-10289 | GCTCAAAGGAGATCG | ATCGTT | TGGGTCTGTATTCGTTGGAA | 3.95 |
| IRIS_313-10293 | GCTCAAAGGAGATCG | ATCGTT | TGGGTCTGTATTCGTTGGAA | 0.91 |
| IRIS_313-10294 | GCTCAAAGGAGATCG | ATCGTT | TGGGTCTGTATTCGTTGGAA | 1.15 |
| IRIS_313-10298 | GCTCAAAGGAGATCG | ATCGTT | TGGGTCTGTATTCGTTGGAA | 12.73 |
| IRIS_313-10300 | GCTCAAAGGAGATCG | ATCGTT | TGGGTCTGTATTCGTTGGAA | 2.69 |
| IRIS_313-10301 | GCTCAAAGGAGATCG | ATCGTT | TGGGTCTGTATTCGTTGGAA | 11.13 |
| IRIS_313-10307 | GCTCAAAGGAGATCG | ATCGTT | TGGGTCTGTATTCGTTGGAA | 6.77 |
| IRIS_313-10314 | - | **GCCGGT** | **TGGGTCTGTGTTCGTTGGAA** | 2.25 |
| IRIS_313-10318 | GCTCAAAGGAGATCG | ATCGTT | TGGGTCTGTATTCGTTGGAA | 3.80 |
| IRIS_313-10325 | **GCTCGCTGGGGGCCG** | **GCCGGT** | **TGGGTCTGTGTTCGTTGGAA** | 3.40 |
| IRIS_313-10332 | GCTCAAAGGAGATCG | ATCGTT | TGGGTCTGTATTCGTTGGAA | 1.83 |
| IRIS_313-10333 | ATATGCTAAGAGCTA | GCTAGG | TGGTTCTGTATTCGTTGGAA | 22.76 |
| IRIS_313-10334 | ATATGCTAAGAGCTA | GCTAGG | - | 13.75 |
| IRIS_313-10337 | ATATGCTAAGAGCTA | GCTAGG | TGGGTCTGTATTCGTTGGAA | 42.29 |
| IRIS_313-10340 | GCTCAAAGGAGATCG | ATCGTT | TGGGTCTGTATTCGTTGGAA | 10.84 |
| IRIS_313-10349 | GCTCAAAGGAGATCG | ATCGTT | TGGGTCTGTATTCGTTGGAA | 7.61 |
| IRIS_313-10352 | **GCTCGCTGGGGGCCG** | **GCCGGT** | **TGGGTCTGTGTTCGTTGGAA** | 4.86 |
| IRIS_313-10353 | ATATGCTAAGAGCTA | GCTAGG | CAAGCTCACGACAAACTAGG | 92.38 |
| IRIS_313-10355 | GCTCAAAGGAGATCG | ATCGTT | TGGGTCTGTATTCGTTGGAA | 9.22 |
| IRIS_313-10357 | GCTCAAAGGAGATCG | ATCGTT | TGGGTCTGTATTCGTTGGAA | 3.54 |
| IRIS_313-10359 | - | - | **TGGGTCTGTGTTCGTTGGAA** | 16.02 |
| IRIS_313-10360 | GCTCAAAGGAGATCG | ATCGTT | TGGGTCTGTATTCGTTGGAA | 1.54 |
| IRIS_313-10361 | ATATGCTAAGAGCTA | GCTAGG | CAAGCTCACGACAAACTAGG | 30.73 |
| IRIS_313-10366 | - | - | TGGGTCTGTATTCGTTGGAA | 1.23 |
| IRIS_313-10371 | GCTCAAAGGAGATCG | ATCGTT | TGGGTCTGTATTCGTTGGAA | 5.48 |
| IRIS_313-10374 | GCTCAAAGGAGATCG | ATCGTT | TGGGTCTGTATTCGTTGGAA | 4.75 |
| IRIS_313-10375 | ATATGCTAAGAGCTA | GCTAGG | CAAGCCCACGACAAACTAGG | 29.99 |
| IRIS_313-10385 | - | ATCGTT | TGGGTCTGTATTCGTTGGAA | 1.33 |
| IRIS_313-10392 | GCTCAAAGGAGATCG | ATCGTT | TGGGTCTGTATTCGTTGGAA | 3.83 |
| IRIS_313-10394 | GCTCAAAGGAGATCG | ATCGTT | TGGGTCTGTATTCGTTGGAA | 9.40 |
| IRIS_313-10396 | GCTCAAAGGAGATCG | ATCGTT | TGGGTCTGTATTCGTTGGAA | 2.38 |
| IRIS_313-10397 | GCTCAAAGGAGATCG | ATCGTT | TGGGTCTGTATTCGTTGGAA | 0.43 |
| IRIS_313-10398 | GCTCAAAGGAGATCG | ATCGTT | TGGGTCTGTATTCGTTGGAA | 0.83 |
| IRIS_313-10399 | - | GCTAGG | TGGTTCTGTATTCGTTGGAA | 3.88 |
| IRIS_313-10400 | GCTCAAAGGAGATCG | ATCGTT | TGGGTCTGTATTCGTTGGAA | 0.61 |
| IRIS_313-10401 | - | ATCGTT | TGGGTCTGTATTCGTTGGAA | 2.97 |
| IRIS_313-10402 | GCTCAAAGGAGATCG | ATCGTT | TGGGTCTGTATTCGTTGGAA | 3.03 |
| IRIS_313-10403 | GCTCAAAGGAGATCG | ATCGTT | TGGGTCTGTATTCGTTGGAA | 0.14 |
| IRIS_313-10404 | ATATGCTAAGAGCTA | - | **TGGGTCTGTGTTCGTTGGAA** | 9.65 |
| IRIS_313-10412 | - | - | TGGTTCTGTATTCGTTGGAA | 15.54 |
| IRIS_313-10417 | - | GCTAGG | CAAGCTCACGACAAACTAGG | 26.98 |
| IRIS_313-10421 | ATATGCTAAGAGCTA | GCTAGG | TGGGTCTGTATTCGTTGGAA | 10.29 |
| IRIS_313-10422 | - | - | - | 22.84 |
| IRIS_313-10423 | ATATGCTAAGAGCTA | GCTAGG | CAAGCTCACGACAAACTAGG | 26.69 |
| IRIS_313-11437 | GCTCAAAGGAGATCG | ATCGTT | TGGGTCTGTATTCGTTGGAA | 5.01 |
| IRIS_313-11467 | GCTCAAAGGAGATCG | ATCGTT | TGGGTCTGTATTCGTTGGAA | 1.17 |
| IRIS_313-11478 | GCTCAAAGGAGATCG | ATCGTT | TGGGTCTGTATTCGTTGGAA | 18.58 |
| IRIS_313-11487 | - | - | TGGGTCTGTATTCGTTGGAA | 4.40 |
| IRIS_313-11508 | GCTCAAAGGAGATCG | ATCGTT | TGGGTCTGTATTCGTTGGAA | 1.02 |
| IRIS_313-11570 | - | GCTAGG | TGGTTCTGTATTCGTTGGAA | 50.43 |

Table S4

| Accession IDs | New_gene | LOC_Os05g09520 | LOC_Os05g09530 | PGC |
|---|---|---|---|---|
| IRIS_313-11573 | - | ATCGTT | TGGGTCTGTATTCGTTGGAA | 22.91 |
| IRIS_313-11577 | - | ATCGTT | TGGGTCTGTATTCGTTGGAA | 19.46 |
| IRIS_313-11580 | GCTCAAAGGAGATCG | ATCGTT | TGGGTCTGTATTCGTTGGAA | 0.08 |
| IRIS_313-11585 | ATTTGCTAAGAGCTA | GCTAGG | CAAGCTCACGACAAACTAGG | 25.96 |
| IRIS_313-11609 | GCTCAAAGGAGATCG | - | TGGGTCTGTATTCGTTGGAA | 9.21 |
| IRIS_313-11650 | GCTCAAAGGAGATCG | ATCGTT | TGGGTCTGTATTCGTTGGAA | 5.33 |
| IRIS_313-11671 | GCTCAAAGGAGATCG | ATCGTT | TGGGTCTGTATTCGTTGGAA | 4.68 |
| IRIS_313-11724 | ATATGCTAAGAGCTA | GCTAGG | CAAGCTCACGACAAACTAGG | 22.66 |
| IRIS_313-11747 | GCTCAAAGGAGATCG | ATCGTT | TGGGTCTGTATTCGTTGGAA | 1.19 |
| IRIS_313-11802 | - | ATCGTT | TGGGTCTGTATTCGTTGGAA | 29.04 |
| IRIS_313-11828 | ATATGCTAAGAGCTA | GCTAGG | CAAGCTCACGACAAACTAGG | 5.26 |
| IRIS_313-7688 | GCTCAAAGGAGATCG | ATCGTT | TGGGTCTGTATTCGTTGGAA | 0.58 |
| IRIS_313-7719 | GCTCAAAGGAGATCG | ATCGTT | TGGGTCTGTATTCGTTGGAA | 1.14 |
| IRIS_313-7725 | GCTCAAAGGAGATCG | ATCGTT | TGGGTCTGTATTCGTTGGAA | 3.55 |
| IRIS_313-8068 | GCTCAAAGGAGATCG | ATCGTT | - | 6.34 |
| IRIS_313-8069 | GCTCAAAGGAGATCG | ATCGTT | TGGGTCTGTATTCGTTGGAA | 2.25 |
| IRIS_313-8161 | GCTCAAAGGAGATCG | ATCGTT | - | 12.78 |
| IRIS_313-8288 | GCTCAAAGGAGATCG | ATCGTT | TGGGTCTGTATTCGTTGGAA | 52.33 |
| IRIS_313-8303 | GCTCAAAGGAGATCG | ATCGTT | TGGGTCTGTATTCGTTGGAA | 2.56 |
| IRIS_313-8305 | GCTCAAAGGAGATCG | ATCGTT | TGGGTCTGTATTCGTTGGAA | 16.18 |
| IRIS_313-8315 | GCTCAAAGGAGATCG | ATCGTT | TGGGTCTGTATTCGTTGGAA | 40.78 |
| IRIS_313-8332 | GCTCAAAGGAGATCG | ATCGTT | TGGGTCTGTATTCGTTGGAA | 28.17 |
| IRIS_313-8368 | GCTCAAAGGAGATCG | ATCGTT | - | 29.35 |
| IRIS_313-8380 | ATATGCTAAGAGCTA | GCTAGG | CAAGCTCACGACAAACTAGG | 84.21 |
| IRIS_313-8383 | - | - | - | 43.08 |
| IRIS_313-8386 | ATATGCTAAGAGCTA | GCTAGG | TGGGTCTGTATTCGTTGGAA | 6.39 |
| IRIS_313-8392 | GCTCAAAGGAGATCG | ATCGTT | TGGGTCTGTATTCGTTGGAA | 12.67 |
| IRIS_313-8401 | - | - | - | 63.93 |
| IRIS_313-8405 | ATATGCTAAGAGCTA | GCTAGG | CAAGCTCACGACAAACTAGG | 36.46 |
| IRIS_313-8409 | - | ATCGTT | TGGGTCTGTATTCGTTGGAA | 7.58 |
| IRIS_313-8412 | GCTCAAAGGAGATCG | ATCGTT | - | 8.00 |
| IRIS_313-8414 | GCTCAAAGGAGATCG | ATCGTT | TGGGTCTGTATTCGTTGGAA | 17.89 |
| IRIS_313-8433 | ATATGCTAAGAGCTA | GCTAGG | - | 17.77 |
| IRIS_313-8435 | ATATGCTAAGAGCTA | GCTAGG | CAAGCTCACGACAAACTAGG | 51.63 |
| IRIS_313-8437 | ATATGCTAAGAGCTA | GCTAGG | CAAGCCCACGACAAACTAGG | 84.48 |
| IRIS_313-8450 | GCTCAAAGGAGATCG | ATCGTT | TGGGTCTGTATTCGTTGGAA | 4.43 |
| IRIS_313-8453 | ATATGCTAAGAGCTA | GCTAGG | TGGGTCTGTATTCGTTGGAA | 14.98 |
| IRIS_313-8454 | ATATGCTAAGAGCTA | GCTAGG | TGGTTCTGTATTCGTTGGAA | 8.48 |
| IRIS_313-8458 | GCTCAAAGGAGATCG | ATCGTT | TGGGTCTGTATTCGTTGGAA | 6.71 |
| IRIS_313-8509 | - | ATCGTT | TGGGTCTGTATTCGTTGGAA | 2.37 |
| IRIS_313-8514 | ATATGCTAAGAGCTA | GCTAGG | CAAGCTCACGACAAACTAGG | 20.98 |
| IRIS_313-8530 | ATATGCTAAGAGCTA | GCTAGG | CAAGCCCACGACAAACTAGG | 46.36 |
| IRIS_313-8536 | GCTCAAAGGAGATCG | ATCGTT | TGGGTCTGTATTCGTTGGAA | 9.37 |
| IRIS_313-8559 | - | GCTAGG | CAAGCTCACGACAAACTAGG | 8.72 |
| IRIS_313-8568 | GCTCAAAGGAGATCG | ATCGTT | TGGGTCTGTATTCGTTGGAA | 21.02 |
| IRIS_313-8571 | - | - | TGGGTCTGTATTCGTTGGAA | 3.88 |
| IRIS_313-8585 | - | - | TGGGTCTGTATTCGTTGGAA | 40.12 |
| IRIS_313-8586 | ATATGCTAAGAGCTA | GCTAGG | TGGGTCTGTATTCGTTGGAA | 87.75 |
| IRIS_313-8595 | GCTCAAAGGAGATCG | ATCGTT | TGGGTCTGTATTCGTTGGAA | 75.83 |
| IRIS_313-8603 | ATATGCTAAGAGCTA | GCTAGG | TGGTTCTGTATTCGTTGGAA | 93.84 |
| IRIS_313-8614 | GCTCAAAGGAGATCG | ATCGTT | TGGGTCTGTATTCGTTGGAA | 0.58 |
| IRIS_313-8616 | GCTCAAAGGAGATCG | ATCGTT | TGGGTCTGTATTCGTTGGAA | 1.05 |
| IRIS_313-8621 | - | ATCGTT | TGGGTCTGTATTCGTTGGAA | 16.71 |
| IRIS_313-8622 | ATTTGCTAAGAGCTA | GCTAGG | TGGGTCTGTATTCGTTGGAA | 40.04 |

Table S4

| Accession IDs | New_gene | LOC_Os05g09520 | LOC_Os05g09530 | PGC |
|---|---|---|---|---|
| IRIS_313-8631 | ATATGCTAAGAGCTA | GCTAGG | CAAGCTCACGACAAACTAGG | 13.44 |
| IRIS_313-8632 | GCTCAAAGGAGATCG | ATCGTT | TGGGTCTGTATTCGTTGGAA | 35.79 |
| IRIS_313-8638 | ATATGCTAAGAGCTA | GCTAGG | TGGTTCTGTATTCGTTGGAA | 92.82 |
| IRIS_313-8643 | ATATGCTAAGAGCTA | GCTAGG | CAAGCTCACGACAAACTAGG | 2.67 |
| IRIS_313-8645 | ATATGCTAAGAGCTA | GCTAGG | CAAGCTCACGACAAACTAGG | 34.17 |
| IRIS_313-8647 | - | - | - | 6.21 |
| IRIS_313-8659 | ATATGCTAAGAGCTA | GCTAGG | TGGGTCTGTATTCGTTGGAA | 39.73 |
| IRIS_313-8660 | - | ATCGTT | TGGGTCTGTATTCGTTGGAA | 10.65 |
| IRIS_313-8674 | GCTCAAAGGAGATCG | ATCGTT | TGGGTCTGTATTCGTTGGAA | 0.82 |
| IRIS_313-8697 | - | ATCGTT | TGGGTCTGTATTCGTTGGAA | 0.71 |
| IRIS_313-8699 | ATATGCTAAGAGCTA | GCTAGG | **TGGGTCTGTGTTCGTTGGAA** | 12.43 |
| IRIS_313-8704 | - | - | - | 5.31 |
| IRIS_313-8717 | ATATGCTAAGAGCTA | GCTAGG | TGGGTCTGTATTCGTTGGAA | 47.75 |
| IRIS_313-8722 | ATATGCTAAGAGCTA | GCTAGG | TGGTTCTGTATTCGTTGGAA | 87.13 |
| IRIS_313-8725 | GCTCAAAGGAGATCG | ATCGTT | TGGGTCTGTATTCGTTGGAA | 52.53 |
| IRIS_313-8727 | ATATGCTAAGAGCTA | - | - | 17.42 |
| IRIS_313-8731 | ATATGCTAAGAGCTA | GCTAGG | CAAGCTCACGACAAACTAGG | 22.60 |
| IRIS_313-8732 | GCTCAAAGGAGATCG | ATCGTT | TGGGTCTGTATTCGTTGGAA | 3.76 |
| IRIS_313-8733 | ATATGCTAAGAGCTA | GCTAGG | CAAGCCCACGACAAACTAGG | 24.39 |
| IRIS_313-8737 | GCTCAAAGGAGATCG | ATCGTT | **TGGGTCTGTGTTCGTTGGAA** | 24.38 |
| IRIS_313-8743 | ATATGCTAAGAGCTA | GCTAGG | CAAGCTCACGACAAACTAGG | 33.54 |
| IRIS_313-8744 | ATATGCTAAGAGCTA | GCTAGG | TGGGTCTGTATTCGTTGGAA | 54.46 |
| IRIS_313-8751 | GCTCAAAGGAGATCG | ATCGTT | TGGGTCTGTATTCGTTGGAA | 2.32 |
| IRIS_313-8754 | ATATGCTAAGAGCTA | GCTAGG | CAAGCTCACGACAAACTAGG | 35.63 |
| IRIS_313-8757 | ATATGCTAAGAGCTA | - | TGGGTCTGTATTCGTTGGAA | 31.68 |
| IRIS_313-8781 | GCTCAAAGGAGATCG | ATCGTT | TGGGTCTGTATTCGTTGGAA | 5.20 |
| IRIS_313-8785 | GCTCAAAGAGAGCTA | GCTAGG | TGGTTCTGTATTCGTTGGAA | 3.67 |
| IRIS_313-8796 | GCTCAAAGGAGATCG | ATCGTT | TGGGTCTGTATTCGTTGGAA | 4.13 |
| IRIS_313-8812 | ATATGCTAAGAGCTA | GCTAGG | CAAGCTCACGACAAACTAGG | 41.84 |
| IRIS_313-8831 | - | GCTAGG | CAAGCTCACGACAAACTAGG | 17.93 |
| IRIS_313-8844 | - | ATCGTT | TGGGTCTGTATTCGTTGGAA | 15.66 |
| IRIS_313-8846 | ATATGCTAAGAGCTA | GCTAGG | - | 41.54 |
| IRIS_313-8850 | - | GCTAGG | TGGGTCTGTATTCGTTGGAA | 37.35 |
| IRIS_313-8854 | - | ATCGTT | - | 21.55 |
| IRIS_313-8889 | - | - | CAAGCTCACGACAAACTAGG | 9.61 |
| IRIS_313-8900 | GCTCAAAGGAGATCG | ATCGTT | TGGGTCTGTATTCGTTGGAA | 9.42 |
| IRIS_313-8903 | GCTCAAAGGAGATCG | ATCGTT | - | 8.00 |
| IRIS_313-8914 | ATATGCTAAGAGCTA | GCTAGG | TGGTTCTGTATTCGTTGGAA | 7.98 |
| IRIS_313-8920 | **GCTCAAAGGAGATCG** | ATCGTT | TGGGTCTGTATTCGTTGGAA | 3.85 |
| IRIS_313-8924 | ATATGCTAAGAGCTA | GCTAGG | CAAGCTCACGACAAACTAGG | 10.06 |
| IRIS_313-8925 | ATATGCTAAGAGCTA | GCTAGG | CAAGCCCACGACAAACTAGG | 42.54 |
| IRIS_313-8930 | ATATGCTAAGAGCTA | GCTAGG | CAAGCCCACGACAAACTAGG | 53.00 |
| IRIS_313-8932 | GCTCAAAGGAGATCG | ATCGTT | TGGGTCTGTATTCGTTGGAA | 2.30 |
| IRIS_313-8935 | GCTCAAAGGAGATCG | ATCGTT | TGGGTCTGTATTCGTTGGAA | 2.68 |
| IRIS_313-8940 | ATATGCTAAGAGCTA | GCTAGG | TGGTTCTGTATTCGTTGGAA | 28.46 |
| IRIS_313-8946 | ATATGCTAAGAGCTA | GCTAGG | TGGTTCTGTATTCGTTGGAA | 78.73 |
| IRIS_313-8948 | ATATGCTAAGAGCTA | GCTAGG | TGGTTCTGTATTCGTTGGAA | 4.91 |
| IRIS_313-8956 | **GCTCGCTGGGGGCCG** | **GCCGGT** | - | 7.02 |
| IRIS_313-8967 | - | ATCGTT | TGGGTCTGTATTCGTTGGAA | 30.13 |
| IRIS_313-8968 | ATATGCTAAGAGCTA | GCTAGG | CAAGCTCACGACAAACTAGG | 29.56 |
| IRIS_313-8980 | ATATGCTAAGAGCTA | GCTAGG | TGGTTCTGTATTCGTTGGAA | 90.52 |
| IRIS_313-8982 | GCTCAAAGGAGATCG | ATCGTT | - | 9.44 |
| IRIS_313-8986 | - | - | **TGGGTCTGTGTTCGTTGGAA** | 12.38 |
| IRIS_313-8987 | GCTCAAAGGAGATCG | ATCGTT | TGGGTCTGTATTCGTTGGAA | 2.58 |

Table S4

| Accession IDs | New_gene | LOC_Os05g09520 | LOC_Os05g09530 | PGC |
|---|---|---|---|---|
| IRIS_313-8988 | GCTCAAAGGAGATCG | ATCGTT | TGGGTCTGTATTCGTTGGAA | 1.98 |
| IRIS_313-8994 | GCTCAAAGGAGATCG | ATCGTT | TGGGTCTGTATTCGTTGGAA | 60.56 |
| IRIS_313-9005 | ATATGCTAAGAGCTA | GCTAGG | TGGGTCTGTATTCGTTGGAA | 33.83 |
| IRIS_313-9017 | ATATGCTAAGAGCTA | GCTAGG | TGGTTCTGTATTCGTTGGAA | 48.05 |
| IRIS_313-9023 | ATATGCTAAGAGCTA | GCTAGG | **TGGGTCTGTGTTCGTTGGAA** | 6.91 |
| IRIS_313-9032 | GCTCAAAGGAGATCG | ATCGTT | TGGGTCTGTATTCGTTGGAA | 2.18 |
| IRIS_313-9044 | GCTCAAAGGAGATCG | ATCGTT | TGGGTCTGTATTCGTTGGAA | 1.78 |
| IRIS_313-9065 | ATATGCTAAGAGCTA | GCTAGG | - | 27.12 |
| IRIS_313-9066 | GCTCAAAGGAGATCG | ATCGTT | TGGGTCTGTATTCGTTGGAA | 1.47 |
| IRIS_313-9067 | GCTCAAAGGAGATCG | ATCGTT | TGGGTCTGTATTCGTTGGAA | 39.85 |
| IRIS_313-9072 | GCTCAAAGGAGATCG | ATCGTT | TGGGTCTGTATTCGTTGGAA | 5.39 |
| IRIS_313-9111 | GCTCAAAGGAGATCG | ATCGTT | TGGGTCTGTATTCGTTGGAA | 1.18 |
| IRIS_313-9119 | GCTCAAAGGAGATCG | ATCGTT | - | 3.02 |
| IRIS_313-9139 | GCTCAAAGGAGATCG | ATCGTT | TGGGTCTGTATTCGTTGGAA | 3.52 |
| IRIS_313-9148 | ATATGCTAAGAGCTA | GCTAGG | CAAGCCCACGACAAACTAGG | 22.56 |
| IRIS_313-9156 | GCTCAAAGGAGATCG | ATCGTT | TGGGTCTGTATTCGTTGGAA | 6.33 |
| IRIS_313-9174 | - | ATCGTT | TGGGTCTGTATTCGTTGGAA | 43.79 |
| IRIS_313-9184 | ATATGCTAAGAGCTA | GCTAGG | CAAGCTCACGACAAACTAGG | 10.28 |
| IRIS_313-9187 | - | - | - | 22.56 |
| IRIS_313-9190 | - | - | CAAGCTCACGACAAACTAGG | 0.59 |
| IRIS_313-9197 | ATATGCTAAGAGCTA | GCTAGG | - | 65.23 |
| IRIS_313-9198 | GCTCAAAGGAGATCG | ATCGTT | TGGGTCTGTATTCGTTGGAA | 81.53 |
| IRIS_313-9204 | ATATGCTAAGAGCTA | - | TGGTTCTGTATTCGTTGGAA | 17.19 |
| IRIS_313-9218 | - | ATCGTT | - | 22.12 |
| IRIS_313-9227 | GCTCAAAGGAGATCG | ATCGTT | TGGGTCTGTATTCGTTGGAA | 4.51 |
| IRIS_313-9253 | ATTTGCTAAGAGCTA | GCTAGG | CAAGCTCACGACAAACTAGG | 41.75 |
| IRIS_313-9256 | - | GCTAGG | CAAGCCCACGACAAACTAGG | 35.09 |
| IRIS_313-9259 | GCTCAAAGGAGATCG | - | TGGGTCTGTATTCGTTGGAA | 7.33 |
| IRIS_313-9262 | GCTCAAAGGAGATCG | ATCGTT | TGGGTCTGTATTCGTTGGAA | 7.47 |
| IRIS_313-9271 | GCTCAAAGGAGATCG | ATCGTT | - | 5.74 |
| IRIS_313-9281 | ATATGCTAAGAGCTA | GCTAGG | TGGTTCTGTATTCGTTGGAA | 18.29 |
| IRIS_313-9285 | ATATGCTAAGAGCTA | GCTAGG | - | 21.72 |
| IRIS_313-9286 | GCTCAAAGAGAGCTA | GCTAGG | TGGTTCTGTATTCGTTGGAA | 2.86 |
| IRIS_313-9287 | GCTCAAAGGAGATCG | ATCGTT | TGGGTCTGTATTCGTTGGAA | 40.83 |
| IRIS_313-9288 | GCTCAAAGGAGATCG | ATCGTT | TGGGTCTGTATTCGTTGGAA | 8.18 |
| IRIS_313-9313 | GCTCAAAGGAGATCG | ATCGTT | TGGGTCTGTATTCGTTGGAA | 18.58 |
| IRIS_313-9314 | GCTCAAAGGAGATCG | ATCGTT | TGGGTCTGTATTCGTTGGAA | 28.95 |
| IRIS_313-9317 | ATATGCTAAGAGCTA | - | TGGGTCTGTATTCGTTGGAA | 8.11 |
| IRIS_313-9320 | ATATGCTAAGAGCTA | GCTAGG | - | 100.00 |
| IRIS_313-9324 | ATATGCTAAGAGCTA | GCTAGG | TGGTTCTGTATTCGTTGGAA | 53.88 |
| IRIS_313-9325 | GCTCAAAGGAGATCG | ATCGTT | TGGGTCTGTATTCGTTGGAA | 13.53 |
| IRIS_313-9348 | GCTCAAAGGAGATCG | ATCGTT | TGGGTCTGTATTCGTTGGAA | 1.57 |
| IRIS_313-9351 | - | ATCGTT | TGGGTCTGTATTCGTTGGAA | 23.12 |
| IRIS_313-9360 | GCTCAAAGGAGATCG | ATCGTT | TGGGTCTGTATTCGTTGGAA | 2.71 |
| IRIS_313-9372 | ATATGCTAAGAGCTA | GCTAGG | TGGTTCTGTATTCGTTGGAA | 25.13 |
| IRIS_313-9384 | GCTCAAAGGAGATCG | ATCGTT | - | 0.51 |
| IRIS_313-9388 | ATATGCTAAGAGCTA | GCTAGG | CAAGCTCACGACAAACTAGG | 40.26 |
| IRIS_313-9400 | GCTCAAAGGAGATCG | ATCGTT | - | 17.30 |
| IRIS_313-9403 | GCTCAAAGGAGATCG | ATCGTT | TGGGTCTGTATTCGTTGGAA | 2.50 |
| IRIS_313-9424 | ATATGCTAAGAGCTA | GCTAGG | TGGTTCTGTATTCGTTGGAA | 15.62 |
| IRIS_313-9427 | GCTCAAAGGAGATCG | ATCGTT | TGGGTCTGTATTCGTTGGAA | 41.50 |
| IRIS_313-9429 | - | ATCGTT | TGGGTCTGTATTCGTTGGAA | 7.20 |
| IRIS_313-9433 | - | ATCGTT | TGGGTCTGTATTCGTTGGAA | 51.43 |
| IRIS_313-9451 | GCTCAAAGAGAGCTA | GCTAGG | TGGTTCTGTATTCGTTGGAA | 14.68 |

Table S4

| Accession IDs | New_gene | LOC_Os05g09520 | LOC_Os05g09530 | PGC |
|---|---|---|---|---|
| IRIS_313-9461 | - | ATCGTT | TGGGTCTGTATTCGTTGGAA | 0.45 |
| IRIS_313-9469 | ATTTGCTAAGAGCTA | GCTAGG | CAAGCTCACGACAAACTAGG | 63.79 |
| IRIS_313-9472 | - | - | - | 13.82 |
| IRIS_313-9482 | GCTCAAAGGAGATCG | ATCGTT | TGGGTCTGTATTCGTTGGAA | 2.60 |
| IRIS_313-9484 | GCTCAAAGGAGATCG | ATCGTT | TGGGTCTGTATTCGTTGGAA | 6.45 |
| IRIS_313-9492 | GCTCAAAGGAGATCG | ATCGTT | TGGGTCTGTATTCGTTGGAA | 37.36 |
| IRIS_313-9503 | GCTCAAAGGAGATCG | ATCGTT | TGGGTCTGTATTCGTTGGAA | 8.36 |
| IRIS_313-9505 | GCTCAAAGGAGATCG | ATCGTT | TGGGTCTGTATTCGTTGGAA | 7.65 |
| IRIS_313-9522 | ATATGCTAAGAGCTA | GCTAGG | CAAGCTCACGACAAACTAGG | 36.93 |
| IRIS_313-9533 | - | ATCGTT | TGGGTCTGTATTCGTTGGAA | 1.81 |
| IRIS_313-9547 | GCTCAAAGGAGATCG | ATCGTT | TGGGTCTGTATTCGTTGGAA | 22.84 |
| IRIS_313-9551 | GCTCAAAGGAGATCG | ATCGTT | TGGGTCTGTATTCGTTGGAA | 6.56 |
| IRIS_313-9557 | ATATGCTAAGAGCTA | GCTAGG | TGGGTCTGTATTCGTTGGAA | 28.36 |
| IRIS_313-9560 | ATATGCTAAGAGCTA | GCTAGG | - | 40.11 |
| IRIS_313-9566 | GCTCAAAGGAGATCG | ATCGTT | TGGGTCTGTATTCGTTGGAA | 4.89 |
| IRIS_313-9570 | GCTCAAAGGAGATCG | ATCGTT | TGGGTCTGTATTCGTTGGAA | 1.45 |
| IRIS_313-9572 | GCTCAAAGGAGATCG | ATCGTT | TGGGTCTGTATTCGTTGGAA | 15.25 |
| IRIS_313-9574 | GCTCAAAGGAGATCG | ATCGTT | TGGGTCTGTATTCGTTGGAA | 3.35 |
| IRIS_313-9590 | GCTCAAAGGAGATCG | ATCGTT | TGGGTCTGTATTCGTTGGAA | 53.99 |
| IRIS_313-9593 | GCTCAAAGGAGATCG | ATCGTT | TGGGTCTGTATTCGTTGGAA | 29.40 |
| IRIS_313-9594 | ATATGCTAAGAGCTA | GCTAGG | CAAGCCCACGACAAACTAGG | 89.99 |
| IRIS_313-9602 | GCTCAAAGGAGATCG | ATCGTT | TGGGTCTGTATTCGTTGGAA | 2.56 |
| IRIS_313-9604 | GCTCAAAGGAGATCG | ATCGTT | TGGGTCTGTATTCGTTGGAA | 2.91 |
| IRIS_313-9605 | ATATGCTAAGAGCTA | GCTAGG | CAAGCTCACGACAAACTAGG | 8.44 |
| IRIS_313-9609 | ATATGCTAAGAGCTA | GCTAGG | TGGTTCTGTATTCGTTGGAA | 8.04 |
| IRIS_313-9611 | - | - | CAAGCTCACGACAAACTAGG | 4.30 |
| IRIS_313-9617 | ATATGCTAAGAGCTA | GCTAGG | TGGGTCTGTATTCGTTGGAA | 68.45 |
| IRIS_313-9623 | GCTCAAAGGAGATCG | ATCGTT | - | 4.22 |
| IRIS_313-9634 | GCTCAAAGGAGATCG | ATCGTT | TGGGTCTGTATTCGTTGGAA | 76.20 |
| IRIS_313-9696 | GCTCAAAGGAGATCG | ATCGTT | TGGGTCTGTATTCGTTGGAA | 1.05 |
| IRIS_313-9705 | ATTTGCTAAGAGCTA | GCTAGG | - | 18.71 |
| IRIS_313-9706 | ATTTGCTAAGAGCTA | GCTAGG | CAAGCTCACGACAAACTAGG | 14.98 |
| IRIS_313-9708 | ATATGCTAAGAGCTA | GCTAGG | CAAGCTCACGACAAACTAGG | 4.31 |
| IRIS_313-9723 | ATATGCTAAGAGCTA | GCTAGG | CAAGCTCACGACAAACTAGG | 38.42 |
| IRIS_313-9727 | ATATGCTAAGAGCTA | GCTAGG | TGGTTCTGTATTCGTTGGAA | 37.02 |
| IRIS_313-9730 | ATATGCTAAGAGCTA | GCTAGG | CAAGCTCACGACAAACTAGG | 26.77 |
| IRIS_313-9732 | - | - | - | 8.08 |
| IRIS_313-9740 | GCTCAAAGGAGATCG | ATCGTT | - | 48.03 |
| IRIS_313-9758 | ATTTGCTAAGAGCTA | GCTAGG | CAAGCCCACGACAAACTAGG | 19.15 |
| IRIS_313-9767 | - | **GCCGGT** | **TGGGTCTGTGTTCGTTGGAA** | 9.09 |
| IRIS_313-9791 | - | GCTAGG | - | 34.96 |
| IRIS_313-9795 | GCTCAAAGGAGATCG | ATCGTT | CAAGCCCACGACAAACTAGG | 9.49 |
| IRIS_313-9831 | ATATGCTAAGAGCTA | GCTAGG | - | 34.49 |
| IRIS_313-9841 | GCTCAAAGGAGATCG | ATCGTT | TGGGTCTGTATTCGTTGGAA | 2.32 |
| IRIS_313-9862 | ATATGCTAAGAGCTA | GCTAGG | CAAGCTCACGACAAACTAGG | 69.82 |
| IRIS_313-9867 | ATATGCTAAGAGCTA | GCTAGG | TGGTTCTGTATTCGTTGGAA | 67.46 |
| IRIS_313-9882 | ATATGCTAAGAGCTA | GCTAGG | CAAGCTCACGACAAACTAGG | 88.94 |
| IRIS_313-9898 | GCTCAAAGAGAGCTA | - | TGGTTCTGTATTCGTTGGAA | 11.28 |
| IRIS_313-9917 | ATATGCTAAGAGCTA | GCTAGG | TGGTTCTGTATTCGTTGGAA | 9.71 |
| IRIS_313-9922 | GCTCAAAGGAGATCG | ATCGTT | CAAGCCCACGACAAACTAGG | 5.62 |
| IRIS_313-9924 | ATATGCTAAGAGCTA | GCTAGG | TGGTTCTGTATTCGTTGGAA | 15.36 |
| IRIS_313-9925 | GCTCAAAGGAGATCG | ATCGTT | CAAGCCCACGACAAACTAGG | 5.45 |
| IRIS_313-9940 | **GCTCGCTGGGGGCCG** | **GCCGGT** | **TGGGTCTGTGTTCGTTGGAA** | 8.65 |
| IRIS_313-9944 | GCTCAAAGGAGATCG | ATCGTT | - | 3.67 |

Table S4

| Accession IDs | New_gene | LOC_Os05g09520 | LOC_Os05g09530 | PGC |
|---|---|---|---|---|
| IRIS_313-9953 | GCTCAAAGGAGATCG | ATCGTT | TGGGTCTGTATTCGTTGGAA | 10.01 |
| IRIS_313-9966 | **GCTCGCTGGGGGCCG** | **GCCGGT** | **TGGGTCTGTGTTCGTTGGAA** | 6.97 |
| IRIS_313-9968 | ATATGCTAAGAGCTA | GCTAGG | - | 30.61 |
| IRIS_313-9969 | ATATGCTAAGAGCTA | GCTAGG | TGGTTCTGTATTCGTTGGAA | 90.56 |
| IRIS_313-9970 | ATATGCTAAGAGCTA | GCTAGG | CAAGCTCACGACAAACTAGG | 18.53 |
| IRIS_313-9976 | ATTTGCTAAGAGCTA | GCTAGG | CAAGCTCACGACAAACTAGG | 29.27 |

**Table S5: Single locus GWAS for SNPs detected against ZS97 reference genome with corrosponding orthologous Ids in MH63 and Nipponbare.**

| Position | Chr | Ref | Alt | Beta | -log10 p-value | Region | Gene | Orthologoues_MH63 | Orthologoues_Nipponbare |
|---|---|---|---|---|---|---|---|---|---|
| 4104137 | 5 | G | C | 0.30 | 5.70 | Upstream | ZS05g0093300 | MH05g0082900 | LOC_Os05g08670 |
| 4599654 | 5 | T | A | -0.41 | 11.70 | Upstream | ZS05g0110000 (GW5) | MH05g0093000 (GW5) | LOC_Os05g09520 (GW5) |
| 4599671 | 5 | A | G | -0.48 | 14.84 | Upstream | ZS05g0110000 | MH05g0093000 | LOC_Os05g09520 |
| 4600629 | 5 | A | G | -0.46 | 11.23 | Upstream | ZS05g0110000 | MH05g0093000 | LOC_Os05g09520 |
| 4602339 | 5 | G | T | -0.45 | 13.14 | Upstream | ZS05g0110000 | MH05g0093000 | LOC_Os05g09520 |
| 4607243 | 5 | A | T | -0.43 | 12.39 | Downstream | ZS05g0110000 | MH05g0093000 | LOC_Os05g09520 |
| 4607689 | 5 | A | T | -0.45 | 13.41 | Downstream | ZS05g0110000 | MH05g0093000 | LOC_Os05g09520 |
| 4607936 | 5 | A | G | -0.43 | 12.29 | Downstream | ZS05g0110000 | MH05g0093000 | LOC_Os05g09520 |
| 4608886 | 5 | T | C | -0.43 | 12.40 | Downstream | ZS05g0110000 | MH05g0093000 | LOC_Os05g09520 |
| 4609180 | 5 | T | C | -0.43 | 12.41 | Downstream | ZS05g0110000 | MH05g0093000 | LOC_Os05g09520 |
| 4609667 | 5 | C | A | -0.41 | 11.34 | Downstream | ZS05g0110000 | MH05g0093000 | LOC_Os05g09520 |
| 4609747 | 5 | A | G | -0.40 | 10.65 | Downstream | ZS05g0110000 | MH05g0093000 | LOC_Os05g09520 |
| 4609824 | 5 | C | T | -0.40 | 10.48 | Downstream | ZS05g0110000 | MH05g0093000 | LOC_Os05g09520 |
| 4609854 | 5 | G | A | -0.41 | 10.98 | Downstream | ZS05g0110000 | MH05g0093000 | LOC_Os05g09520 |
| 4609910 | 5 | G | A | -0.41 | 11.03 | Downstream | ZS05g0110000 | MH05g0093000 | LOC_Os05g09520 |
| 4610087 | 5 | C | G | -0.42 | 11.68 | Downstream | ZS05g0110000 | MH05g0093000 | LOC_Os05g09520 |
| 4610188 | 5 | C | T | -0.40 | 10.33 | Downstream | ZS05g0110000 | MH05g0093000 | LOC_Os05g09520 |
| 4611979 | 5 | C | T | -0.40 | 10.53 | Intergenic | ZS05g0110000-ZS05g0110100 | MH05g0093000-MH05g0093100 | LOC_Os05g09520-LOC_Os05g09530 |
| 4612359 | 5 | C | T | -0.40 | 10.33 | Intergenic | ZS05g0110000-ZS05g0110100 | MH05g0093000-MH05g0093100 | LOC_Os05g09520-LOC_Os05g09530 |
| 4613099 | 5 | C | T | -0.37 | 8.69 | Intergenic | ZS05g0110000-ZS05g0110100 | MH05g0093000-MH05g0093100 | LOC_Os05g09520-LOC_Os05g09530 |
| 4613339 | 5 | T | C | -0.36 | 8.10 | Intergenic | ZS05g0110000-ZS05g0110100 | MH05g0093000-MH05g0093100 | LOC_Os05g09520-LOC_Os05g09530 |
| 4613365 | 5 | T | C | -0.35 | 7.67 | Intergenic | ZS05g0110000-ZS05g0110100 | MH05g0093000-MH05g0093100 | LOC_Os05g09520-LOC_Os05g09530 |
| 4613902 | 5 | A | G | -0.35 | 7.72 | Intergenic | ZS05g0110000-ZS05g0110100 | MH05g0093000-MH05g0093100 | LOC_Os05g09520-LOC_Os05g09530 |
| 4613924 | 5 | G | A | -0.36 | 8.43 | Intergenic | ZS05g0110000-ZS05g0110100 | MH05g0093000-MH05g0093100 | LOC_Os05g09520-LOC_Os05g09530 |
| 4613931 | 5 | G | A | -0.34 | 7.44 | Intergenic | ZS05g0110000-ZS05g0110100 | MH05g0093000-MH05g0093100 | LOC_Os05g09520-LOC_Os05g09530 |
| 4613987 | 5 | C | A | -0.27 | 5.14 | Intergenic | ZS05g0110000-ZS05g0110100 | MH05g0093000-MH05g0093100 | LOC_Os05g09520-LOC_Os05g09530 |
| 4614060 | 5 | A | G | -0.35 | 8.01 | Intergenic | ZS05g0110000-ZS05g0110100 | MH05g0093000-MH05g0093100 | LOC_Os05g09520-LOC_Os05g09530 |
| 4614276 | 5 | G | A | -0.34 | 7.48 | Intergenic | ZS05g0110000-ZS05g0110100 | MH05g0093000-MH05g0093100 | LOC_Os05g09520-LOC_Os05g09530 |
| 4614292 | 5 | C | T | -0.30 | 6.15 | Intergenic | ZS05g0110000-ZS05g0110100 | MH05g0093000-MH05g0093100 | LOC_Os05g09520-LOC_Os05g09530 |
| 4614299 | 5 | A | G | -0.34 | 7.50 | Intergenic | ZS05g0110000-ZS05g0110100 | MH05g0093000-MH05g0093100 | LOC_Os05g09520-LOC_Os05g09530 |
| 4614499 | 5 | T | C | -0.29 | 5.99 | Intergenic | ZS05g0110000-ZS05g0110100 | MH05g0093000-MH05g0093100 | LOC_Os05g09520-LOC_Os05g09530 |
| 4614515 | 5 | A | G | -0.30 | 6.24 | Intergenic | ZS05g0110000-ZS05g0110100 | MH05g0093000-MH05g0093100 | LOC_Os05g09520-LOC_Os05g09530 |
| 4615150 | 5 | A | G | -0.31 | 6.39 | Intergenic | ZS05g0110000-ZS05g0110100 | MH05g0093000-MH05g0093100 | LOC_Os05g09520-LOC_Os05g09530 |
| 4615250 | 5 | T | A | -0.37 | 8.88 | Intergenic | ZS05g0110000-ZS05g0110100 | MH05g0093000-MH05g0093100 | LOC_Os05g09520-LOC_Os05g09530 |
| 4615336 | 5 | T | C | -0.37 | 8.94 | Intergenic | ZS05g0110000-ZS05g0110100 | MH05g0093000-MH05g0093100 | LOC_Os05g09520-LOC_Os05g09530 |
| 4615445 | 5 | A | G | -0.36 | 8.30 | Intergenic | ZS05g0110000-ZS05g0110100 | MH05g0093000-MH05g0093100 | LOC_Os05g09520-LOC_Os05g09530 |
| 4615745 | 5 | A | G | -0.37 | 8.74 | Intergenic | ZS05g0110000-ZS05g0110100 | MH05g0093000-MH05g0093100 | LOC_Os05g09520-LOC_Os05g09530 |
| 4616584 | 5 | T | A | -0.37 | 8.36 | Intergenic | ZS05g0110000-ZS05g0110100 | MH05g0093000-MH05g0093100 | LOC_Os05g09520-LOC_Os05g09530 |

| Position | Chr | Ref | Alt | Beta | -log10 p-value | Region | Gene | Orthologoues_MH63 | Orthologoues_Nipponbare |
|---|---|---|---|---|---|---|---|---|---|
| 4617119 | 5 | A | T | -0.38 | 9.09 | Intergenic | ZS05g0110000-ZS05g0110100 | MH05g0093000-MH05g0093100 | LOC_Os05g09520-LOC_Os05g09530 |
| 5054322 | 5 | A | C | -0.31 | 5.27 | Downstream | ZS05g0115200 | MH05g0098900 | LOC_Os05g10690 |
| 5069547 | 5 | T | C | -0.27 | 5.16 | Upstream | ZS05g0115300 | MH05g0099000 | LOC_Os05g10700 |

# Table S6

**Table S6: Single locus GWAS for SNPs detected against MH63 reference genome with corrosponding Orthologous IDs in ZS97 and Nipponbare**

| Position | Chr | Ref | Alt | Beta | -log10 p-value | Effect | Gene | Orthologous_ZS97 | Orthologous_Nipponbare |
|---|---|---|---|---|---|---|---|---|---|
| 5012096 | 5 | T | C | 0.29 | 5.28 | Synonymous | MH05g0089300 | Not present | LOC_Os05g09200 |
| 5107094 | 5 | A | G | 0.35 | 8.16 | Upstream | MH05g0091600 | ZS05g0108900 | LOC_Os05g09360 |
| 5192872 | 5 | T | C | 0.36 | 8.67 | Upstream | MH05g0092200 | Not present | LOC_Os05g09470 |
| 5269365 | 5 | T | C | -0.32 | 7.30 | Upstream | MH05g0093300 | ZS05g0110300 | LOC_Os05g09550 |
| 5298677 | 5 | C | T | -0.30 | 6.63 | Upstream | MH05g0094100 | ZS05g0110700 | LOC_Os05g09630 |
| 5302192 | 5 | T | C | -0.30 | 6.60 | Upstream | MH05g0094100 | ZS05g0110700 | LOC_Os05g09630 |

S

# Table S7

**Table S7: Genes/QTLs characterized in the mutants influencing the grain chalkiness.**

| Gene/QTL | Chr | Locus ID | Start-end positions (Mb) | Effect | Reference |
|---|---|---|---|---|---|
| *SUBSTANDARD STARCH GRAIN4 (SSG4)* | 1 | LOC_Os01g08420 | 4.13-4.14 | Chalk endosperm | (Matsushima *et al.*, 2014) |
| *OsAPL2* | 1 | LOC_Os01g44220 | 25.35-25.36 | Chalky endosperm | (Zhang *et al.*, 2012) |
| *SBEIIB* | 2 | LOC_Os02g32660 | 19.37-19.36 | Amylose-Extender Mutation in Rice | (Butardo *et al.*, 2011; Nishi *et al.*, 2001) |
| *gif1 (grain incomplete Filling 1)/OsCIN2* | 4 | LOC_Os04g33740 | 20.42-20.43 | Abnormally developed and loosely Packed starch granules | (Wang *et al.*, 2008) |
| *flo2 (FLOURY ENDOSPERM2)* | 4 | LOC_Os04g55230 | 32.84-32.85 | Affects storage substance accumulation In the endosperm | (She *et al.*, 2010) |
| *Flo4 (OsPPDKB)* | 5 | LOC_Os05g33570 | 19.72-19.74 | Affects metabolism and starch structure In the endosperm | (Kang *et al.*, 2005; Zhang *et al.*, 2018) |
| *Flo1* | 5 | NA* | 17.70-20.70 | floury endosperm | (Mo *et al.*, 2013a) |
| *flo7* (*t*) | 5 | NA* | 19.33–19.86 | white-core floury endosperm | (Mo *et al.*, 2013b) |
| *OsSSIII/flo5* | 8 | LOC_Os08g09230 | 5.35-5.36 | white-core floury endosperm | (Fujita *et al.*, 2007; Ryoo *et al.*, 2007) |
| *OsVPS22* | 9 | LOC_Os09g36020 | 20.75-20.75 | chalky endosperm | (Zhang *et al.*, 2013) |
| *Flo7 (FLOURY ENDOSPERM7)* | 10 | LOC_Os10g32680 | 17.11-17.12 | chalky or floury endosperm | (Zhang *et al.*, 2015) |

**The gene ID was not detected.*

## References :


**Butardo V, Jr., Fitzgerald MA, Bird AR, Gidley MJ, Flanagan BM, Larroque O, Resurreccion AP, Laidlaw HK, Jobling SA, Morell MK, Rahman S** 2011. Impact of down-regulation of starch branching enzyme IIb in rice by artificial microRNA- and hairpin RNA-mediated RNA silencing. *Journal of Experimental Botany* **62**, 4927–4941.

**Fujita N, Yoshida M, Kondo T, Saito K, Utsumi Y, Tokunaga T, Nishi A, Satoh H, Park J-H, Jane J-L** 2007. Characterization of SSIIIa-deficient mutants of rice: the function of SSIIIa and pleiotropic effects by SSIIIa deficiency in the rice endosperm. *Plant Physiology* **144**, 2009-2023.

Table S7

**Kang HG, Park S, Matsuoka M, An G**. 2005. White-core endosperm floury endosperm-4 in rice is generated By knockout mutations in the C4-type pyruvate orthophosphate dikinase gene (OsPPDKB). *The Plant Journal* **42**, 901-911.

**Matsushima R, Maekawa M, Kusano M, Kondo H, Fujita N, Kawagoe Y, Sakamoto W**. 2014. Amyloplast-localized SUBSTANDARD STARCH GRAIN4 protein influences the size of starch grains in rice endosperm. *Plant Physiology* **164**, 623-636.

**Mo Y-J, Jeung J-U, Kang K-H, Lee J-S, Kim B-K**. 2013a. Genetic Analysis on Floury Endosperm Characteristics of Namil (SA)-flo1, a Japonica Rice Mutant Line. *Korean Journal of Crop Science* **58**, 283-291.

**Mo Y-J, Jeung J-U, Shin Y-S, Park CS, Kang K-H, Kim B-K**. 2013b. Agronomic and genetic analysis of Suweon 542, a rice floury mutant line suitable for dry milling. *Rice* **6**, 37.

**Nishi A, Nakamura Y, Tanaka N, Satoh H**. 2001. Biochemical and genetic analysis of the effects of amylose -extender mutation in rice endosperm. *Plant Physiology* **127**, 459-472.

**Ryoo N, Yu C, Park C-S, Baik M-Y, Park IM, Cho M-H, Bhoo SH, An G, Hahn T-R, Jeon J-S**. 2007. Knockout of a starch synthase gene OsSSIIIa/Flo5 causes white-core floury endosperm in rice (Oryza sativa L.). *Plant Cell Reports* **26**, 1083-1095.

**She K-C, Kusano H, Koizumi K, Yamakawa H, Hakata M, Imamura T, Fukuda M, Naito N, Tsurumaki Y, Yaeshima M**. 2010. A novel factor FLOURY ENDOSPERM2 is involved in regulation of rice grain size and starch quality. *The Plant Cell* , tpc. 109.070821.

**Wang E, Wang J, Zhu X, Hao W, Wang L, Li Q, Zhang L, He W, Lu B, Lin H**. 2008. Control of rice Grain-filling and yield by a gene with a potential signature of domestication. *Nature Genetics* **40**, 1370.
